# A self‐propagating, barcoded transposon system for the dynamic rewiring of genomic networks

**DOI:** 10.15252/msb.202211398

**Published:** 2023-03-27

**Authors:** Max A English, Miguel A Alcantar, James J Collins

**Affiliations:** ^1^ Department of Biological Engineering Massachusetts Institute of Technology (MIT) Cambridge MA USA; ^2^ Institute for Medical Engineering and Science, MIT Cambridge MA USA; ^3^ Wyss Institute for Biologically Inspired Engineering Harvard University Boston MA USA; ^4^ Broad Institute of MIT and Harvard Cambridge MA USA

**Keywords:** functional genomics, gene regulatory network, laboratory evolution, Tn‐Seq, transposon, Biotechnology & Synthetic Biology, Methods & Resources

## Abstract

In bacteria, natural transposon mobilization can drive adaptive genomic rearrangements. Here, we build on this capability and develop an inducible, self‐propagating transposon platform for continuous genome‐wide mutagenesis and the dynamic rewiring of gene networks in bacteria. We first use the platform to study the impact of transposon functionalization on the evolution of parallel *Escherichia coli* populations toward diverse carbon source utilization and antibiotic resistance phenotypes. We then develop a modular, combinatorial assembly pipeline for the functionalization of transposons with synthetic or endogenous gene regulatory elements (e.g., inducible promoters) as well as DNA barcodes. We compare parallel evolutions across alternating carbon sources and demonstrate the emergence of inducible, multigenic phenotypes and the ease with which barcoded transposons can be tracked longitudinally to identify the causative rewiring of gene networks. This work establishes a synthetic transposon platform that can be used to optimize strains for industrial and therapeutic applications, for example, by rewiring gene networks to improve growth on diverse feedstocks, as well as help address fundamental questions about the dynamic processes that have sculpted extant gene networks.

## Graphical abstract

A modular, transposon‐based platform is developed to dynamically rewire gene regulatory networks in bacteria. The continuous mobilization of transposons throughout the host genome yields multi‐site adaptive mutations and growth phenotypes in both static and dynamic selective environments.

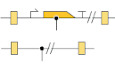

## Introduction

Mobile genetic elements (MGEs) have played a fundamental role in the evolution of complex cellular organisms and can be found across all kingdoms of life (Kazazian, 2004). Transposons are an ancient and diverse family of MGEs that have helped drive several major evolutionary transitions (Koonin, 2016) and have provided functional raw material for extant gene regulatory networks (GRNs; Feschotte, 2008; Aziz *et al*, 2010; Chuong *et al*, 2017; Cosby *et al*, 2021). Furthermore, the diverse molecular functions encoded in transposons and other MGEs present a rich source for the discovery of new tools for synthetic biology (Friedland *et al*, 2009; Roquet *et al*, 2016), genome engineering (Strecker *et al*, 2019; Vo *et al*, 2021), and gene editing (Altae‐Tran *et al*, 2021). Given the established role played by transposons in microbial genome dynamics (Vandecraen *et al*, 2017) and their impact on the adaptability and evolvability of gene regulatory networks (Pósfai *et al*, 2006; Umenhoffer *et al*, 2010; Vernyik *et al*, 2020), we reasoned that they would be well suited to form the basis of an engineered, self‐propagating platform for laboratory genome evolution (Zhou *et al*, 2021).

Current continuous directed evolution technologies focus on accelerating nucleotide‐level diversification within genome‐orthogonal replicators or defined genetic loci (Johnston *et al*, 2020; Morrison *et al*, 2020). By contrast, our limited ability to control and interpret larger structural changes in laboratory evolution experiments constrains both the phenotypes that can be explored and our fundamental understanding of the underlying evolutionary processes. A major challenge in directed genome evolution is our limited ability to dynamically track the mutations across a genome and mechanistically link these changes to the emergent phenotype (Luo *et al*, 2018). As a result, many of the processes generating genome‐wide structural heterogeneity in cells—for example, the duplication, translocation, transposition, or recombination of DNA segments (Kirchberger *et al*, 2020)—have yet to be adapted into engineering approaches for fitness landscape exploration during laboratory evolution (Ma *et al*, 2019; Gowland & Jewett, 2022).

The extensive suite of transposon‐based tools developed for high‐throughput loss‐of‐function and gain‐of‐function genetic screens (e.g., transposon insertion sequencing, Tn‐seq) present a genome‐scale solution to this problem (Cain *et al*, 2020). However, these screening pipelines involve a single, static round of mutagenesis and have not been optimized for continuous evolution. While they represent powerful methods for quantifying gene essentiality across environments (Price *et al*, 2018) and studying specific phenotypes of interest (Wang *et al*, 2011; Santiago *et al*, 2018), they do not allow for sequential, multisite mutagenesis. Nor do they reveal how evolutionary interactions between transposons and their hosts shape the structure of GRNs (Kleckner, 1990; Szitenberg *et al*, 2016). These dynamic perturbations could, however, accelerate strain engineering through gene network rewiring (Isalan *et al*, 2008; Windram *et al*, 2017) and build rich datasets for network‐level models of cell phenotypes (Lopatkin & Collins, 2020). In turn, this could provide a “proof‐by‐construction” platform to study the impact of analogous processes on the evolution of natural systems—a central tenet of synthetic biology (Bashor & Collins, 2018).

Here, we present an engineered, self‐propagating transposon platform to study the role of MGEs in the evolution of complex natural GRNs and harness their capacity to accelerate genome diversification through genome‐wide, *in vivo* mutagenesis. Existing transposon tools for genetic screens have been developed on an *ad hoc* basis and lack the ability to continuously rewire endogenous networks to probe their evolvability. Building on the hyperactive *mariner* transposase derivative *himar1C9* from the horn fly *Haematobia irritans* (Lampe *et al*, 1999), we adopt a synthetic biology framework to transposon platform design that focuses on three areas: (i) incorporating a systematized, modular assembly workflow to deliver functionalized transposon variants for gene network rewiring (Liu *et al*, 2018); (ii) comparing the impact of engineered transposon variants on host phenotypes in parallel evolving populations of an *Escherichia coli* MDS42 strain that lacks endogenous MGEs (Pósfai *et al*, 2006); and (iii) using unique DNA barcodes to track the locations of individual transposon lineages within host populations via longitudinal next‐generation sequencing (NGS) readouts (Wetmore *et al*, 2015). We demonstrate that this transposon platform can recapitulate examples of natural insertion‐mediated host adaptations to both nutrients and antibiotics. We then compare the impacts of transposon functionalization (e.g., with inducible outward‐facing promoters) on the evolution of *E. coli* populations toward diverse carbon source utilization in over 500 parallel cultures, observing two classes of gain‐of‐function mutation: promoter insertions upstream of catabolic enzymes, and loss‐of‐function insertions within their respective transcriptional repressors. Finally, as a proof‐of‐concept experiment with dynamic environments, we track parallel evolutions across alternating carbon sources and demonstrate the emergence of inducible, multigenic phenotypes through multilocus transposon insertions. This work expands our ability to develop optimized strains for biotechnological applications and creates a test bed for studying how co‐regulated gene networks evolve through the dispersal of mobile genetic elements.

## Results

### Constructing a self‐propagating transposon mutagenesis platform

**Figure 1:**
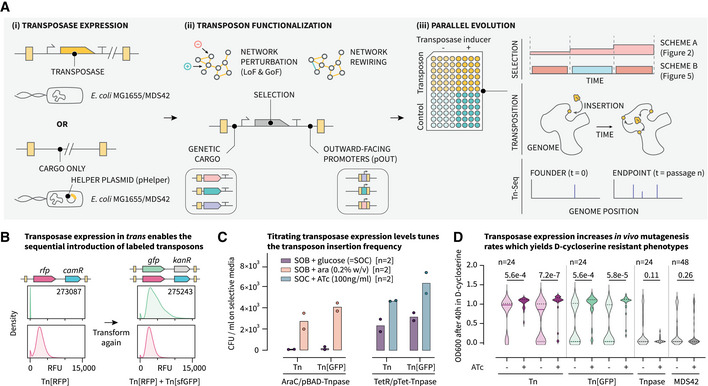
Engineered platform for continuous transposon‐mediated mutagenesis and gene regulatory network rewiring Our approach to genome evolution can be broken down into three core elements: (i) a mechanism to control transposase expression either from within the transposon (in *cis*) or from an independent pHelper plasmid (in *trans*); (ii) a DNA assembly platform to functionalize and barcode transposon variants, allowing *in situ* network rewiring and lineage tracking, respectively; and (iii) a parallelized approach to evolution focusing on comparing replicate lineages across different transposon variants and environmental conditions. These experiments can be coupled to a modified Tn‐Seq pipeline for genome‐wide insertion site identification.Transposase expression in *trans* enables the sequential insertion of transposons functionalized with orthogonal fluorescent reporter cassettes into the genome of *E. coli* MG1655 cells, as determined by flow cytometry (FITC, 488 nm; mCherry, 561 nm). The data represent ~2.7 × 10^5^ cells from pooled colonies of two sequential transformations.A comparison of two strategies to titrate transposase expression levels from the pHelper plasmid utilizing either pBAD or pTet. We used relative CFU counts on selective plates as a proxy for insertion frequencies following transformation of chemically competent MDS42‐pHelper cells with the transposon donor suicide vector (R6K origin of replication, *n* = 2 technical replicates). The inducers (arabinose or ATc) were added after the heat shock, during the rescue in SOC/SOB (1 h, 37°C).The introduction of transposase and transposon elements into MDS42 cells lacking endogenous MGEs increases the rate of *in vivo* mutagenesis. As a proxy, we measured the proportion of replicate, bottlenecked cultures that develop resistance to the antibiotic D‐cycloserine (20 μM) via the spontaneous inactivation of the *cycA* gene (e.g., through transposon insertion; Fehér *et al*, 2006; Pósfai *et al*, 2006; Umenhoffer *et al*, 2010). End‐point ODs (40 h, 37°C) were measured for *n* = 24 colonies each from duplicate transformations, *n* = 24 colonies for the parental MDS42‐pHelper strain, and *n* = 48 for “wild‐type” MDS42. The adjusted *P*‐values are shown above the violin plots and correspond to FDR‐corrected two‐sided Wilcoxon signed‐rank tests between populations cultured with or without ATc. Tn, mariner transposon (kanR); Tn[sfGFP], mariner transposon harboring a green fluorescent protein (*sfGFP*) gene; Tn[GFP], mariner transposon harboring an *mNeonGreen* gene; Tnpase, *himar1C9* transposase; ATc, anhydrotetracycline; ara, arabinose; Tn‐Seq, transposon insertion sequencing. Source data are available online for this figure.

Our platform for continuous, transposon‐mediated genome evolution can be broken down into three core functionalities: (i) an inducible mechanism for transposase expression in *cis* or in *trans* (Fig 1A(i)); (ii) a DNA assembly strategy to functionalize transposons with natural and synthetic cargos (e.g., reporters, promoters, and/or transcription factors) and thereby enable gene network rewiring (Fig 1A(ii); Isalan *et al*, 2008); and (iii) a high‐resolution, genome‐wide readout of insertion location based on NGS (Fig 1A(iii)). To build this self‐propagating transposon mutagenesis platform, we started with the well‐established *himar1C9* element from the *mariner* family of Class II DNA transposons. This transposon has been optimized for high insertion frequencies and is orthogonal to natural microbial MGEs (Lampe *et al*, 1996). Furthermore, it yields a random distribution of insertions at TA sites throughout the genome and is compatible with a range of NGS pipelines for insertion localization (Cain *et al*, 2020). To enable the continuous self‐propagation of the transposon within the genome, we explored two strategies for the expression of the transposase: the first mimics a natural transposon, with the transposase acting in *cis* from within the region flanked by the inverted repeat (IR) sequences (Fig 1A(i)), while the second uses a medium copy helper plasmid (pHelper) to provide transposase acting in *trans* (Fig 1A(i)).

In this study, we primarily focus on the plasmid‐based transposase expression strategy with the aim of prototyping and characterizing parallel transposon variants in a single common pHelper strain. To verify that sustained transposase expression in *trans* could support the sequential incorporation of differentially labeled transposons, we began from an *E. coli* MG1655 strain containing a pHelper plasmid encoding a transposase on a carbenicillin resistance (carb^R^) backbone. We transformed this strain with a donor plasmid (pDonor) harboring an RFP‐ and chloramphenicol resistance (cam^R^)‐labeled transposon on a suicide vector backbone and measured both GFP and RFP levels across a population inoculated from carb^R^/cam^R^ colonies. As expected, we only observed a population of RFP‐labeled transposons (Fig 1B, left column). Using competent cells made from this ensemble of insertion mutants, we sequentially transformed them with a GFP‐ and kanamycin resistance (kan^R^)‐labeled transposon on a suicide vector backbone. After selection for carb^R^/cam^R^/kan^R^ clones, we observed two populations of transposons (RFP‐ and GFP‐labeled) within the genomes of the same cells (Fig 1B, right column). This confirmed the capacity for the sequential genomic insertion of differentially labeled transposons using a transposase acting in *trans*.

To allow for external user control over the levels of transposase expression, we tested two pHelper designs: an arabinose‐inducible AraC/pBAD‐transposase expression vector (Armbruster *et al*, 2017), and an anhydrotetracycline (ATc)‐inducible TetR/pTet‐transposase (cam^R^) expression vector. First, we compared the number of colony‐forming units (CFUs) following the side‐by‐side transformation of an *E. coli* MDS42 pHelper strain with the two transposon donor variants on R6k suicide vector backbones. The “Tn” transposon variant contained a gene encoding kanamycin resistance, whereas the “Tn[GFP]” transposon variant contained an additional gene encoding an mNeonGreen fluorescent reporter as a representative synthetic cargo. As the donor plasmids harboring these transposons cannot propagate in MDS42 cells that lack the *pir* gene, the transformation frequency represents a simple proxy for the frequency of initial transposition events from the donor plasmid and their integration into the genome (Fig 1C). Active repression of the pBAD promoter in the presence of glucose resulted in a relatively low background insertion frequency. Conversely, while the TetR/pTet‐transposase (cam^R^) expression platform resulted in a higher absolute number of insertion mutants, the uninduced state also retained an intermediate level of activity. Notably, we did not observe a decrease in the number of insertion mutants with the Tn[GFP] variant (Fig 1C), suggesting that the transposons are still capable of efficient insertion when functionalized with synthetic cargo that is not essential for survival on antibiotic selection.

Next, we sought to measure the impact of this transposon‐transposase system on *in vivo* mutation rates following the initial insertion. We modified an established protocol that uses the spontaneous emergence of resistance to the antibiotic D‐cycloserine (D‐cyc)—typically following inactivation of the *cycA* gene—as a composite metric for the various sources of genomic mutation rates (Wargel *et al*, 1971; Fehér *et al*, 2006), including MGE‐mediated gene disruptions (Pósfai *et al*, 2006). In the context of our engineered platform, transposon insertions into the *cycA* gene, which encodes for the primary D‐cyc importer, would give rise to D‐cyc resistant (D‐cyc^R^) mutants (Baisa *et al*, 2013). Starting from multiple unique founder insertions grown under nonselective conditions, we measured the frequency of D‐cyc^R^ wells emerging in these replicate populations following a severe bottleneck (i.e., a 10,000‐fold dilution) and their subsequent transfer to media containing D‐cyc. In this continuous, *in vivo* mutagenesis experiment, we consistently observed a higher frequency of D‐cyc^R^ wells in replicates harboring both a transposon and transposase, which was further increased upon the addition of the transposase inducer ATc (Fig 1D). This trend was observed for both the Tn and Tn[GFP] transposon variants, further supporting the capability for adaptive insertion when the transposons are functionalized with synthetic cargo. For both transposon variants, we observed a broad distribution in end‐point optical density values (Fig 1D) which can result from secondary insertions into the host genome that impact growth, or from early‐arising mutants that have remained in stationary phase for an extended period of time. Together, these experiments indicate that while the AraC/pBAD‐transposase expression platform offers tighter control, the TetR/pTet‐transposase (cam^R^) expression platform provides a means to increase the rate of transposon‐mediated mutations *in vivo*. Importantly, ATc also meets our need for an inducer that is orthogonal to the nutrient content of the growth media used for subsequent laboratory evolution experiments. In agreement with previous studies, we observed a lower number of adaptation events in populations of the parental *E. coli* MDS42 strain lacking the engineered transposon and transposase (Fig 1D; Pósfai *et al*, 2006). In this and subsequent experiments, *E. coli* MDS42 represent an important negative control that accounts for the impact of other sources of genomic variation orthogonal to our engineered transposon platform.

These results demonstrate that by maintaining transposase expression in *trans*, orthogonal transposons can be continuously mobilized to yield adaptive insertions in the genomes of host cells under selection (Fig 1D). Building on this, we envisaged a general workflow for a self‐propagating transposon system for the dynamic rewiring of genomic networks. The platform we develop here uses established insertion sequencing pipelines to track transposon locations genome‐wide over the course of directed evolution experiments and could therefore be used as a screening approach to identify gene network rewirings that give rise to desired phenotypes. Moving forward, we set out to validate this platform using both static and dynamic selection conditions and compare the evolutionary impact of different transposon architectures (Dataset EV1) in high‐replicate, parallel growth selections.

### Benchmarking the platform against a natural transposon‐mediated adaptive switch

**Figure 2:**
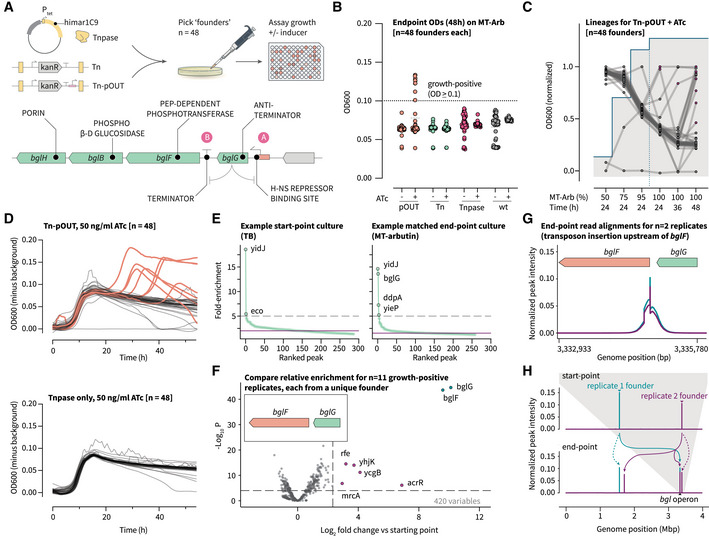
Validating the transposon‐dependent activation of a model, cryptic metabolic operon The adaptation of wild‐type *E. coli* isolates to growth on the carbon source arbutin involves the disruption of an H‐NS repressor binding site (site A) upstream of the positive regulator *bglG* by IS1 or IS5, and the subsequent activation of the structural genes *bglF/B/H* (Schentz, 1995; Hall, 1998). We introduced two transposon variants, Tn and Tn‐pOUT, into MDS42‐pHelper cells and cultured 384 parallel populations each derived from unique, single‐colony founders in media with an increasing proportion of arbutin. We observed an alternative insertion‐activation mechanism for Tn‐pOUT transposons at Site B within the operon.End‐point OD600 measurements (48 h, 1.0 g/l arbutin) for *n* = 48 replicates per condition.OD measurements normalized to minimum and maximum values for *n* = 48 replicates in a single condition (Tn‐pOUT + ATc, (B) orange points) showing the points at which high‐growth phenotypes emerge in 100% MT‐arbutin media (1.0 g/l). The blue line indicates the proportion of MT‐Arb in the growth media.Growth curves for two conditions (Tn‐pOUT and Tnpase only, both with ATc) in MT‐arbutin media (1.0 g/l), inoculated from the 24‐h time point in 95% MT‐arbutin media from (C). The curves from high‐growth replicates are highlighted in orange (*n* = 48 replicates each).Fold‐enrichment values for peaks identified using MACS3, based on aligned Tn‐Seq reads from the start‐point (TB, left) and end‐point (MT‐arbutin, right) cultures for a single founder colony. The initial insertion (*yidJ*) is the dominant peak before selection and is retained alongside multiple new, high‐intensity peaks postselection including *bglG*.By aggregating all high‐growth variants (*n* = 11) from the experiment in (B) and pairing start‐point and end‐point Tn‐Seq data, we used Bio‐Tradis (Barquist *et al*, 2016) to identify differentially enriched insertion loci (labeled points). Differential transposon enrichment analysis was performed using a negative binomial generalized linear model with Benjamini–Hochberg correction for multiple hypothesis testing (as implemented in the Bio‐Tradis toolkit).All (11/11) of these replicates exhibited a sharp insertion peak at a TA site upstream of *bglF*, suggesting the Tn‐pOUT mediated activation of the *bglF/B/H* operon. Two representative end‐point traces (teal and purple) are shown from the 11 replicates.Longitudinal sequencing enables the genome‐wide tracking of transposon movement. After selection, the transposons demonstrate both preservation of their initial insertion location (dashed arrows) and their convergence on the *bgl* operon (solid arrows). Additional intermediate insertions were also observed (solid, purple arrow near 1.7 Mbp) outside of the *bgl* operon. The top panel shows representative traces (teal and purple) corresponding to start‐point transposon insertions for two out of 11 replicates. The bottom panel shows end‐point insertions for the same, representative replicates. The insertion plots were generated by counting the number of NGS reads aligning to each position in the *E. coli* MDS42 genome and normalizing by the total number of reads per replicate. Tn, unmodified *mariner* transposon; Tn‐pOUT, *mariner* transposon with an outward‐facing pJ23104 promoter. Source data are available online for this figure.

MGEs can play an important role as genetic switches, remodeling the host genome in response to environmental changes in ways that can be adaptive for their hosts. One well‐documented example of this is the activation of the *bgl* operon to facilitate growth on the glycoside arbutin (Fig 2A): frequent transposition of the endogenous insertion sequence (IS) elements IS1 or IS5 into the operator of the regulatory gene *bglG* disrupts a binding site for the repressor H‐NS, leading to an increase in *bglG* expression and the upregulation of the structural genes *bglF*, *bglB*, and *bglH* (Schentz, 1995; Hall, 1998). Guided by previous work developing this phenomenon into an assay for MGE activity (Pósfai *et al*, 2006), we used it to benchmark the continuous *himar1C9* mutagenesis platform in *E. coli* MDS42 cells lacking IS1 and IS5. To increase the throughput of this assay, we switched from measuring CFU formation on solid arbutin media (requiring ~1 plate per initial founder) to using longitudinal optical density (OD) measurements in liquid media (1 well per initial founder). We first tested different combinations of host strain (*E. coli* MDS42 or MG1655), transposase induction systems [TetR/pTet (in *cis* and in *trans*) or AraC/pBAD (in *trans* only)], and arbutin selection schemes (Appendix Figs S1 and S2). These experiments confirmed previous observations that populations containing endogenous and/or artificial transposon systems adapt more frequently to growth on arbutin than those lacking MGEs (Umenhoffer *et al*, 2010). To confirm the mechanism for these phenotypes in the context of our artificial transposon platform, we then performed a final experiment with 48 unique founder insertions across four strains: the parental MDS42 strain, an MDS42 strain with pHelper only (transposase only), MDS42 pHelper cells with a genomic transposon (Tn), and a final variant in which the transposon harbors an outward‐facing, constitutive pJ23104 promoter (Tn‐pOUT). By including transposon variants with and without an outward‐facing promoter (Fig 2A), we were able to investigate evolutionary trajectories that rely on loss‐of‐function versus gain‐of‐function mutations, respectively. Furthermore, each group of 48 replicates was cultured under two inducer conditions (0 or 50 ng/ml ATc) to enforce either intermediate or high levels of transposase expression, for a total of 384 parallel evolving populations (Fig 2B).

To assess the adaptation frequencies for each group, we passaged populations in 96‐well plate format through minimal media with an increasing ratio of arbutin to glucose as a carbon source. Importantly, in our general workflow the initial replicate cultures were founded from individual colonies which must have undergone at least a single insertion event with the transposon harboring the antibiotic resistance cassette in order to propagate during the initial antibiotic selection. Within the timescale of this experiment, high‐OD end‐point phenotypes (11/48 replicates) were only observed in the Tn‐pOUT strain exposed to the ATc inducer (Fig 2B), with the growth‐positive phenotypes emerging at both early and late time points (Fig 2C and D). Based on this evidence for a transposon variant‐specific growth phenotype (Fig 2D), we modified an established Tn‐Seq pipeline to compare the start‐ and end‐point insertion locations across all 11 replicates (Fig 2E and Appendix Fig S3). As expected, for the preselection populations each of the 11 replicates was identified with a unique, highly enriched “founder” insertion (except in one case, where the initial insertion occurred near a multicopy ribosomal gene; Appendix Fig S4). In every corresponding end‐point population that evolved a high‐growth phenotype on arbutin, a second highly enriched peak was identified in the terminator region downstream of *bglG* and upstream of *bglF* (Fig 2F and G). While the transposon orientation cannot be determined by the sequencing data alone, the transposon is likely oriented such that the constitutive promoter is driving the expression of *bglF*/*B*/*H*, which are essential for arbutin catabolism. Moreover, we consistently observed the preservation of the donor site during transposon movement (Fig 2H), in keeping with previous reports on transposon remobilization (Hagemann & Craig, 1993; Vo *et al*, 2021).

This experiment demonstrates that evolution from multiple distinct founder insertions of an engineered Tn‐pOUT converges on a qualitatively equivalent growth outcome to natural IS1/IS5 insertions (Reynolds *et al*, 1981; Schnetz & Rak, 1992; Amster‐Choder, 2005), but via a distinct mechanism—namely, the direct upregulation of *bglF/B/H* expression rather than via the disruption of H‐NS‐mediated *bglG* repression (Schentz, 1995; Hall, 1998). In our evolution scheme, the direct upregulation of *bglF/B/H* expression is likely a favorable adaptation because these genes do not contain internal transcriptional repressor binding sites. In contrast, *bglG* contains an additional intragenic H‐NS binding site which would likely need to be outcompeted by the outward‐facing promoter that is harbored on the transposon (Dole *et al*, 2004). Hence, adaptation solely through the disruption of H‐NS‐mediated *bglG* repression would likely result in slower accumulation of *bglF*/*B*/*H* and increase the amount of time required for growth. Interestingly, in several of the replicate populations, our NGS‐based tracking approach allowed us to detect additional highly enriched peaks. Given the switch‐like adaptation to arbutin utilization and the fact that these additional peaks were generally not conserved between replicates, we hypothesize that they represent intermediate insertion events preceding the high‐fitness insertion at *bglF* (Fig 2H and Appendix Fig S4). Through genetic linkage, these intermediate insertions could be carried to fixation in the population. Taken together, these findings confirm the capacity of our engineered, orthogonal transposon system to replicate natural adaptive processes and highlight the important influence that specific functional differences (i.e., the presence of an outward‐facing promoter) can have on the overall contribution of MGEs to the evolvability of their host populations.

### Screening for transposon‐mediated metabolic adaptations

**Figure 3:**
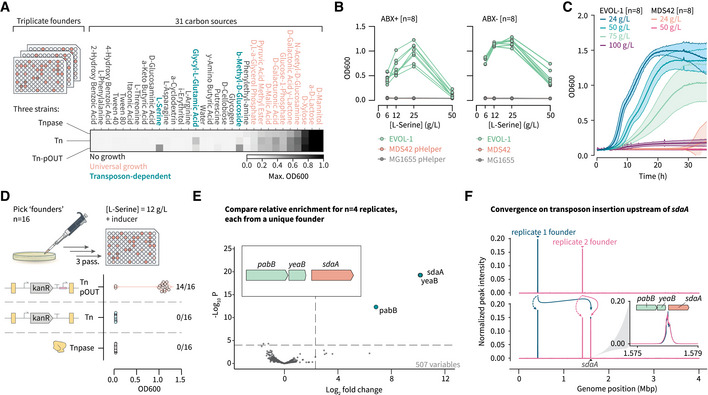
Screening for transposon‐dependent carbon source utilization phenotypes In a preliminary screen of 31 carbon sources, we assessed the adaptation frequencies of triplicate founder colonies for two transposon variants (Tn and Tn‐pOUT with pJ23104). As a negative control, we included three colonies from the MDS42 pHelper parental strain. The heatmap shows the maximum OD across the three replicates for each condition, with the color map scaled to a maximum OD cutoff of 1.0. We identified three distinct groups: no growth (18 conditions), universal growth (10 conditions), and transposon‐dependent growth (3 conditions).L‐serine utilization was observed in 1/3 of the Tn‐pOUT replicates (EVOL‐1). Further comparison of this isolate to MG1655 and MDS42 demonstrated its high tolerance to L‐serine levels, both with (left panel) and without (right panel) antibiotic selection for the transposon and transposase (*n* = 8 replicate colonies each).Detectable growth at even higher concentrations of L‐serine (up to 100 g/l) was observed in the presence of the preferred carbon source glucose (4.0 g/l). The data are from the same eight colonies as (B) grown under different conditions.A repeated screen focusing on L‐serine increased the number of unique founder replicates to 16. Reproducible evolution of L‐serine tolerance was only observed with the Tn‐pOUT transposon (14/16 replicates). OD measurements were taken after 50 h of growth on the third passage in M9 L‐serine media.Using Tn‐Seq data from four of these samples at both the start‐ and end‐points, we identified three genes from a single region showing high differential enrichment: *pabB*, *yeaB*, and *sdaA*. Differential transposon enrichment analysis was performed using a negative binomial generalized linear model with Benjamini–Hochberg correction for multiple hypothesis testing (as implemented in the Bio‐Tradis toolkit).Genome‐wide transposon insertion maps showing convergence from distinct founder peaks (top panel) on a single conserved location, postselection (bottom panel). High‐resolution alignments from two representative samples confirm that insertions near both *pabB* and *yeaB* are in the promoter region upstream of *sdaA* (L‐serine deaminase I, inset). Source data are available online for this figure.

To test the power of our genome evolution platform to identify insertion‐dependent growth phenotypes and generate strains capable of diverse feedstock utilization, we designed a rapid screen of 31 carbon sources in a standard plate‐based assay (Garland & Mills, 1991). As before, we incorporated two transposon variants (Tn and Tn‐pOUT) into the screen in parallel, allowing us to directly compare their impacts on host adaptability and to distinguish evolutionary trajectories relying on loss‐ or gain‐of‐function mutations. We initiated the screen with three founder colonies for each transposon variant and included three colonies from the parental MDS42 pHelper strain as negative growth controls. The strains were initially inoculated into nonselective minimal media containing glucose, and a small population from these cultures (i.e., approximately 1,000 cells) were then passaged into minimal media containing a new carbon source. The cells were then serially passaged every 32 h into fresh media containing the same carbon source as the previous passage. After three passages in media containing the new carbon source, this screen identified three carbon sources in which only the transposon‐harboring strains appeared to exhibit a partial or complete growth advantage arising during serial passaging: L‐serine, glycyl‐L‐glutamic acid, and β‐methyl‐D‐glucoside (Fig 3A and Appendix Fig S5). Both glycyl‐L‐glutamic acid (Durso *et al*, 2004) and β‐methyl‐D‐glucoside (Schaefler & Malamy, 1969; AbuOun *et al*, 2009) have previously been described as examples of growth conditions that differentiate laboratory strains and environmental strains of *E. coli*. At high concentrations, L‐serine is toxic and inhibits cell growth (Zhang *et al*, 2010). In our preliminary screen, one replicate of the Tn‐pOUT lineages showed detectable growth on L‐serine as a sole carbon source (EVOL‐1), suggesting a possible gain‐of‐function mechanism for this adaptation. Further characterization of this isolate showed its ability to grow in L‐serine concentrations up to 50 g/l, while unevolved MDS42 and MG1655 showed no detectable growth across this range (Fig 3B). We used previous efforts to evolve strains capable of high‐yield L‐serine production as a benchmark for the fitness of EVOL‐1 in the presence of a more favorable carbon source (i.e., glucose; Mundhada *et al*, 2017) and observed extreme tolerance up to 100 g/l of L‐serine (Fig 3C).

To determine how reproducibly this phenotype evolved and to identify the underlying genetic changes, we augmented the initial screen with a second experiment comparing 16 unique founder colonies across the same three strains—passaging the plates three times in 12 g/l of L‐serine as the sole carbon source in the presence of ATc. We observed growth in 14/16 colonies when the transposon was functionalized with a constitutive outward promoter, but no growth in cultures with either an unfunctionalized promoter or no transposon (Fig 3D). Using Tn‐Seq data that we collected pre and postselection for four colonies, we identified a reproducible insertion upstream of the *sdaA* gene (Fig 3E and F, and Appendix Figs S6 and S7). SdaA (L‐serine deaminase I) is an iron–sulfur cluster‐containing enzyme that catalyzes the conversion of L‐serine to pyruvate and ammonium (Su & Lang, 1989; Tuan *et al*, 1990; Lin *et al*, 1992; Cicchillo *et al*, 2004). In *E. coli*, *sdaA* is typically regulated by a σ^32^‐specific promoter as part of the stress response, and its activity *in vitro* is impaired under aerobic conditions due to the oxidative inactivation of the iron–sulfur complex (Newman *et al*, 1985). Transposon‐mediated adaptation to L‐serine utilization therefore proceeds via *sdaA* overexpression, representing a rewiring from the natural σ^32^‐mediated expression to the synthetic, constitutive σ^70^‐specific promoter harbored on the transposon. As with the arbutin evolution experiment (Fig 2), the use of a transposon functionalized with a constitutive promoter resulted in an adaptive mutation distinct from the natural mechanism—namely, the direct activation of *sdaA* as opposed to mutations in its transcriptional regulators that indirectly result in increased *sdaA* expression (Dataset EV2) (Ambartsoumian *et al*, 1994; Monette, 2006). We observed the same transposon insertion upstream of *sdaA* in cells containing a helper plasmid harboring a different antibiotic selection marker (Appendix Figs S6 and S7). These experiments highlight the reproducible and rapid generation of useful mutant phenotypes that can be achieved with this platform and demonstrate the ease with which the transposons can be tracked to identify the causative genetic changes (Fig 3E and F).

### A modular assembly pipeline facilitates rapid prototyping and barcode‐based tracking

**Figure 4:**
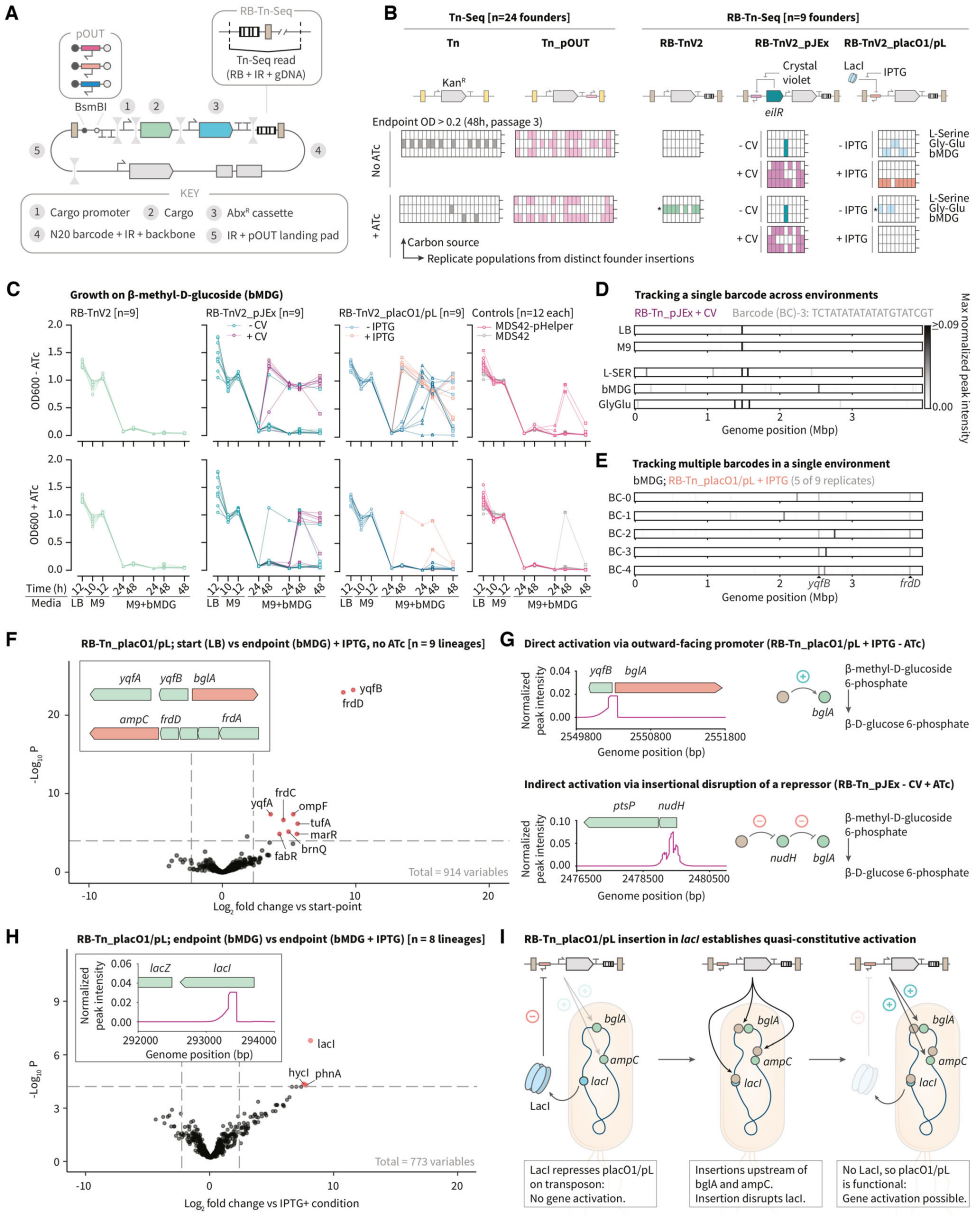
Modular assembly platform for transposon functionalization and barcode‐based lineage tracking A modified two‐layer Golden Gate assembly pipeline focusing on modular plasmid assembly (via BbsI) and promoter library insertion (via BsmBI) within the transposon enables rapid prototyping and transposon barcoding.Comparison of the evolutionary impacts of the two original transposons (Tn, gray; and Tn‐pOUT, pink; *n* = 24 per condition) with three second‐generation transposons (RB‐TnV2, green; RB‐TnV2_pJEx, teal/purple; RB‐TnV2_pLacO1/pL, blue/orange; *n* = 9 per condition), across the three carbon sources identified in Fig 3A. Each box represents a replicate culture, with each column derived from the same initial founder colony and each row corresponding to a unique carbon source. Colored cells indicate growth (end‐point OD600 > 0.2). Rows marked with a * symbol indicate contamination from RB‐TnV2_pJEx cultures as determined based on the unique sequencing barcode of each founder.OD600 measurements for each carbon source were used to track the emergence of growth phenotypes in cultures without ATc (top row) or with ATc (bottom row), and the transposon‐specific inducers CV or IPTG. We included two controls: MDS42 pHelper expressing transposase only, and the parental MDS42 strain (*n* = 12 per condition).Computational barcode demultiplexing from pooled sequencing runs for each unique variant‐environment combination enabled the reconstruction of insertion mutant lineages. For a single lineage, condition‐specific insertion spectra evolve from the initial founder insertion(s).By comparing independent lineages for a single carbon source‐inducer combination, convergent insertion loci emerge (black arrows).Differential enrichment of RB‐Tn‐Seq reads between paired start‐point and end‐point samples for independent lineages confirms the reproducible insertion sites. For MDS42 cells harboring transposons with a pLacO1 promoter in bMDG + IPTG, the two common peaks in (E) correspond to *frdD* and *yqfB* (likely activating *ampC* and *bglA*, respectively, inset).Representative read alignment traces from individual barcoded replicates showing two possible mechanisms of *bglA* activation under selection for growth on bMGD: upstream insertion of an RB‐TnV2_pLacO1/pL transposon with an outward‐facing pLacO1/pL promoter driving IPTG‐induced expression (upper panel), or direct insertion of an RB‐TnV2_pJEx transposon within a repressor of *bglA* (Deana *et al*, 2008) that likely disrupts protein function (lower panel). The accumulation of read alignments in the upper panel indicates that the transposon is oriented such that the outward‐facing promoter is driving expression of *bglA*.For RB‐TnV2_pLacO1/pL, growth phenotypes in bMDG emerge later in the absence of IPTG (C). Differential enrichment between paired IPTG^+^ and IPTG^−^ replicates implicates insertions in *lacI*, likely rescuing the activation potential of the pLacO1/pL transposons (inset: read alignments in the *lac* operon for a single barcoded replicate population). Differential transposon enrichment analysis was performed using a negative binomial generalized linear model with Benjamini–Hochberg correction for multiple hypothesis testing (as implemented in the Bio‐Tradis toolkit).A possible model for the establishment of quasi‐constitutive, RB‐TnV2_pLacO1/pL transposon‐mediated gene activation via the disruption of the endogenous repressor *lacI*. Insertions upstream of *bglA* and *ampC* then recapitulate the adaptive insertions seen in the bMDG + IPTG condition (F). RB, random barcode; IR, inverted repeat; CV, crystal violet; IPTG, Isopropyl ß‐D‐1‐thiogalactopyranoside; bMDG, ß‐methyl‐D‐glucoside. Source data are available online for this figure.

The results presented in this work so far, as well as those from diverse insertion mutagenesis screens (Cain *et al*, 2020), demonstrate that transposon functionalization can impact the outcome of a genome‐wide laboratory evolution experiment. To facilitate the rapid prototyping of different transposon variants and to support the option for pooled screens, we implemented a modular, combinatorial assembly pipeline that uses customizable collections of defined parts (Santos‐Moreno & Schaerli, 2019). Specifically, we modified an existing Golden Gate transposon assembly workflow (known as “Magic Pools”) to allow for the incorporation of libraries of outward‐facing promoter variants and additional genetic cargos (Fig 4A; Liu *et al*, 2018). Our assembly pipeline also supports the incorporation of DNA barcodes into the individual transposon molecules such that their unique molecular identity can be cross‐referenced with their genomic location via random barcode Tn‐Seq (RB‐Tn‐Seq; Fig 4A inset, and Datasets EV1 and EV3; Wetmore *et al*, 2015). To demonstrate the utility of this modular platform for the functionalization of transposons with synthetic or endogenous gene regulatory elements, we constructed three barcoded transposon variants: (i) RB‐TnV2, which lacks an outward‐facing promoter; (ii) RB‐TnV2_pJEx, which is based on the Jungle Express system and harbors an inducible promoter regulated by the engineered crystal violet (CV)‐responsive repressor EilR (Ruegg *et al*, 2018); and (iii) RB‐TnV2_placO1/pL, which harbors an IPTG‐inducible placO1/pL promoter regulated by the endogenous LacI protein (Lutz & Bujard, 1997). These inducible promoters were selected based on the orthogonality of their inducers to the nutrient content of the growth media. We confirmed their ability to activate gene expression across the transposons' inverted repeat sequence in a GFP‐based plasmid reporter assay (Appendix Fig S8). As a modification to the “Magic Pools” platform, we included the option to add cargo and a cargo‐specific promoter to the transposon in addition to the antibiotic selection cassette and the outward‐facing promoter system (and any associated regulators). While this would allow the addition of fluorescent reporters, biosensors, or promoter‐transcription factor pairs (Isalan *et al*, 2008), in the specific designs tested here we instead used a short, noncoding “spacer” part spanning both modules (Datasets EV1 and EV3).

In the context of our continuous *in vivo* mutagenesis platform, this modular transposon functionalization and barcoding approach has the potential to facilitate the characterization of highly parallelized evolutionary trajectories. Specifically, transposon barcoding allows for the longitudinal tracking of individual transposon lineages as they propagate within the genomes of evolving host populations. At each time point, pooling parallel replicates before sequencing increases the throughput (i.e., the number of founder colonies and/or time points that can be measured) and reduces the associated cost of each experiment. The independent, barcode‐defined transposon lineages can then be demultiplexed *in silico* and traced back from the end‐point to their initial founder insertion identified through sequencing at *t* = 0. To validate this framework, we initiated a large‐scale, parallel evolution assay using the three new barcoded transposon variants assembled using the “Magic Pools” workflow (RB‐TnV2, RB‐TnV2_pJEx, and RB‐TnV2_placO1/pL). With the addition of the two original transposon variants used in this work (Tn and Tn‐pOUT), as well as two control strains, we systematically compared their impact on *E. coli* MDS42 genome evolution under selection for growth on three different carbon sources (L‐serine, glycyl‐L‐glutamic acid, and β‐methyl‐D‐glucoside) with or without ATc (Fig 4B and Appendix Fig S9). For each transposon variant, we studied replicate populations initiated from distinct founder insertions (*n* = 24 or *n* = 9), and in the case of both RB‐TnV2_pJEx and RB‐TnV2_placO1/pL, we compared parallel lineages with or without their respective inducers (CV or IPTG).

Interestingly, the transposase inducer (i.e., ATc) and the inducers for the outward‐facing, inducible promoters (i.e., CV or IPTG) had different impacts on growth for each carbon source and specific barcoded transposon variant. For example, we noticed a reliance on CV for growth across all three carbon sources in populations harboring the RB‐TnV2_pJEx transposon variant, regardless of the presence or absence of ATc (Fig 4B; teal versus purple). In contrast, the populations harboring the RB‐TnV2_placO1/pL transposon variants only grew in the absence of ATc, while the impact of IPTG appeared to be carbon source dependent (Fig 4B; blue versus orange). In future experiments, an even larger number of replicate populations could help elucidate the reproducibility of these inducer‐carbon source relationships. In this experiment, different transposon architectures, inducer conditions, and controls were typically grown in the same 96‐well plates. All OD measurements were conducted at the same time points across wells and plates, and these plates were subsequently passaged regardless of the observed OD measurement for individual wells (Fig 4C and Appendix Figs S10–S13). Therefore, early‐arising growth phenotypes remain in stationary phase for longer, which can lead to a decrease in OD and hence a lower inoculum for subsequent passaging compared with wells with late‐arising growth phenotypes (Fig 4C).

To track the location of the transposons within the genomes of these evolving cells, we performed RB‐Tn‐Seq on pooled samples at three time points (Appendix Fig S14): after the first passage in LB (12 h), after a second passage in M9‐Glucose (12 h), and at the end of the third passage in the selective carbon sources (48 h). In these experiments, the transposon orientation is discernible based on the orientation of RB‐Tn‐Seq read alignments, which accumulate in the direction opposite to the outward‐facing promoter. For the populations harboring the barcoded transposon variants (i.e., RB‐TnV2, RB‐TnV2_pJEx, and RB‐TnV2_placO1/pL), the number of founding colonies is much lower than the theoretical number of unique 20 bp barcodes introduced through cloning. This ensures that the likelihood of the same 20 bp barcode sequence appearing in different founder colonies is low. Importantly, the unique barcode defining each founder colony supports both the computational demultiplexing of the underlying lineages of each pooled sample (Fig 4D and Appendix Fig S15), and the identification of rare cross‐contamination events between groups of pooled samples—a known problem in long‐term plate‐based assays, particularly if regular liquid transfer steps are required (DeBenedictis *et al*, 2022). This is routinely identified as a discrepancy in the barcode sequence for a unique replicate population when comparing a given end‐point strain to that of its respective founder. For example, we observed that two sets of end‐point samples that were evolved on glycyl‐L‐glutamic acid did not contain the same barcode sequences as their respective founder colonies. Instead, they contained barcodes that we could associate with a different lineage harboring an entirely different promoter architecture and therefore represent a false‐positive result (Appendix Fig S13). We were then able to discard these cross‐contaminated cultures from downstream lineage reconstructions, and any conclusions derived from them (Extended Dataset EV2 and Appendix Fig S3; Halpern & Umbarger, 1961; Vender *et al*, 1965; Williams *et al*, 1994; Hansen *et al*, 2005; Reitzer, 2005).

For each unique combination of carbon source and inducer(s) culminating in more than one independent growth phenotype, we compared demultiplexed, replicate lineages (Appendix Figs S16–S19) to identify reproducible insertion sites associated with carbon source utilization (Fig 4E, and Appendix Figs S20 and S21). We provide a detailed summary of the primary insertion mutations driving the growth phenotypes in Dataset EV2. Interestingly, with this transposon mutagenesis platform we could identify two broad types of perturbation: gain‐of‐function insertions upstream of catabolic enzymes required for carbon source utilization, and loss‐of‐function insertions within transcriptional regulators that typically act to repress these enzymes. As an example, under selection for growth on β‐methyl‐D‐glucoside, we repeatedly observed either the direct activation of *bglA* which encodes the enzyme 6‐phospho‐β‐glucosidase A (Fig 4F and G; Schaefler & Malamy, 1969; Prasad *et al*, 1973; Zangoui *et al*, 2015), or the inactivation of *nudH* (*rppH*) (Aguilar *et al*, 2012) which has been shown to increase *bglA* transcript levels by ~2.5× alongside hundreds of other genes (Fig 4G and Appendix Fig S20; Deana *et al*, 2008). In the former case, direct *bglA* activation reproducibly co‐occurs with insertions in the 3′ end of *frdD* (Fig 4F), where the ORF overlaps with the promoter of the downstream β‐lactamase gene *ampC* (Tseng *et al*, 1994). This lends evidence to previous work implicating *ampC* in the regulation of cell morphology in poor growth conditions and starvation (Henderson *et al*, 1997; Händel *et al*, 2013), possibly via its alternative function as a peptidoglycan hydrolase (Bishop & Weiner, 1993; Santos *et al*, 2002).

As a proof‐of‐concept demonstration for the ability of this transposon‐mediated genome evolution platform to introduce more complex, distributed perturbations to the host gene regulatory network, we looked for instances of multisite insertion. The OD traces for the IPTG^−^ and IPTG^+^ replicates of the TnV2_placO1/pL evolution (Fig 4C, third column) show a delayed but reproducible emergence of growth phenotype even in the absence of IPTG. Given the requirement for the upregulation of *bglA* (Fig 4F), we hypothesized that transposon insertion into the endogenous *lacI* gene would relieve repression on the outward‐facing placO1/pL promoter and establish quasi‐constitutive gene activation at other insertion sites. As evidence for this multisite insertion mechanism, we identified a common, additional *lacI* insertion in the IPTG^‐^ context by comparing the differential enrichment of reads across the *n* = 8 paired IPTG^−^ and IPTG^+^ conditions (Fig 4H). In short, the insertion of a transposon with an outward‐facing placO1/pL promoter upstream of *bglA* introduces an artificial regulatory interaction with *lacI* that can be disrupted through a transposon‐mediated knock‐out of *lacI* (Fig 4I).

### A continuous transposon mobilization platform facilitates evolution in dynamic environments

**Figure 5:**
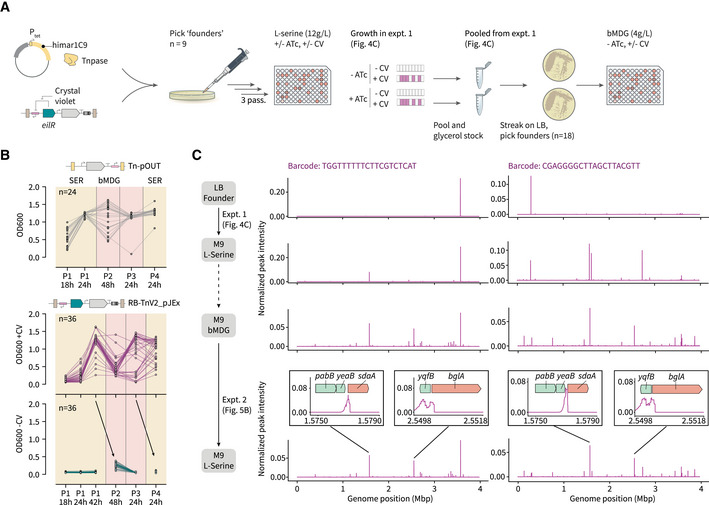
Dynamic environments with sequential carbon source selections drive the emergence of multisite insertions A schematic of the overall experiment, taking pre‐evolved RB‐TnV2_pJEx strains from the L‐serine + CV culture experiments in Fig 4C (*n* = 36 total) and reselecting on L‐serine, before transitioning to a new carbon source: ß‐methyl‐D‐glucoside. We performed a similar experiment in parallel with Tn‐pOUT strains pre‐evolved on L‐serine (*n* = 24).OD measurements for parallel populations of Tn‐pOUT (gray, *n* = 24) and RB‐TnV2_pJEx (purple, with CV, *n* = 36) derived from cultures pre‐evolved on L‐serine (Appendix Figs S9 and S10), and subsequently evolved toward growth on bMDG. In the absence of crystal violet, no growth was observed in the RB‐TnV2_pJEx cultures (teal, *n* = 36). Gaps indicate reseeding from the prior time point of the crystal violet‐induced cultures (arrows).Cross‐experiment lineages for two representative barcodes, tracking the distribution of transposon insertions after selection on L‐serine (Fig 4B and Appendix Fig S12) and after further selection on ß‐methyl‐D‐glucoside (B). Insets represent high‐resolution RB‐Tn‐Seq read alignments for the major peaks. RB, random barcode; IR, inverted repeat; CV, crystal violet; IPTG, Isopropyl ß‐D‐1‐thiogalactopyranoside; bMDG, ß‐methyl‐D‐glucoside. Source data are available online for this figure.

In contrast to static transposon mutagenesis experiments, a continuous platform for transposon mobilization and propagation could enable studies on the role of additive mutations in evolution experiments with fluctuating selection conditions. To test this possibility, we explored the impact of dynamic environments consisting of sequential carbon source selections on the number of highly enriched insertion mutations (Fig 5A). When cells derived from glycerol stocks of the variants preselected on L‐serine were recultured and switched to a new carbon source (β‐methyl‐D‐glucoside, Fig 5B), we saw an initial lag in OD600 before the eventual adaptation to this new carbon source in the presence of the inducer (CV) for the outward‐facing promoter. Importantly, parallel cultures lacking the inducer CV were unable to grow on L‐serine, or adapt to growth on β‐methyl‐D‐glucoside even after reseeding from high‐OD cultures containing CV (Fig 5B, bottom panel). This is likely due to the fact that the adaptive mutations observed in cultures growing on L‐serine and β‐methyl‐D‐glucoside require activation of *sdaA* and *bglA*, respectively (Fig 4). Longitudinal sequencing of these cultures identified the secondary emergence of highly enriched transposon insertions upstream of *bglA*, in the context of genomes already containing insertions upstream of *sdaA* (Fig 5C and Appendix Fig S22). In short, multisite insertions emerged supporting growth on both carbon sources through additive gain‐of‐function mutations, in a manner dependent on the supply of the inducer CV. Together with the results presented in Fig 4, these examples highlight two important advantages of our transposon‐mediated evolution platform for future studies: first, the possibility for multiloci mutagenesis enables the investigation of epistatic genetic interactions (Wong *et al*, 2016), and second, our ability to incorporate synthetic, orthogonal regulatory sequences (e.g., the pJEx promoter) into the transposons establishes a mechanism to introduce external, inducible control over these cellular phenotypes (Isalan *et al*, 2008).

## Discussion

In this work, we developed an engineered, self‐propagating transposon platform for continuous genome‐wide mutagenesis and dynamic GRN rewiring. To validate its generalizability and robustness, we used the platform to study the impact of transposon functionalization on the evolution of parallel *E. coli* populations toward diverse carbon source utilization and antibiotic resistance phenotypes (Dataset EV2). Through the implementation of barcode‐based tracking and longitudinal NGS, we were able to reconstruct transposon lineages within the genomes of pooled host cell populations and thereby increase the throughput and temporal resolution of the experiments. Enabled by this increase in throughput, future efforts could explore longer timescales and use a greater number of replicates to support a more statistical approach to measurements of evolvability (Chory *et al*, 2021; DeBenedictis *et al*, 2022), as well as facilitate the discovery of rare genetic innovations. We note three technical features of our platform that could be the focus of future work: namely, reductions in the leakiness of the TetR/pTet‐Tnpase system used to control transposase expression, an assessment of its functionality in bacterial hosts with high numbers of endogenous mobile genetic elements, and a characterization of the behavior of the transposon system in month‐ or year‐long passaging experiments. In the former case, a major obstacle is the limited availability of low‐leakiness promoter systems with inducers that are sufficiently orthogonal to avoid confounding the selection conditions (e.g., nutrient conditions).

Our final proof‐of‐concept demonstration advanced an underexplored aspect of laboratory evolution experiments (Fig 5): namely, the contribution of more complex, dynamic environments to the emergent structures of gene regulatory and metabolic networks (Szappanos *et al*, 2016). In this context, the advantage of our transposon platform lies in its apparent ability to introduce insertions at multiple locations in a genome through changes in the copy number of the transposon, mimicking the models for the creation of co‐regulated gene networks through MGE dispersal typically associated with eukaryotic genomes (Feschotte, 2008). The continued development of microbial single‐cell genomics technologies will improve our ability to determine whether these insertion events are in the same genome, or in different positions within the genomes of two or more divergent but co‐resident host lineages. Technological solutions to the intragenomic mapping of multi‐insertion mutants could stem from modifications to the recently described droplet Tn‐Seq (dTn‐Seq) approach (Thibault *et al*, 2019), but true single‐cell bacterial sequencing remains challenging (Woyke *et al*, 2017; Kuchina *et al*, 2021). Alternatively, the differential fluorescent labeling of intracellular transposon subpopulations could be coupled to cytometry readouts to measure relative changes in their copy number (Fig 1B). These approaches could then be used to parameterize mathematical models of intragenomic competition between transposons and study the emergence of superparasitism between co‐resident, autonomous transposons (Startek *et al*, 2013).

We anticipate our genome evolution platform playing an important role in screening efforts to improve strains for industrial and therapeutic applications. As an example, fitness‐conferring mutations that improve growth on diverse feedstocks (Mundhada *et al*, 2017; Gowland & Jewett, 2022) could be identified through the transposon‐mediated rewiring of GRNs. Similar mutations could then be rationally introduced without the transposon components to establish a final, stable strain. In this context, our platform exhibits several features that facilitate its extensibility to diverse bacterial strains and applications. First, the *himar1C9* mariner transposase that is used in our platform is compatible with a broad range of bacterial hosts (Goodman *et al*, 2009; Perry & Yost, 2014; Stocks *et al*, 2022). Second, the modular assembly pipeline that is used to construct the donor plasmids enables the transposons to be functionalized with diverse genetic cargos and accelerates prototyping for diverse microbial hosts (Liu *et al*, 2018). Third, our platform is compatible with both small and large starting library sizes: As an example of the former, the evolution experiments in this study were initiated from individual founder colonies. They represent a viable genome‐wide genetic screening approach for strains that have low transformation efficiencies and therefore preclude standard saturation mutagenesis. In this framework, genetic diversity can be generated from a small set of founder colonies (10^0^–10^2^) through the continuous mobilization of the transposon to new sites, thereby creating a more diverse library. Conversely, the use of barcode‐associated transposons allows the pooling of many founder strains to substantially increase the number of evolutionary trajectories that can be characterized as an ensemble.

We envision these features facilitating genome engineering efforts in species commonly used for bioproduction and as engineered live biotherapeutics. Many bacteria that are used as engineered live biotherapeutics are unable to engraft in the mammalian gut (Isabella *et al*, 2018; Russell *et al*, 2022) and require repeated administration for treating chronic diseases. In future studies, our platform could be used to deliver transposons that are functionalized with either random or rationally selected (e.g., metagenomically derived (Crook *et al*, 2019)) gene libraries to identify loss‐ and gain‐of‐function mutations that promote engraftment in microbially complex gut environments. Furthermore, the transposon‐mediated delivery of catabolic or biosynthetic genes could help optimize bioproduction strains by dynamically exploring how growth or product yield is impacted by gene copy number, genomic integration site, and the inactivation of nonessential genes. This platform could also serve as a test bed for longer‐term evolution experiments designed to study the dynamics of host‐MGE or MGE‐MGE interactions and, in particular, how these evolutionary processes are impacted by contingency (Szappanos *et al*, 2016) and environmental complexity (Startek *et al*, 2013; O'Brien *et al*, 2016; Sandberg *et al*, 2017). Transposons functionalized with well‐defined, endogenous regulatory elements—such as heat‐shock or nutrient‐specific promoters—could be used to test current models for the relationship between environmental predictability and the evolution of anticipatory genetic regulation under oscillatory selection regimens (Mitchell *et al*, 2009; Freddolino & Tavazoie, 2012). Ultimately, we expect this artificial evolution platform to help answer fundamental questions about the dynamic processes that have sculpted extant GRNs.

## Materials and Methods

### Reagents and Tools table

**Table:** 

Reagent/Resource	Reference or Source	Identifier or Catalog Number
**Experimental models**		
*E. coli*: *MDS42*	Pósfai *et al* (2006)/Scarab Genomics	C‐6265
*E. coli*: MG1655	ATCC	700926
*E. coli*: OneShot PIR2 competent cells	Invitrogen	C111110
*E. coli*: Turbo competent cells	New England Biolabs	C2984H
**Recombinant DNA**		
pHdCas9	Addgene	#137080
pSAM_AraC	Addgene	#91569
pSAM_Ec	Addgene	#102939
pDonor_Tn	This study	N/A
pDonor_Tn[sfGFP]	This study	N/A
pDonor_Tn[GFP]	This study	N/A
pDonor_Tn[RFP]	This study	N/A
pDonor_Tn‐pOUT	This study	N/A
pDonor_Tn[himar1C9]	This study	N/A
pDonor_RB‐TnV2	This study	N/A
pDonor_RB‐TnV2_pJEx	This study	N/A
pDonor_RB‐TnV2_placO1/pL	This study	N/A
pHelper_AraC/pBad‐Tnpase (cam^R^)	This study	N/A
pHelper_TetR/pTet‐Tnpase (cam^R^)	This study	N/A
pHelper_TetR/pTet‐Tnpase (carb^R^)	This study	N/A
Additional plasmids and sequence information	This study	Dataset EV1
**Oligonucleotides and other sequence‐based reagents**		
Nextera XT Index Kit v2 Set A (96 indexes, 384 samples)	Illumina	FC‐131‐2001
NGS primers and adapters	This study	Appendix Table S1
**Chemicals, Enzymes, and other reagents**		
NEBuilder HiFi DNA Assembly Master Mix	New England Biolabs	E2621L
NEBNext Ultra II FS DNA Library Prep with Sample Purification Beads	New England Biolabs	E6177L
NEBNext Ultra II Q5 Master Mix	New England Biolabs	M0544L
OneTaq Hot Start 2X Master Mix with Standard Buffer	New England Biolabs	M0484L
Q5® Hot Start High‐Fidelity 2X Master Mix	New England Biolabs	M0494L
BbsI‐HF	New England Biolabs	R3539L
BsmBI‐v2	New England Biolabs	R0739L
FseI	New England Biolabs	R0588L
SbfI‐HF	New England Biolabs	R3642L
T4 DNA Ligase	New England Biolabs	M0202L
Carbenicillin disodium salt	Sigma‐Aldrich	C1389‐5G
Chloramphenicol	Sigma‐Aldrich	C0378‐100G
D‐cycloserine	Sigma‐Aldrich	C6880‐5G
Kanamycin sulfate	VWR	0408‐25G
Bacto Dehydrated Agar	BD	DF0140‐01‐0
LB Broth, Miller	BD	BP1426‐2
M9, Minimal Salts, 5X	SERVA	48505.01
Terrific Broth (TB)	BD	DF0438‐17
Arbutin	Sigma‐Aldrich	A4256‐10G
Methyl β‐D‐glucopyranoside (bMDG)	Sigma‐Aldrich	M0779
glycyl‐L‐glutamic acid	Sigma‐Aldrich	851604
L‐serine	Sigma‐Aldrich	S4311‐25G
Anhydrotetracycline (ATc) hydrochloride	Supelco	37919‐100MG‐R
L‐(+)‐Arabinose	ThermoFisher	A11921.18
Crystal Violet (CV)	Fisher Scientific	C581‐25
Isopropyl β‐d‐1‐thiogalactopyranoside (IPTG)	Sigma‐Aldrich	I6758
**Software**		
Python v3.6.13		https://www.python.org/
Bio‐Tradis	Barquist *et al* (2016)	https://github.com/sanger‐pathogens/Bio‐Tradis
Custom Tn‐Seq and RB‐Tn‐Seq analysis pipelines	This study	https://github.com/maalcantar/tn‐seq_data_analysis
GraphPad Prism 9		https://www.graphpad.com/
Fastp v0.23.2	Chen *et al* (2018)	https://github.com/OpenGene/fastp
Bowtie2 v2.4.4	Langmead & Salzberg (2012)	https://bowtie‐bio.sourceforge.net/bowtie2/index.shtml
UMI‐tools v1.1.0	Smith *et al* (2017)	https://github.com/CGATOxford/UMI‐tools
MACS v3.0.0a7	Zhang *et al* (2008)	https://github.com/macs3‐project/MACS
BEDTools v2.30.0	Quinlan & Hall (2010)	https://bedtools.readthedocs.io/en/latest/
Seqkit v2.2.0	Shen *et al* (2016)	https://bioinf.shenwei.me/seqkit/
Fastq‐pair v0.4		https://github.com/linsalrob/fastq‐pair
Bartender v1.1	Zhao *et al* (2018)	https://github.com/LaoZZZZZ/bartender‐1.1
**Other**		
GenCatch Plasmid DNA Mini‐Prep Kit	Epoch Life Sciences	2160250
DNeasy Blood & Tissue Kit	Qiagen	69504
Monarch DNA Gel Extraction Kit	New England Biolabs	T1020L
Magnetic Separation Rack	New England Biolabs	S1515S
CLARIOstar Plus	BMG Labtech	
SpectraMax M5 plate reader	Molecular Devices	
Qubit 3.0 Fluorometer	ThermoFisher	
T100 Thermal Cycler	Bio‐Rad	
Biolog EcoPlates	Biolog	

### Methods and Protocols

#### Strains and routine plasmid construction

The underlying genetic part sequences used to construct the plasmids in this study were obtained from the following plasmids: the TetR/pTet‐regulated *himar1C9* was from pHdCas9, a gift from Harris Wang (Addgene plasmid #137080); the AraC/pBAD‐regulated *himar1C9* was from pSAM_AraC, a gift from Harry Mobley (Addgene plasmid #91569); the *mariner* transposon flanked with MmeI‐modified inverted repeats was derived from pSAM_Ec, a gift from Matthew Mulvey (Addgene plasmid #102939). Transposon‐mediated mutagenesis and evolution experiments were performed in two *E. coli* K‐12 strains: MG1655, and its genome‐reduced derivative MDS42 (Pósfai *et al*, 2006).

To construct the pHelper plasmids, we used NEB Turbo *E. coli* competent cells. pDonor plasmids harboring an R6Ky origin of replication were assembled in Invitrogen OneShot PIR2 competent cells (*pir* ^+^). All plasmids were constructed with a combination of NEB HiFi assembly protocols and restriction‐ligation cloning, except those described in Fig 4 and Appendix Fig S9, which were constructed using the Golden Gate assembly platform. Following transformation of the chemically competent cloning strains, colonies were isolated on LB agar containing either 50 μg/ml chloramphenicol, 50 μg/ml kanamycin, or 100 μg/ml carbenicillin. Diagnostic colony PCR reactions were performed in NEB OneTaq HotStart 2x Master Mix with sample loading dye, and 2–5 positive colonies were identified by resolving the amplicons on 1% agarose gels with SYBR Safe (ThermoFisher). The clones were grown overnight in 6 ml LB with the corresponding antibiotic selection, and plasmid DNA was extracted with a GenCatch™ Plasmid DNA Mini‐Prep Kit (Epoch Life Sciences). Finally, each plasmid prep was Sanger sequenced to verify the insert and backbone sequences (Quintara Biosciences). The plasmids constructed and used in this work will be available upon request to the corresponding author.

#### Growth media and inductions

Standard cloning procedures and nonselective outgrowths were performed using Miller LB broth (Fisher), with 15 g/l Bacto agar (BD) added for solid media. Unless otherwise stated, we used kanamycin (50 μg/ml), carbenicillin (100 μg/ml), and chloramphenicol (20 μg/ml) for antibiotic selections.

For the carbon source utilization experiments described in Figs 3 and 4, we used M9 minimal media supplemented with different carbon sources in powdered form or added directly to the Biolog EcoPlates. To make 1 l of the base media, we combined 200 ml Difco 5× M9 minimal salts solution (BD), 1 ml MgSO_4_ (1 M), 0.3 ml CaCl_2_ (1 M), and 10 ml of a trace elements stock solution (100×). To make the 100× trace elements stock solution, we added the following (in order) to 800 ml of filtered water: 5 g EDTA, 498 mg FeCl_3_ (anhydrous), 84 mg ZnCl_2_, 765 μl CuCl_2_·2H_2_O (0.1 M), 210 μl CoCl_2_·6H_2_O (0.2 M), 1.6 ml H_3_BO_3_ (0.1 M), and 8.1 μl MnCl_2_·4H_2_O (1 M) before bringing the total volume to 1 l and sterilizing over a 0.22 μm filter. NaOH was used to adjust the pH to 7.5 immediately after the EDTA was added. For the downstream experiments with either L‐serine (6–100 g/l), glycyl‐L‐glutamic acid (12 g/l), or β‐methyl‐D‐glucoside (4.0 g/l), we incorporated the carbon source in powdered form to the base media before bringing the volume to 1 l and sterilizing over a 0.22 μm filter.

To make the nonselective M9‐glucose, we added 1 ml biotin (1 mg/ml), 1 ml thiamin (1 mg/ml), and 20 ml glucose (20% w/v stock) to the base media before bringing the volume to 1 l and sterilizing over a 0.22 μm filter. For the D‐cycloserine and arbutin growth experiments, we used the MT mineral salts medium described by Hall (1998). This consists of 423 mg sodium citrate, 100 mg MgSO_4_·7H_2_O, 1 g (NH_4_)_2_SO_4_, 540 mg FeCl_3_, 1 mg thiamine, 3 g KH_2_PO_4_, and 7 g K_2_HPO_4_, with either glucose (2.0 g/l) or arbutin (1.0 g/l) as carbon sources. For the arabinose induction experiments, we combined arabinose (1.0 g/l) and glycerol (2.0 g/l). In the arbutin growth experiments, we used glucose (1.0 g/l) and glycerol (2.0 g/l) as the carbon source.

Unless otherwise stated, the transcriptional inducers used in this study were added to growth media at the following concentrations: anhydrotetracycline (ATc), 50 ng/ml; arabinose, 0.1% w/v; isopropyl β‐D‐1‐thiogalactopyranoside (IPTG), 200 μM; and crystal violet (CV), 0.25 μM. The ATc and IPTG stock solutions were stored frozen at −20°C in 100% ethanol and water, respectively.

#### Transformation and D‐cycloserine assays

For the arabinose‐inducible transposase system, *E. coli* strains harboring an AraC/pBAD‐controlled *himar1C9* transposase on a medium copy p15A plasmid were transformed with pDonor plasmid molecules (10 ng) and rescued for 1 h at 37°C in either SOC (uninduced), or SOB+0.2% arabinose (induced). We compared two transposon constructs: an unmodified donor containing a *neoR*/*kanR* cassette (labeled Tn in the text) and a larger variant containing *kanR* and *mNeonGreen* expression cassettes (labeled Tn[GFP] in the text). Four hundred and fifty microliter from each transformation was then spread on two replicate plates. The colonies were counted after overnight growth at 37°C. For the ATc‐inducible system, *E. coli* strains harboring a TetR/pTet‐controlled *himar1C9* transposase on a medium copy p15A plasmid were transformed with donor plasmid molecules (10 ng) and rescued for 1 h at 37°C in either SOC, or SOC + 100 ng/ml ATc. Four hundred and fifty microliter from each transformation was then plated on two replicate plates. The colonies were counted after overnight growth at 37°C.

The D‐cycloserine growth assay presented in Fig 1D was performed by transforming MDS42 harboring a TetR/pTet‐controlled *himar1C9* transposase on a pHelper plasmid with the two transposon variants described above: Tn and Tn[GFP]. Individual colonies were picked and inoculated into a 96‐deep‐well plate with 300 μl terrific broth (TB, Difco™) and antibiotics per well (*n* = 24 per variant, per duplicate transformation). We also included two controls: the parental MDS42 strain (*n* = 48), and MDS42 (*n* = 24) with the pHelper plasmid. After an overnight incubation at 37°C, the TB plate was passaged into two parallel plates containing 300 μl MT minimal salts media with 0.1% glucose and 0.2% glycerol per well, at a dilution of 1:100. One MT‐glucose/glycerol plate was supplemented with ATc (50 ng/ml). The cultures were grown for 8 h at 37°C and then passaged into a final pair of selection plates containing 400 μl MT‐glucose/glycerol media and 20 μM D‐cycloserine, with or without ATc. Importantly, each culture was diluted 10,000‐fold into the selection media at this step to create a population bottleneck, and selection was maintained with kanamycin (25 μg/ml) and chloramphenicol (25 μg/ml). Statistical comparisons between end‐point OD values were carried out in R v3.6.1 using the wilcox.test function with paired = TRUE. The resultant *P*‐values were corrected for multiple hypothesis testing by using the p.adjust function with method = “fdr” (Benjamini–Hochberg; Benjamini & Hochberg, 1995).

#### Arbutin growth experiments

In a preliminary experiment (Appendix Fig S2A), we transformed both *E. coli* MG1655 and MDS42 cells harboring either a TetR/pTet‐ or AraC/pBAD‐regulated *himar1C9* transposase helper plasmid (pHelper) with a donor plasmid harboring the Tn‐pOUT construct. Individual colonies from the transformations were picked alongside the respective pHelper controls and the parental strain and inoculated into 300 μl of TB (Difco™) in individual wells of a 96‐deep‐well plate. Throughout this experiment, we used both chloramphenicol and kanamycin at 25 μg/ml. After growth overnight at 37°C with shaking at 900 rpm, we passaged the wells into 300 μl nonselective MT minimal salts media at a dilution of 1:100. For the variants with the TetR/pTet‐regulated transposase, the nonselective MT media contained glucose (1.0 g/l) and glycerol (2.0 g/l) as the carbon source, and duplicate plates were incubated for ~8 h either with or without 50 ng/ml ATc (i.e., induced or uninduced conditions). For the variants with the AraC/pBAD‐regulated transposase, the duplicate plates contained either MT media with glucose (1.0 g/l) and glycerol (2.0 g/l) as the uninduced state, or arabinose (1.0 g/l) and glycerol (2.0 g/l) as the induced state. Similarly, these duplicate plates were incubated for ~8 h at 37°C with shaking at 900 rpm (Infors Multitron 2 shaking incubator). After passaging, the wells of the TB overnight plate were supplemented with 300 μl of 50% glycerol and frozen at −80°C. Following treatment with or without inducer, the cultures were passaged at a ratio of 1:2,000 into 400 μl fresh MT media containing arbutin (1.0 g/l) by first diluting each well 10‐fold in PBS. These cultures were incubated for 5 days at 37°C, and high‐growth phenotypes were documented based on the characteristic color change of the media (Appendix Fig S2B and C). For the arabinose‐inducible system, the MT‐arbutin media were incompatible with sustained induction. However, for the ATc‐inducible system, we maintained 50 ng/ml throughout. Focusing specifically on the ATc‐inducible *tetR* variant of the pHelper, we repeated this experiment by reinoculating fresh TB plates from the original glycerol stock plates and grew them overnight at 37°C. We then passaged the wells into duplicate plates of 300 μl MT media with glucose (1.0 g/l) and glycerol (2.0 g/l) at a ratio of 1:100, either with or without inducer and incubated them at 37°C for ~8 h. We then passaged 3 μl per well from both the induced and uninduced plates into two intermediate conditions: 300 μl total of MT‐arbutin and MT‐glucose/glycerol mixed at ratios of either 3:1 or 19:1. After a 24‐h incubation, these four plates were then finally passaged into 100% MT‐arbutin media at a ratio of 1:100 and incubated for 5 days at 37°C. On the second day, 200 μl of fresh media was added to each well. On the fifth day, end‐point OD600 measurements were taken with a CLARIOstar Plus plate reader (BMG Labtech; Appendix Fig S3D and E).

The experiment presented in Fig 2A was performed in a similar way, starting with the inoculation of single colonies into the wells of a 96‐well deep‐well plate and then passaging the cultures through the following conditions (dilution factors for each passage are provided in brackets): 8 h in 400 μl TB, 8 h in 400 μl MT‐glucose/glycerol (1:100 dilution), 24 h in MT‐arbutin:MT‐glucose/glycerol mixed at a 1:1 ratio (1:100 dilution), 24 h in MT‐arbutin:MT‐glucose/glycerol mixed at a 3:1 ratio (1:100 dilution), 24 h in MT‐arbutin:MT‐glucose/glycerol mixed at a 19:1 ratio (1:100 dilution), and 48 h in 100% MT‐arbutin (1:100 dilution). For each of the 24‐h incubations, end‐point OD600 readings were taken using a SpectraMax M5 plate reader. During the final selection in MT‐arbutin, intermediate OD600 readings were taken by removing 100 μl of culture per well and replacing the volume with 100 μl of appropriate fresh media. A final end‐point OD600 reading was taken at 48 h.

The growth curves shown in Fig 2D and E were obtained by inoculating 100 μl of MT‐arbutin media (containing 50 ng/ml ATc) with 1 μl of the appropriate ATc + culture following the 24‐h incubation on MT‐arbutin:MT‐glucose/glycerol mixed at a 19:1 ratio. In all the experiments described in this section, parental MG1655 and MDS42 strains were maintained without antibiotic. pHelper strains expressing the transposase only were maintained with chloramphenicol. Transposon insertion strains were maintained with both chloramphenicol and kanamycin.

#### Biolog EcoPlate carbon source assays

Chemically competent MDS42 cells (50 μl) containing a pHelper plasmid with a chloramphenicol resistance marker were transformed in triplicate with 20 ng of the pDonor plasmids Tn and Tn‐pOUT and plated on LB agar with dual chloramphenicol (20 μg/ml) and kanamycin (50 μg/ml) selection. One colony from each of the triplicate plates was inoculated into 6 ml of M9‐glucose minimal media with ATc (50 ng/ml), chloramphenicol (20 μg/ml), and kanamycin (50 μg/ml), alongside three control cultures inoculated from untransformed MDS42‐pHelper cells with ATc (50 ng/ml) and chloramphenicol (20 μg/ml). After 16 h of growth, the nine cultures were passaged into identical, fresh media at a dilution of 1:100 and grown for a further 8 h. The cultures were diluted to a concentration of 10^6^ cfu/ml, and then 1 μl was aliquoted into the wells of three Biolog EcoPlates such that each plate contained 32 wells corresponding to MDS42 pHelper, 32 wells corresponding to MDS42 pHelper Tn, and 32 wells corresponding to MDS42 pHelper Tn‐pOUT spanning each unique carbon source, respectively. The wells of the Biolog EcoPlates contained 100 μl of M9 salts media (without carbon source), supplemented with 50 ng/ml ATc. To reduce the potential burden imposed by antibiotic selection in this preliminary screen, we used chloramphenicol (10 μg/ml) and kanamycin (25 μg/ml) to maintain selection for the transposase and transposon, respectively.

The three replicate plates were incubated at 37°C with shaking at 900 rpm and 90% humidity. OD600 measurements were taken every 6–12 h using a SpectraMax^®^ M5 plate reader. After 24 h of growth, 1 μl of culture was taken from each well and used to inoculate fresh triplicate plates at a dilution of 1:100. This process was repeated every 24 h for a total of three passages, with OD600 measurements continuing in parallel for both freshly inoculated and ancestral plates (Appendix Fig S5). A glycerol stock of the single MDS42 pHelper Tn‐pOUT replicate that showed detectable growth on L‐serine (EVOL‐1) was stored at −80°C.

#### L‐serine growth experiments

A glycerol stock of EVOL‐1 was used to streak out colonies on LB agar with chloramphenicol (20 μg/ml) and kanamycin (50 μg/ml) selection. For comparison, we also grew out stocks of MDS42 pHelper, MG1655 pHelper, MDS42, and MG1655. Eight colonies were picked from each of the five strains (EVOL‐1 plus four controls) and grown up overnight in M9‐glucose media in a 96‐deep‐well plate at 37°C. These overnight cultures were then each inoculated 1:300 into two different parallel sets of conditions: 300 μl M9‐glucose with either 24, 50, 75 or 100 g/l L‐serine (Fig 3C), and 300 μl M9 salts only with 6, 12, 24, or 50 g/l L‐serine (Fig 3B). These cultures were grown for 24 h, with 100 μl aliquots removed for OD600 measurements at 13 and 23 h using a CLARIOstar Plus plate reader (BMG Labtech).

To assess the reproducibility of this adaptation process, we repeated the transformation process for the original carbon source screen: chemically competent MDS42 cells (50 μl) containing a pHelper plasmid with a chloramphenicol resistance marker were transformed with 20 ng of the pDonor plasmids Tn or Tn‐pOUT and plated on LB agar with dual chloramphenicol (20 μg/ml) and kanamycin (50 μg/ml) selection. To control for any effects of the antibiotic selection, we performed the same transformation with chemically competent MDS42 cells (50 μl) containing a pHelper plasmid with an ampicillin/carbenicillin resistance marker. Sixteen colonies from each of the six MDS42 strains—Tn and Tn‐pOUT with either pHelper(cam^R^) or pHelper(carb^R^), and the parental controls pHelper(cam^R^) and pHelper(carb^R^)—were inoculated into 300 μl M9‐glucose minimal media with 50 ng/ml ATc in a 96‐deep‐well plate and incubated at 37°C for 10 h. These cultures were then passaged into 300 μl of identical fresh media at a dilution of 1:300 and grown overnight. After this second passage in M9‐glucose minimal media, 3 μl of the overnight cultures were inoculated into two parallel plates of M9 salts media with 300 μl of 12 g/l L‐serine per well either with or without 50 ng/ml ATc. Appropriate antibiotic selection was maintained throughout with chloramphenicol (20 μg/ml) or carbenicillin (100 μg/ml), and kanamycin (50 μg/ml) for strains containing the transposon. At 32‐h intervals, each plate was passaged into an identical one containing 300 μl per well of fresh media at a dilution of 1:100, with a final OD600 measurement taken after 50 h of growth in the third passage (Fig 3D).

For each of the two strains with detectable growth at this end‐point [MDS42 Tn‐pOUT pHelper(cam^R^) and MDS42 Tn‐pOUT pHelper(carb^R^)], we stored four separate replicates for Tn‐Seq and paired these with the corresponding “start‐point” cultures from the first round of nonselective growth. We then pooled all the replicates for each strain at the start‐point (six pools), and all the replicates in the two growth‐positive strains at the end‐point (two pools). This total of 24 samples (8 independent populations at matched start‐ and end‐points, plus 6 start‐point pools and 2 end‐point pools) were prepped for Tn‐Seq.

#### Tn‐Seq sample preparation and sequencing

To generate Tn‐seq data for the experiments in Figs 2, 3, 4, we used a modified version of the Tn‐seq protocol described by Palani (2019). First, we purified genomic DNA (gDNA) from cell pellets (fresh, or flash‐frozen and stored at −80°C) using a Qiagen DNeasy Blood & Tissue Kit following the manufacturer's instructions. We included an Rnase A treatment and eluted samples in 100 μl AE buffer. We then fragmented 100 ng gDNA using an NEBNext Ultra II FS DNA Library Prep with Sample Purification Beads, again following the manufacturer's instructions. As described by Palani (2019), at the ligation step we replaced the standard NEB adapter with 2.5 μl of a custom, preannealed Nextera‐compatible adapter (sENG‐020 and sENG‐021, Appendix Table S1, 15 μM) containing an 8 N unique molecular identifier (UMI, Appendix Table S1). We then performed a 0.8× SPRI selection, eluting in 17 μl of 0.1× TE buffer and transferring 15 μl of this to a new tube. We set up 50 μl enrichment PCR reactions with 15 μl of adapter‐ligated gDNA template, 5 μl each of the forward and reverse primer (sENG‐022 and sENG‐023, Appendix Table S1, 10 μM), and 25 μl of NEBNext Ultra II Q5 Master Mix. Using a T100 Thermal Cycler (Bio‐Rad), the samples were incubated as follows (heated lid on): 98°C 30 s, (98°C 10 s, 65°C 75 s) × 12 cycles, 65°C 5 min, 4°C hold. We then performed a 0.9× SPRI selection, eluting in 17 μl of 0.1× TE buffer and transferring 15 μl of this to a new tube. We measured the concentration of DNA in a 1 μl aliquot of each sample using a Qubit3 fluorimeter (ThermoFisher) and set up indexing PCR reactions with 10 ng of template (made up to 7.5 μl), 2.5 μl each of a unique pair of i5 and i7 primers from a Nextera XT Index Kit (Illumina), and 12.5 μl NEBNext Ultra II Q5 Master Mix. The samples were incubated as follows (heated lid on): 98°C 30 s, (98°C 10 s, 65°C 90 s) × 10 cycles, 65°C 5 min, 4°C hold. We then performed a final 1.2× SPRI selection on each sample, eluting in 30 μl 0.1× TE buffer. We used 2 and 4% TAE‐agarose gels to check the size distributions of the amplified products relative to a low‐molecular‐weight DNA ladder (NEB) and verified the absence of primer‐dimer bands (~128 bp). After measuring the concentrations of each sample, we then pooled them by equal mass and submitted them for sequencing by Quintara Biosciences (Cambridge, MA). Samples were run in paired‐end mode on a MiSeq (2 × 150 cycles, 1 M reads) or HiSeqX (2 × 150 cycles, 300 M reads) with a 15% PhiX spike‐in. All primers used for Tn‐Seq sample preparation and sequencing are included in Appendix Table S1.

#### Tn‐seq analysis

Raw paired‐end sequencing reads were first trimmed, quality filtered, and tagged for UMIs using fastp (Chen *et al*, 2018) v0.23.2 with parameters “‐U —umi_loc=read2 —umi_len=9 —trim_front1=27 —trim_front2=17.” To locate transposon insertion sites, we mapped filtered reads with Bowtie2 (Langmead & Salzberg, 2012) v2.4.4 against reference genomes for *E. coli* MG1655 (U00096.3) or MDS42 (AP012306.1). In brief, we created bowtie indices from each reference genome using the “bowtie2‐build” function with default parameters. We then mapped paired‐end reads to a reference genome using the “bowtie2” function with parameters “—sensitive‐local —maxins 1000 —no‐mixed —no‐discordant —no‐unal.” We chose the maximum insertion parameter based on the size distribution of sequences observed on a 2% agarose gel prior to sequencing. Additionally, only read pairs in which both reads concordantly aligned to the reference genome were considered in subsequent analyses. We deduplicated mapped read pairs based on both their UMI and mapping coordinate using the UMI‐tools (Smith *et al*, 2017) v1.1.0 “dedup” function with parameters “—umi‐separator=: ‐‐paired.”

To locate genomic sites enriched with transposon insertions, we used the Model‐based Analysis of Chip‐Seq (MACS) v3.0.0a7 peak calling algorithm (Zhang *et al*, 2008). Specifically, we identified genomic sites with significantly higher read coverage compared with background, within samples, using the “macs3 callpeaks” function with parameters “‐f BAMPE ‐g 5e6 ‐B ‐q 0.01.” Peaks were associated with their nearest genes using the BEDTools (Quinlan & Hall, 2010) v2.30.0 “closest” function. We plotted peaks and their corresponding fold‐change over background in Python v3.6.13 using Matplotlib v3.2. The most highly enriched peaks were labeled with their nearest gene. If two genes were equally proximal to a peak, only one gene name was used as the label. Lastly, we calculated the distribution of transposon insertion events by counting the number of times forward and reverse reads mapped to each position in the genome. The mapping counts were normalized by the total number of mapped reads.

Statistical comparisons of transposon enrichment pre and postevolutions were conducted using Bio‐Tradis (Barquist *et al*, 2016). As an input to Bio‐Tradis, we used alignments produced by the SMALT v0.7.6 read mapper (ref: https://www.sanger.ac.uk/tool/smalt‐0/) as this was the only short‐read mapper that did not result in substantial amounts of soft‐clipping, which interferes with the Bio‐Tradis pipeline. We performed alignments using the “smalt map” function with parameters “‐x ‐y 0.96 ‐r ‐1” and deduplicated alignments as described previously. Since the native Bio‐Tradis pipeline only accepts FASTQ files as the initial input, we modified the existing scripts to accept deduplicated BAM files in order to mitigate statistical artifacts created by PCR amplification biases. The resultant insertion site files were then annotated with genomic features using the “tradis_gene_insert_sites” function with default settings. Transposon enrichment pre and postevolution was then compared using the “tradis_comparison.R” function with parameters ‐f ‐t 128, which produced fold‐changes and adjusted *P*‐values for each gene above the detection threshold. We produced volcano plots using the EnhancedVolcano (Blighe *et al*, 2018) v.1.4.0 package in R v3.6.1.

#### Magic pools platform

To facilitate the rapid Golden Gate assembly of transposon donor plasmids, we modified the Magic Pools pipeline described by Liu *et al* (2018). A schematic of the overall design is provided in Fig 4A. In short, the donor plasmid was split into the following functional components: Part 1, a promoter to be used for the expression of the cargo; Part 2, cargo gene(s) for expression from within the transposon; Part 3, a preassembled antibiotic resistance cassette (including promoter, ORF and terminator(s)); Part 4, one end of the transposon including the FseI‐SbfI sites for barcode insertion and the terminal repeat, as well as the backbone components for plasmid cloning (R6K origin of replication, oriT sequence, and ampicillin resistance cassette); Part 5, one end of the transposon with BsmBI sites for pOUT promoter insertion, as well as insulating terminator sequences. A portion of Part 4 (encoding the mariner transposon end) and all of Part 5 were first ordered as gBlocks (IDT). The linear Part4 gBlock fragment was combined with a linear section of plasmid backbone components using NEBuilder HiFi DNA Assembly Master Mix (NEB). Flanking BbsI cut sites for each part were installed by PCR using Q5 Hot Start High‐Fidelity 2X Master Mix (NEB), and the resulting products verified via agarose gel and column purified. Each part was stored as a linear DNA amplicon at −20°C.

We began by assembling the donor plasmid for RB‐TnV2. Part 4 (50 fmol) and all other parts (100 fmol) were combined in a 20 μl Golden Gate assembly reaction, containing 2 μl T4 DNA ligase buffer (NEB), 1 μl T4 DNA ligase (NEB), and 1 μl BbsI‐HF (NEB). The samples were incubated for 60 cycles × (37°C, 5 min → 16°C, 5 min), and then 3 μl was transformed into One Shot PIR1 competent cells (Invitrogen). Correct assembly of the transposon donor plasmid was verified via colony PCR and Sanger sequencing. To assemble the pOUT constructs RB‐TnV2_pJEx and RB‐TnV2 pLacO1/pL, distinct promoter regions were then inserted into the Part 5 segment of RB‐TnV2. We first PCR‐amplified the promoter regions of interest using Q5 Hot Start High‐Fidelity 2X Master Mix (NEB) to insert orientation‐specific BsmBI cut sites, and the resulting products were verified via agarose gel and column purified. Each part was stored as a linear DNA amplicon at −20°C. Golden Gate assembly reactions for pOUT promoter insertion were prepared by combining the plasmid backbone (50 fmol), the promoter amplicon (100 fmol), water (to a total reaction volume of 10 μl), T4 DNA ligase buffer (1 μl, NEB), T4 DNA ligase (0.5 μl, NEB), and (0.5 μl BsmBI‐v2, NEB). The samples were incubated for 60 cycles × (42°C, 5 min → 16°C, 5 min) → 60°C for 5 min, and then 3 μl from each reaction was transformed into One Shot PIR1 competent cells (Invitrogen). Correct promoter insertions were verified by Sanger sequencing.

To create N20 barcodes for each transposon variant, we adapted the protocol described by Wetmore *et al* (2015). In short, the barcode section is ordered as a ssDNA oligo, and then two flanking PCR primers were used to create a double‐stranded barcode amplicon with flanking SbfI‐FseI cut sites for insertion into the transposon backbone. To identify the optimal template concentration, we set the flanking primer concentration to 0.5 μM and varied the barcode template input concentration (0.2, 0.1, 0.02, or 0.01 μM). We then performed PCR reactions with Q5 Hot Start High‐Fidelity 2X Master Mix (NEB), setting the annealing temperature to 64°C and elongation time to 60 s for a total of six cycles to preserve barcode diversity. We found that a starting concentration of 0.02 μM template maximized the barcode input while minimizing the formation of undesired products (e.g., unamplified template, primer dimers, or amplicon oligomers) as determined by agarose gel. Following this optimization, we repeated three 50 μl PCR reactions in parallel and gel‐purified the product bands using a Monarch DNA Gel Extraction Kit (NEB) to create a pool of barcode amplicons. Separately, we set up restriction digest reactions for the different transposon donor plasmids and the barcode amplicon in 50 μl reactions as follows: 2 μg DNA, 5 μl CutSmart Buffer (NEB), 1 μl SbfI‐HF (NEB), and 1 μl FseI (NEB). After incubating the samples for 2 h at 37°C, we gel‐purified the products using a Monarch DNA Gel Extraction Kit (NEB). Finally, we performed separate ligation reactions to insert barcodes from the same digested pool into the different donor backbones, combining template (0.1 pmol), barcode (0.6 pmol), 2 μl T4 DNA ligase buffer (NEB), and 1 μl T4 DNA ligase (NEB) in 20 μl reactions that were then incubated at 16°C overnight.

For each donor variant, we transformed replicates consisting of two aliquots of One Shot PIR1 (Invitrogen) competent cell tubes, plating 4 × 250 μl per tube following rescue with 950 μl of SOC. We then incubated the LB‐agar plates overnight at 30°C, resuspended the colonies in 2.5 ml of LB‐kanamycin (50 μg/ml) per plate, and pooled the resuspension for each donor variant (giving 20 ml LB per ligation reaction). We added this to 250 ml of LB with kanamycin (50 μg/ml) in 1 l flasks and grew the cultures for 1 h at 37°C. After dividing this culture into 50 ml aliquots, we then used Zymo‐spin VI columns (C1013‐10) to perform scaled‐up minipreps on the cell pellet using buffers from a GenCatch Plasmid DNA Mini‐Prep Kit (Epoch Life Sciences).

#### Carbon source utilization experiments

Chemically competent *E. coli* MDS42 cells (50 μl aliquots) harboring a TetR/pTet‐regulated *himar1C9* transposase gene on a helper plasmid were transformed with 20 ng of five different donor plasmids encoding the *mariner* transposon variants (Appendix Fig S8A). This included the original nonbarcoded variants Tn and Tn‐pOUT (used in Figs 1, 2, 3), as well as the three barcoded variants RB‐TnV2, RB‐TnV2_pJEx, and RB‐TnV2_pLacO1/pl. Each transformation was performed in triplicate, and after a 1‐h rescue in 900 μl SOC, 950 μl of the cultures were plated on LB agar with chloramphenicol and kanamycin. For the Tn and Tn‐pOUT samples (compatible with the Tn‐Seq pipeline), eight colonies from each triplicate plate were inoculated into LB media for a total for 24 parallel populations for each transposon variant. We also included 24 wells inoculated from colonies of the parental strain (MDS42 with the helper plasmid) and 24 wells inoculated from colonies of unmodified MDS42 as controls. For the RB‐TnV2, RB‐TnV2_pJEx, and RB‐TnV2_pLacO1/pl samples, three colonies from each triplicate plate were inoculated into LB media for a total for nine parallel populations for each transposon variant. We also included 12 wells inoculated from colonies of the parental strain (MDS42 with the helper plasmid) and 12 wells inoculated from colonies of unmodified MDS42 as controls.

The culture conditions used to evolve these replicate populations toward growth on three carbon sources (12 g/l L‐serine, 12 g/l glycyl‐L‐glutamic acid, and 4 g/l β‐methyl‐D‐glucoside) are described in detail in Appendix Fig S9. In brief, after growing the cultures overnight on LB at 37°C, we split the plates into uninduced (no ATc) and induced (50 ng/ml ATc) replicates and propagated them through two passages in M9‐glucose media. These two parallel branches were then triplicated across the three carbon sources and grown for three passages at 37°C. Importantly, for the transposon variants with the inducible promoters (RB‐TnV2_pJEx and RB‐TnV2_pLacO1/pl), these wells were duplicated into the selective media plates and their inducers (crystal violet and IPTG, respectively) added to one of the two replicates. Growth measurements were taken by removing 100 μl aliquots from each well at the time points indicated, transferring them to a 96‐well clear‐bottom plate, and measuring the OD of each well using a CLARIOstar Plus plate reader (BMG Labtech). After the final time point of the carbon source utilization experiment described in Appendix Fig S8, the wells from each unique combination of transposon variant, carbon source and inducers were pooled, and aliquots used for Tn‐Seq/RB‐Tn‐Seq sample preparation and to prepare glycerol stocks for storage at −80°C.

#### Carbon source switching experiments

The starting points for this evolution experiment were the Tn‐pOUT and RB‐TnV2_pJEx strains that had been pre‐evolved in L‐serine media and subsequently stored as pooled glycerol stocks (Appendix Figs S9 and S10). In the case of the RB‐TnV2_pJEx cultures, these cultures had evolved in the presence of crystal violet (CV). Streaks from four glycerol stocks corresponding to two pooled Tn‐pOUT end‐points (M9 L‐serine, with and without ATc) and two pooled RB‐TnV2_pJEx end‐points (M9 L‐serine + CV, with and without ATc) were plated on LB agar with kanamycin and chloramphenicol. For the Tn‐pOUT samples, 12 colonies were picked from each plate and inoculated into 400 μl M9 L‐serine, in a 96‐deep‐well plate for a total of 24 replicates. For the RB‐TnV2_pJEx samples, 18 colonies were picked from each plate and inoculated into 400 μl M9 L‐serine. A second set of 18 colonies were picked from the same plates and inoculated into 400 μl M9 L‐serine with 0.25 μM CV (Fig 5A). In all cases, the M9 L‐serine was supplemented with kanamycin and chloramphenicol. This represents a further passage in their original selective growth media.

To introduce these pre‐evolved populations into a new environment, the M9 L‐serine plate was passaged twice in M9 media with 4.0 g/l β‐methyl‐D‐glucoside as the carbon source (Fig 5A). At each step, the cultures were diluted 1:100 into 400 μl of the fresh media. The first passage in β‐methyl‐D‐glucoside was incubated for 48 h, while the second was incubated for 24 h. Finally, these cultures were passaged once more in M9 L‐serine for an additional 24 h, before being pooled and prepared for Tn‐Seq or RB‐Tn‐Seq.

#### RB‐Tn‐Seq sample preparation and sequencing

We prepared gDNA samples from cell culture pellets in the same way we prepared samples for Tn‐seq. To modify the NGS sample preparation workflow to include the transposon barcode, we modified the primers described by Wetmore *et al* (2015) and Palani (2019) for enrichment of the transposon‐genome junctions via PCR. Furthermore, we used an adapter and enrichment primer sets designed for compatibility with the NEBNext Multiplex Oligos for Illumina (Dual Index Primers Sets) in an analogous two‐step amplification process. In short, we replaced the standard NEB adapter with 2.5 μl of a custom, preannealed TruSeq‐compatible adapter (sENG‐021 and sENG‐024, Appendix Table S1, 15 μM) containing an 8 N unique molecular identifier (UMI, Appendix Table S1). We then performed a 0.3×/0.15× SPRI selection, eluting in 17 μl of 0.1× TE buffer and transferring 15 μl of this to a new tube. We set up 50 μl enrichment PCR reactions with 15 μl of adapter‐ligated gDNA template, 5 μl each of the forward and reverse primer (sENG‐025 and sENG‐027 for Tn‐Seq donors, or sENG‐030 and sENG‐027, Appendix Table S1 for RB‐Tn‐Seq donors), and 25 μl of NEBNext Ultra™ II Q5 Master Mix. Using a T100 Thermal Cycler (Bio‐Rad), the samples were incubated as follows (heated lid on): 98°C 30 s, (98°C 10 s, 65°C 75 s) × 13 cycles, 65°C 5 min, 4°C hold. We then performed a 0.9X SPRI selection, eluting in 17 μl of 0.1× TE buffer and transferring 15 μl of this to a new tube. We measured the concentration of DNA in a 1 μl aliquot of each sample using a Qubit3 fluorimeter (ThermoFisher) and set up indexing PCR reactions with 10 ng of template (made up to 15 μl), 5 μl each of a unique pair of i5 and i7 primers from a NEBNext Multiplex Oligos for Illumina (NEB), and 25 μl NEBNext Ultra™ II Q5 Master Mix. The samples were incubated as follows (heated lid on): 98°C 30 s, (98°C 10 s, 65°C 90 s) × 11 cycles, 65°C 5 min, 4°C hold. We then performed two 0.9× SPRI size selection steps on each sample, eluting in 30 μl 0.1× TE buffer. We used 2 and 4% TAE‐agarose gels to check the size distributions of the amplified products relative to a low‐molecular‐weight DNA ladder (NEB) and verified the absence of primer‐dimer bands (~128 bp). After measuring the concentrations of each sample, we then pooled them by equal mass and submitted them for sequencing by Quintara Biosciences (Cambridge, MA). Samples were run in paired‐end mode on a MiSeq (2 × 150 cycles, 1 M reads) or HiSeqX (2 × 150 cycles, 300 M reads) with a 15% PhiX spike‐in. All primers used for RB‐Tn‐Seq sample preparation and sequencing are included in Appendix Fig S1.

#### Rb‐Tn‐Seq analysis

We first filtered for read pairs in which the forward read contained the expected transposon terminus sequence, with two mismatches permitted, using the seqkit (Shen *et al*, 2016) v2.2.0 “grep” function with parameters “‐s ‐i ‐m 2 ‐P ‐p CAGACCGGGGACTTATCAGCCAACCTGTTA.” Since this process only returns forward reads, we obtained corresponding reverse reads using fastq‐pair (ref: https://github.com/linsalrob/fastq‐pair) v0.4. Next, we extracted RB‐Tn‐Seq barcodes by locating the sequence between expected barcode flanking regions using the “seqkit amplicon” function with parameters “‐m 4 ‐P ‐s ‐F GATGTCCACGAGGTCTCT ‐R GCCGGCCGTCGACCTGCAGCGTACG ‐r 19:‐26 –bed.” In order to consolidate nearly identical barcode sequences that differ because of sequencing errors, we clustered barcodes using the Bartender (Zhao *et al*, 2018) “bartender_single_com” function with parameter “‐d 2.” Only reads associated with barcodes that matched the expected length (20 bp) were used in subsequent analyses.

After extracting transposon‐derived sequences and their associated barcodes, reads were trimmed, quality filtered, and tagged for UMIs using fastp with parameters “‐U ‐‐umi_loc=read2 ‐‐umi_len=8 ‐‐trim_front1=30 ‐‐trim_front2=0.” We then mapped filtered reads using Bowtie2 and deduplicated alignments as described previously. Every deduplicated alignment was then associated with its most proximal gene using the BEDTools “closest” function. Barcodes were used to demultiplex samples into lineages (i.e., samples from different time points that contain the same RB‐Tn‐Seq barcode). We used barcode‐gene associations to track genes that were enriched in transposon insertions throughout and between lineages.

## Author contributions

**Max A English:** Conceptualization; data curation; software; formal analysis; validation; investigation; visualization; methodology; writing – original draft; writing – review and editing. **Miguel A Alcantar:** Data curation; software; formal analysis; validation; investigation; visualization; methodology; writing – original draft; writing – review and editing. **James J Collins:** Conceptualization; resources; supervision; funding acquisition; visualization; writing – original draft; project administration; writing – review and editing.

## Disclosure and competing interests statement

The authors declare that they have no conflict of interest. JJC is an editorial advisory board member. This has no bearing on the editorial consideration of this article for publication.

## Supporting information



Appendix

Dataset EV1

Dataset EV2

Dataset EV3

Source Data for Appendix

Source Data for Figure 1

Source Data for Figure 2

Source Data for Figure 3

Source Data for Figure 4

Source Data for Figure 5

## Data Availability

The raw sequencing files are publicly from the Sequence Read Archive with accession number PRJNA922969. All code used for analysis are available on GitHub (https://github.com/maalcantar/tn‐seq_data_analysis).
