# Supplementary material for: A self‐propagating, barcoded transposon system for the dynamic rewiring of genomic networks
Source: Mol Syst Biol. 2023 Mar 27;19(6):e11398. doi: 10.15252/msb.202211398 (PMC10258560; doi:10.15252/msb.202211398)
Supplement: Supplementary file 1 — Appendix [file MSB-19-e11398-s008.pdf]

Supplementary Information for:

Title: A self-propagating, barcoded transposon system for the dynamic rewiring of genomic networks

Authors: Max A. English†, Miguel A. Alcantar†, and James J. Collins

Appendix Figures 2
Appendix Figure S1 2
Appendix Figure S2 3
Appendix Figure S3 4
Appendix Figure S4 5
Appendix Figure S5 6
Appendix Figure S6 8
Appendix Figure S7 10
Appendix Figure S8 11
Appendix Figure S9 12
Appendix Figure S10 13
Appendix Figure S11 14
Appendix Figure S12 15
Appendix Figure S13 16
Appendix Figure S14 17
Appendix Figure S15 18
Appendix Figure S16 19
Appendix Figure S17 20
Appendix Figure S18 21
Appendix Figure S19 22
Appendix Figure S20 23
Appendix Figure S21 24
Appendix Figure S22 25

Appendix Tables 26
Appendix Table S1 26

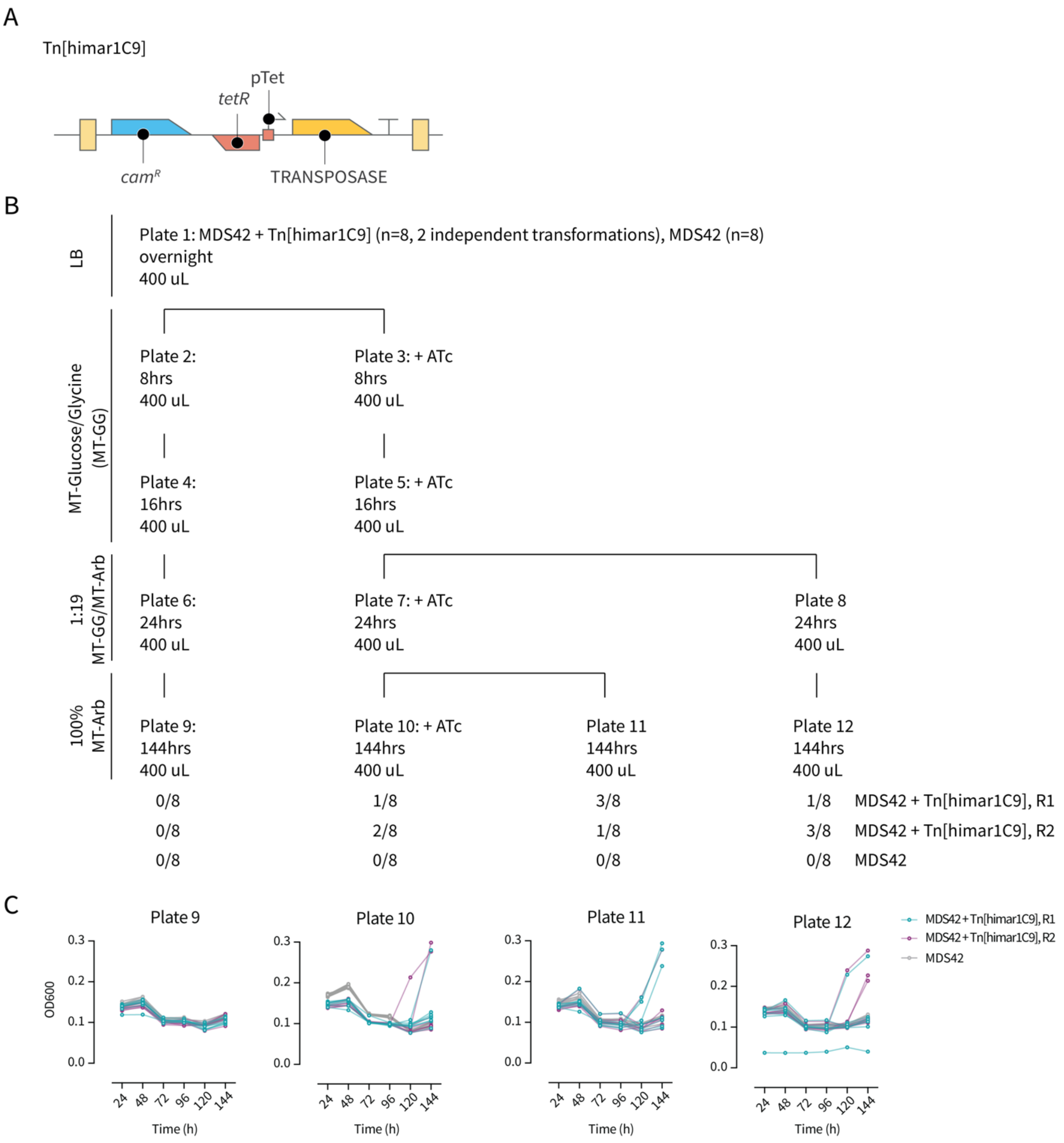

Appendix Figure S1. The impact of an autonomous *mariner* transposon system on the adaptation of *E. coli* MDS42 to arbutin as the sole carbon source. (A) The Tn[himar1C9] transposon construct delivered by direct transformation. (B) Schematic overview of the culture conditions used throughout the course of adaptation to arbutin. The values in the final three rows indicate the proportion of the eight replicates that showed a high-growth phenotype. R1 and R2 correspond to replicates obtained from each of the two independent transformations at the start of the evolution experiment (n=8 unique founder colonies per condition). (C) OD readings taken every 24 hours for the parallel cultures after their introduction to MT-arbutin media. The plate numbers correspond to those described in (B).

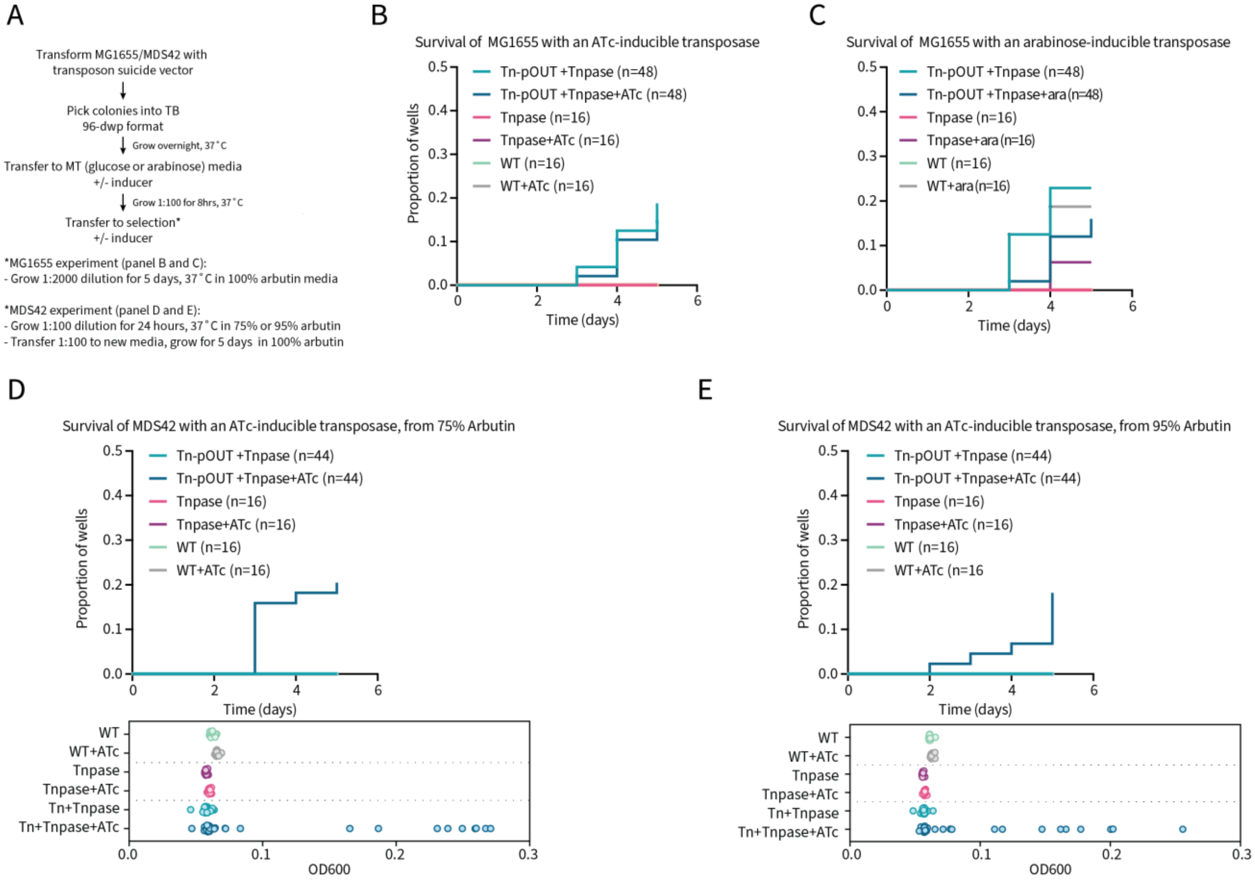

Appendix Figure S2. Preliminary experiments on the evolution of arbutin utilization in *E. coli* MG1655 and MDS42 strains. (A) A schematic overview of the two experiments presented here. In all cases, the transposase was expressed from a medium copy plasmid with a chloramphenicol resistance marker. (B) The proportion of wells of a 96-deep-well plate that showed high-growth phenotypes after their transfer from MT-glucose media (8 hours) to MT-arbutin media (5 days). Plates were inspected every 24 hours for growth. In this experiment, one of two parallel replicate plates had ATc (50ng/ml) added to the MT-glucose and MT-arbutin media. Replicate numbers represent unique founder colonies from a single transformation. (C) The proportion of wells of a 96-deep-well plate that showed high-growth phenotypes after their transfer from either MT-glucose media (uninduced) or MT-arabinose media (induced) (8 hours) to MT-arbutin media (5 days). Plates were inspected every 24 hours for growth. Replicate numbers represent unique founder colonies from a single transformation. (D) The proportion of wells of a 96-deep-well plate that showed high-growth phenotypes after their transfer from MT-glucose media (8 hours) to a 3:1 mixture of MT-arbutin and MT-glucose media (24hrs), and then finally to MT-arbutin media (5 days). Plates were inspected every 24 hours for growth. In this experiment, one of two parallel replicate plates had ATc (50ng/ml) added to the MT-glucose and MT-arbutin media. In the lower panel, endpoint OD measurements are shown for each well in the plate. (E) A similar experiment to (D), in which the intermediate culture conditions was a 19:1 mixture of MT-arbutin and MT-glucose media. Replicate numbers represent unique founder colonies from a single transformation. The founder colonies grown overnight in TB were identical for the replicate cultures in both (D) and (E).

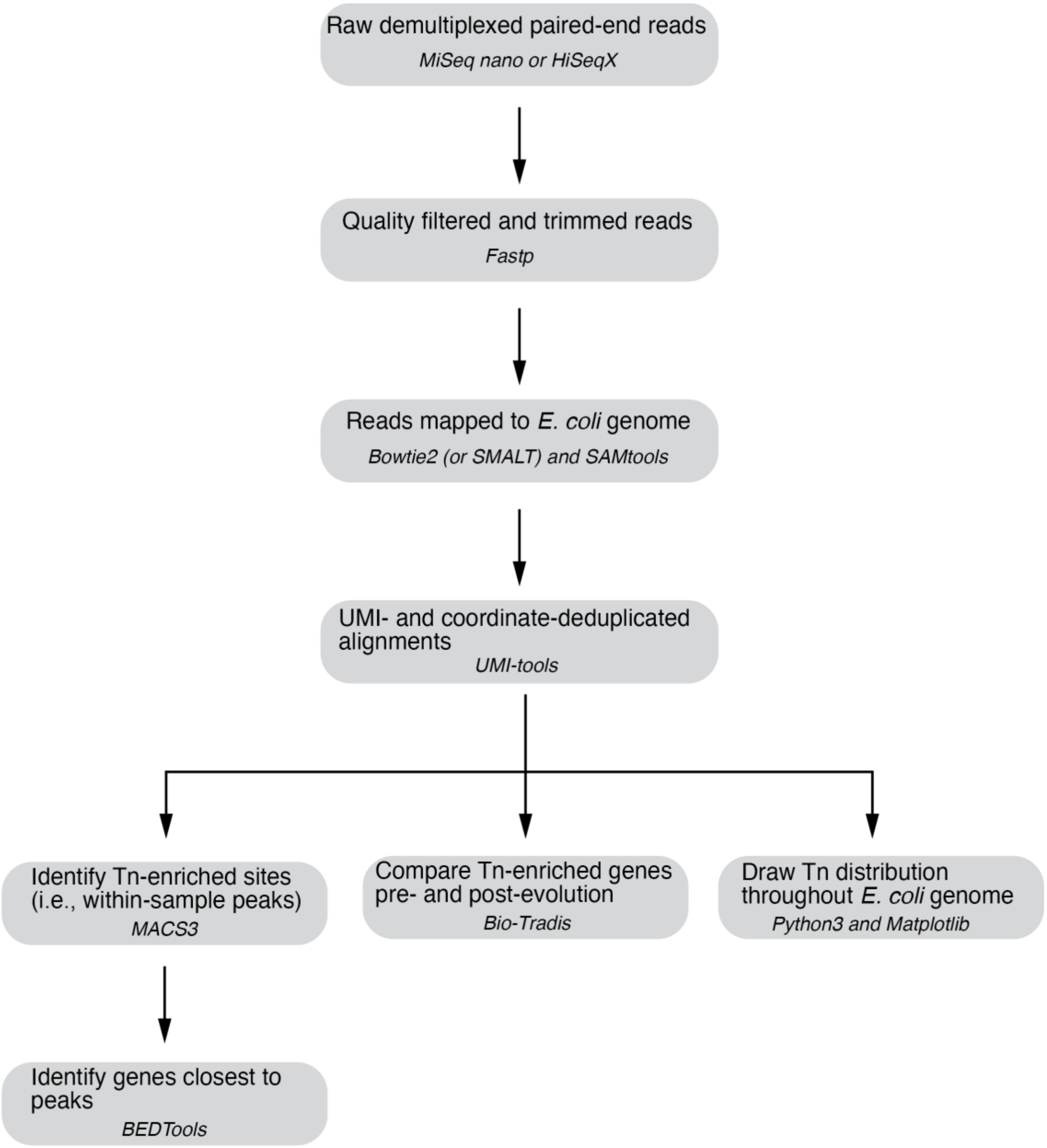

Appendix Figure S3. A schematic representation of the bioinformatics pipeline used to process and analyze the Tn-Seq data presented in Figures 2 and 3. After quality filtering the raw demultiplexed reads, we used Bowtie2 or SMALT (specifically for the Bio-Tradis pipeline) to align the paired reads to the *E. coli* MDS42 genome (AP012306.1). A UMI on the adapter enabled deduplication of the paired reads using both the UMI sequence and mapping coordinate. We then used a series of complementary approaches to identify and annotate peaks corresponding to the enrichment of the transposon-genome junctions.

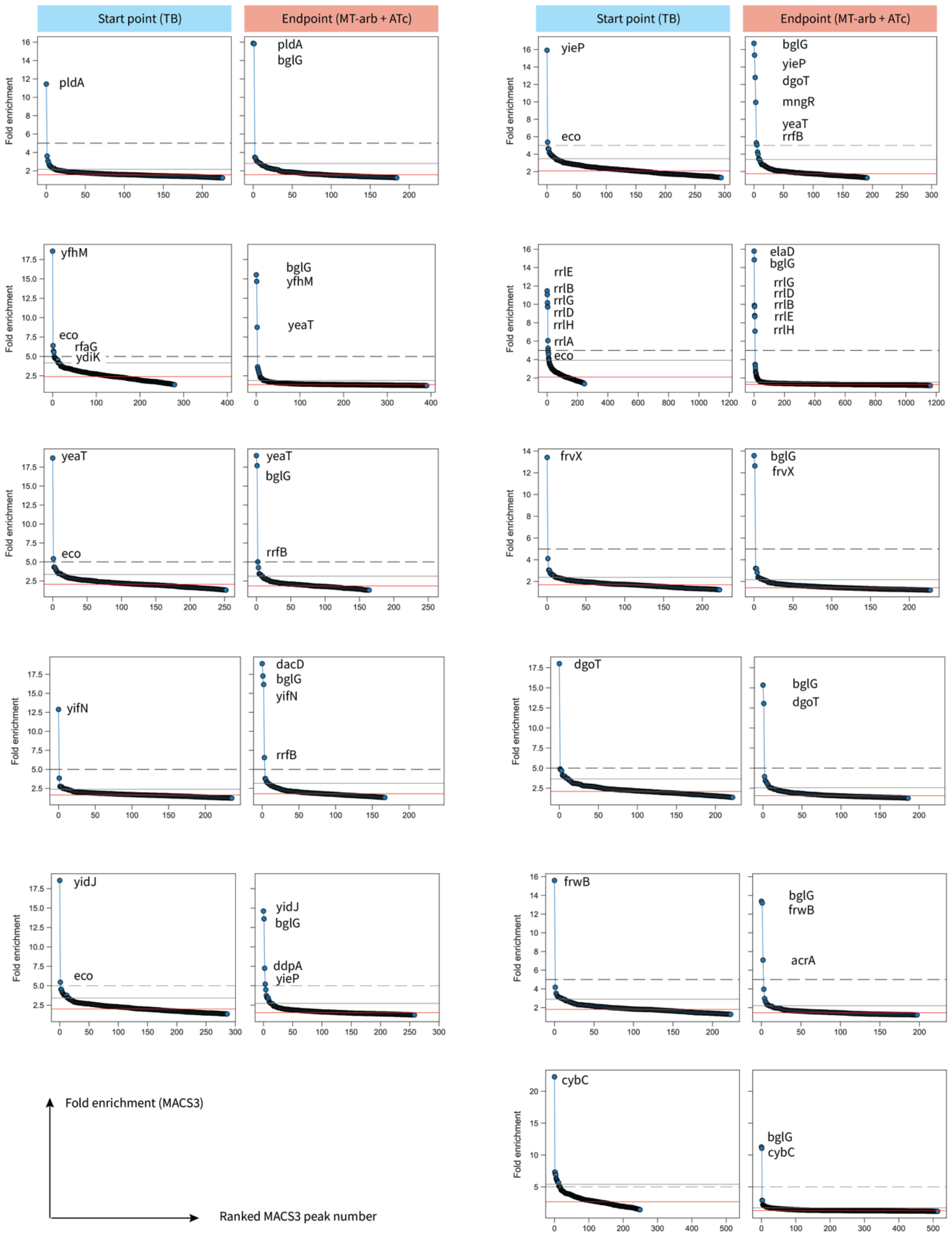

Appendix Figure S4. Plots of the distributions of ranked fold enrichment scores for unique peaks identified using the MACS3 package. Each pair corresponds to Tn-Seq data from individual wells of a 96-well plate either pre- or post-selection (left and right, respectively). The 11 replicates that showed high-growth phenotypes are listed in two columns.

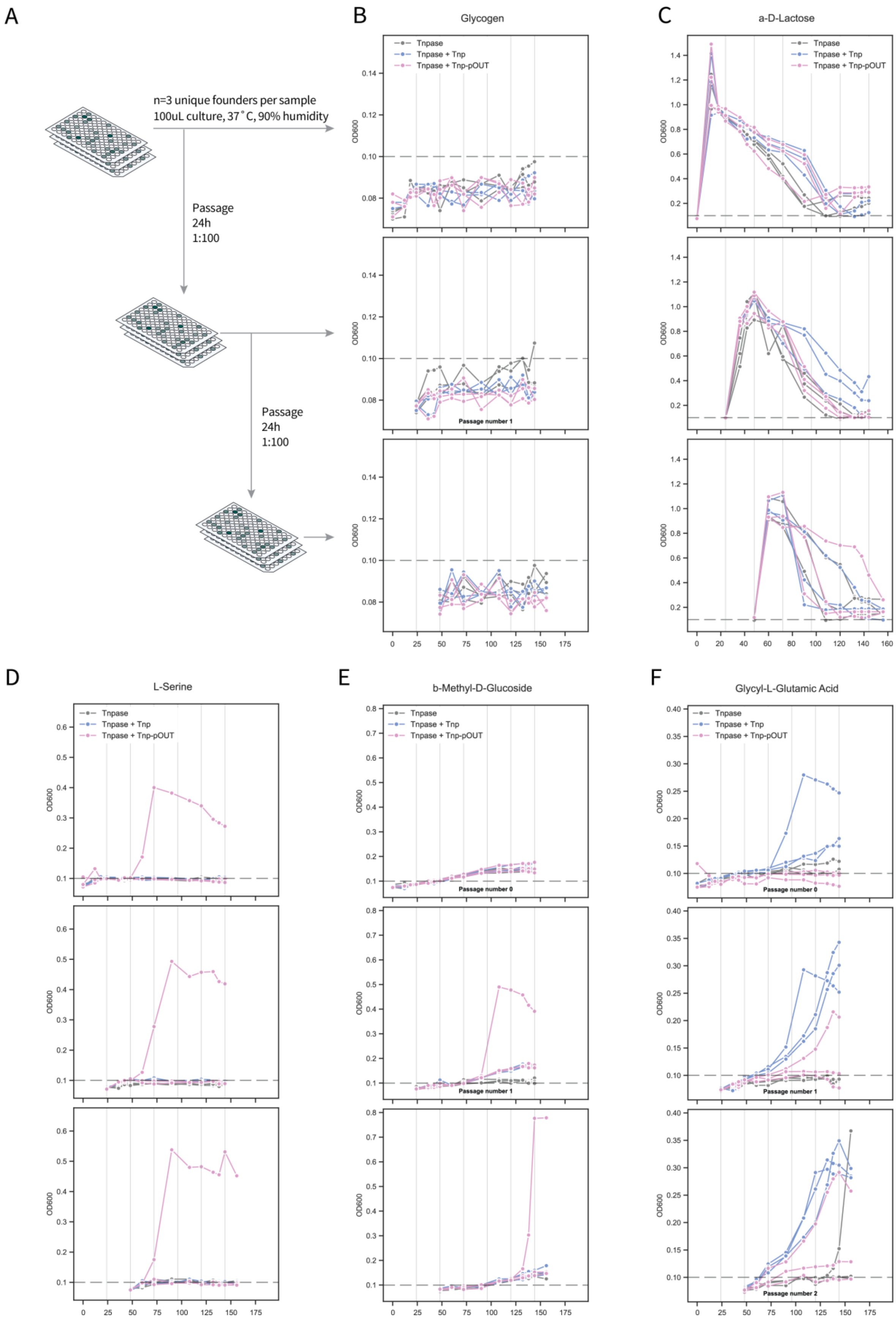
A
n=3 unique founders per sample
100uL culture, 37˚C, 90% humidity
Passage
24h
1:100
Passage
24h
1:100
B
Glycogen
Tnpase
Tnpase + Tnp
Tnpase + Tnp-pOUT
OD600
Passage number 1
C
a-D-Lactose
D
L-Serine
E
b-Methyl-D-Glucoside
Passage number 0
Passage number 1
F
Glycyl-L-Glutamic Acid
Passage number 0
Passage number 1
Passage number 2

Appendix Figure S5. A screen for transposon-dependent growth phenotypes using Biolog EcoPlates with 31 unique carbon sources. (A) A schematic of the passaging process for each of the three *E. coli* MDS42 strains: transposases only (grey, n=3), transposase with an unmodified transposon founder (Tn, blue, n=3), and transposase with an outward-facing promoter transposon (Tn-pOUT, pink, n=3). (B) An example of a carbon source (glycogen) for which there were no observed growth phenotypes within the timeframe of the experiment. (C) An example of a carbon source (α-D-lactose) for which all three replicates of all three strains showed growth phenotypes. (D) Growth on L-serine, showing the emergence of a single Tn-pOUT strain (labelled EVOL-1 in Fig. 3). (E) Growth on ß-methyl-D-glucoside, showing the emergence of a single Tn-pOUT strain. (F) Growth on glycyl-L-glutamic acid, showing the emergence of multiple Tn and Tn-pOUT strains, and a transposase-only strain in the final passage.

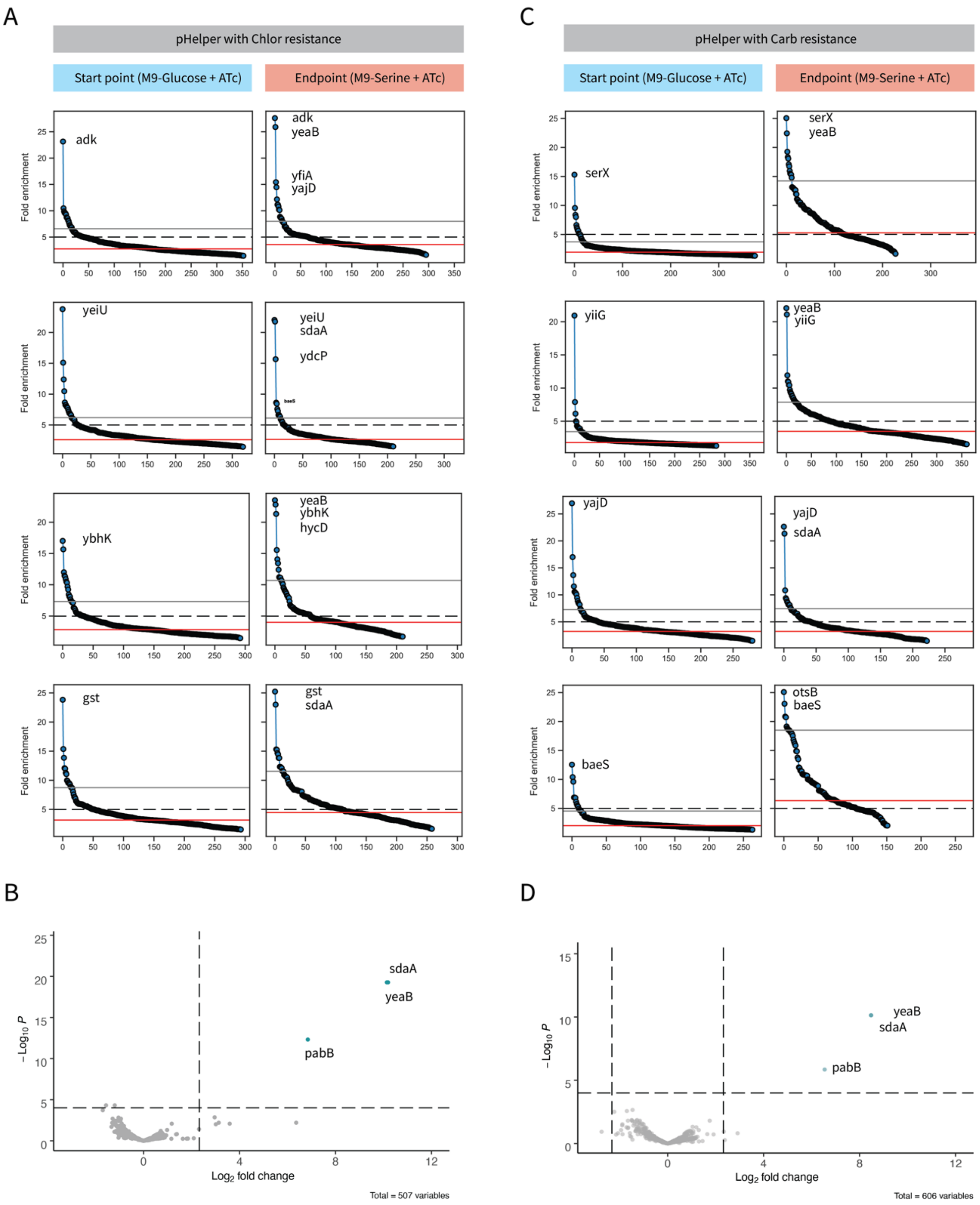

Appendix Figure S6. (A) Plots showing the distributions of ranked fold enrichment scores for unique peaks identified using the MACS3 package. Each pair corresponds to Tn-Seq data from individual wells of a 96-well plate either pre- or post-selection (left and right, respectively). The initial founder insertion is annotated, alongside other highly enriched peaks in the endpoint. (B) A volcano plot showing the differential enrichment of transposon insertions across the genome, calculated by aggregating Tn-Seq data from the four paired replicates in (A). (C) and (D), same as (A) and (B) from an experiment in which the chloramphenicol resistance maker on the pHelper plasmid was replaced with an ampicillin/carbenicillin resistance marker. Differential transposon enrichment analysis was performed using a negative binomial

generalized linear model with Benjamini-Hochberg correction for multiple hypothesis testing utilizing the edgeR package, as implemented in Bio-Tradis.

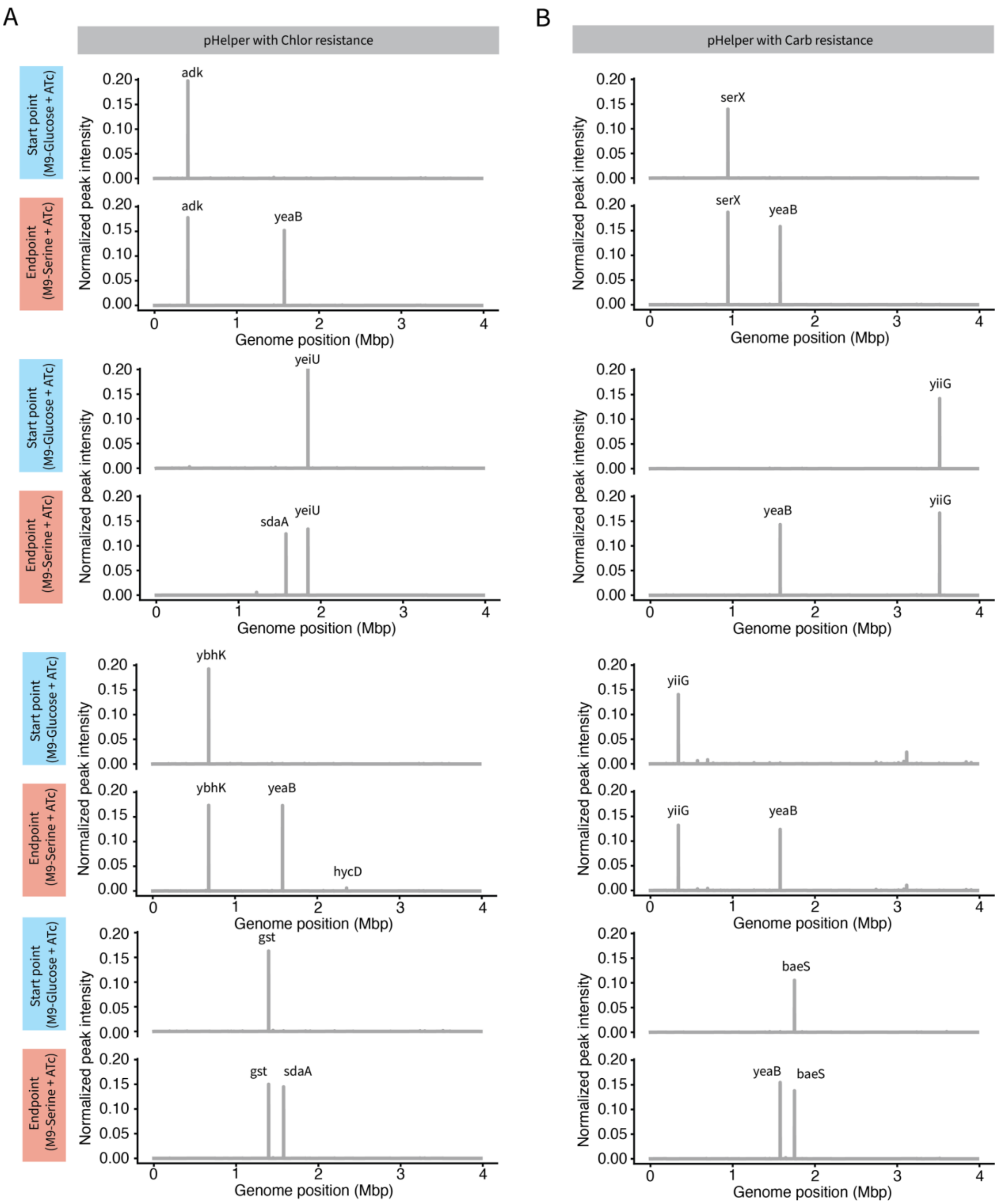

Appendix Figure S7. (A) Genome-wide read alignments showing the normalized number of reads mapping to each position of the *E. coli* MDS42 genome. In each case, the founder peak (pre-selection, as in Appendix Fig. S6) and the dominant secondary peak (post-selection) are annotated with the most proximal gene name. (B) Same as (A), from an experiment in which the chloramphenicol resistance maker on the pHelper plasmid was replaced with an ampicillin/carbenicillin resistance marker.

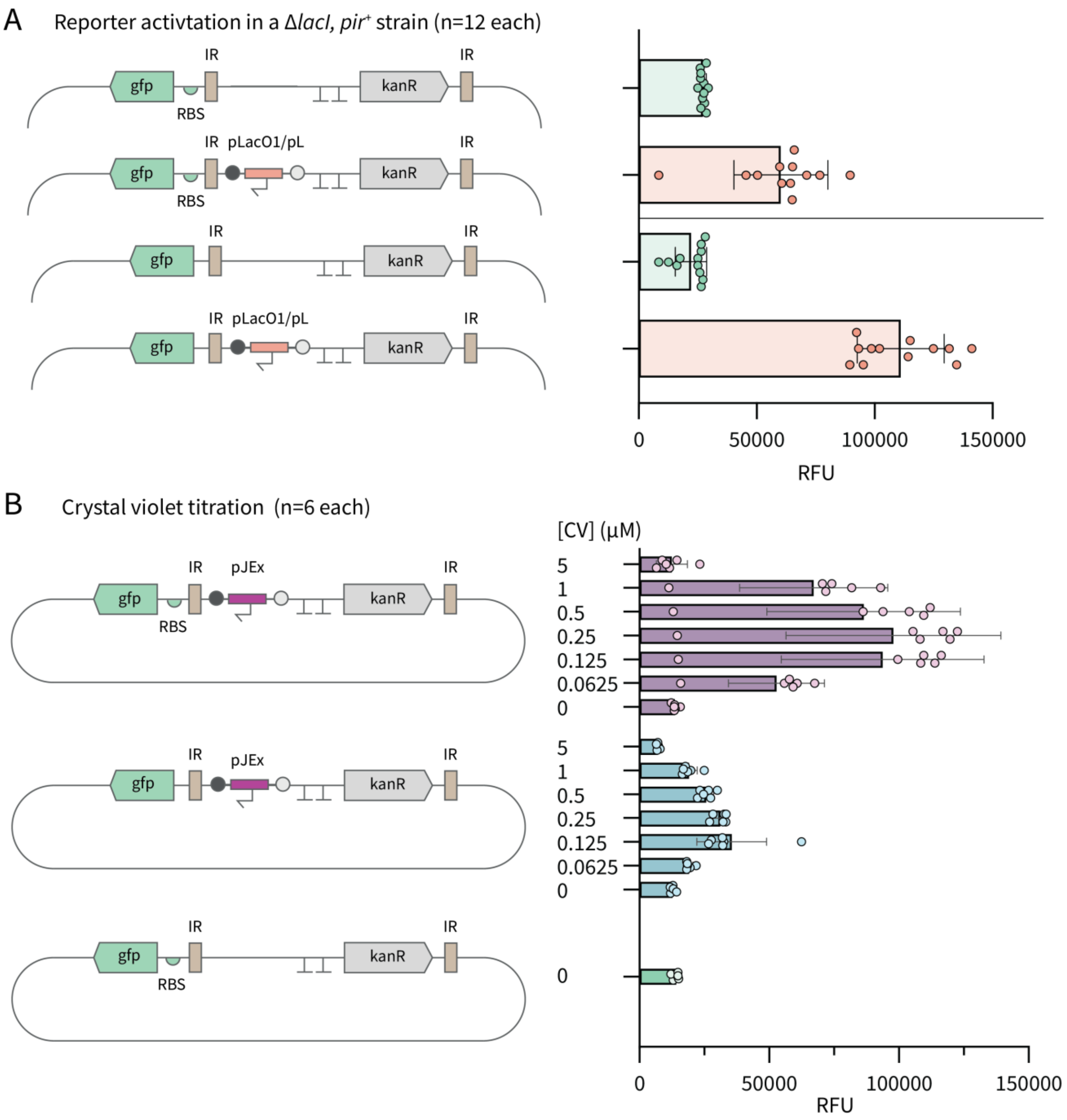

Appendix Figure S8. Validation of the promoter activity of the RB-TnV2_pJEx, and TnV2_placO1/pL transposon variants used in the carbon source utilization experiments. (A) The TnV2_placO1/pL donor plasmid was modified to incorporate a fluorescent *gfp* reporter gene downstream of the transposon end, either with (upper) or without (lower) a ribosome binding site (RBS). The plasmids were transformed into OneShot PIR2 *E. coli* cells (Δ*lacI pir*$^{+}$), which maintain the donor plasmids at ~15 copies per cell. Fluorescence measurements were taken in LB after growth overnight for n=12 replicate colonies from a single transformation. (B) A similar experiment was performed with the RB-TnV2_pJEx donor construct, comparing fluorescence measurements after 6 hours post-induction with a range of crystal violet (CV) concentrations. The data represent n=6 replicate colonies from a single transformation.

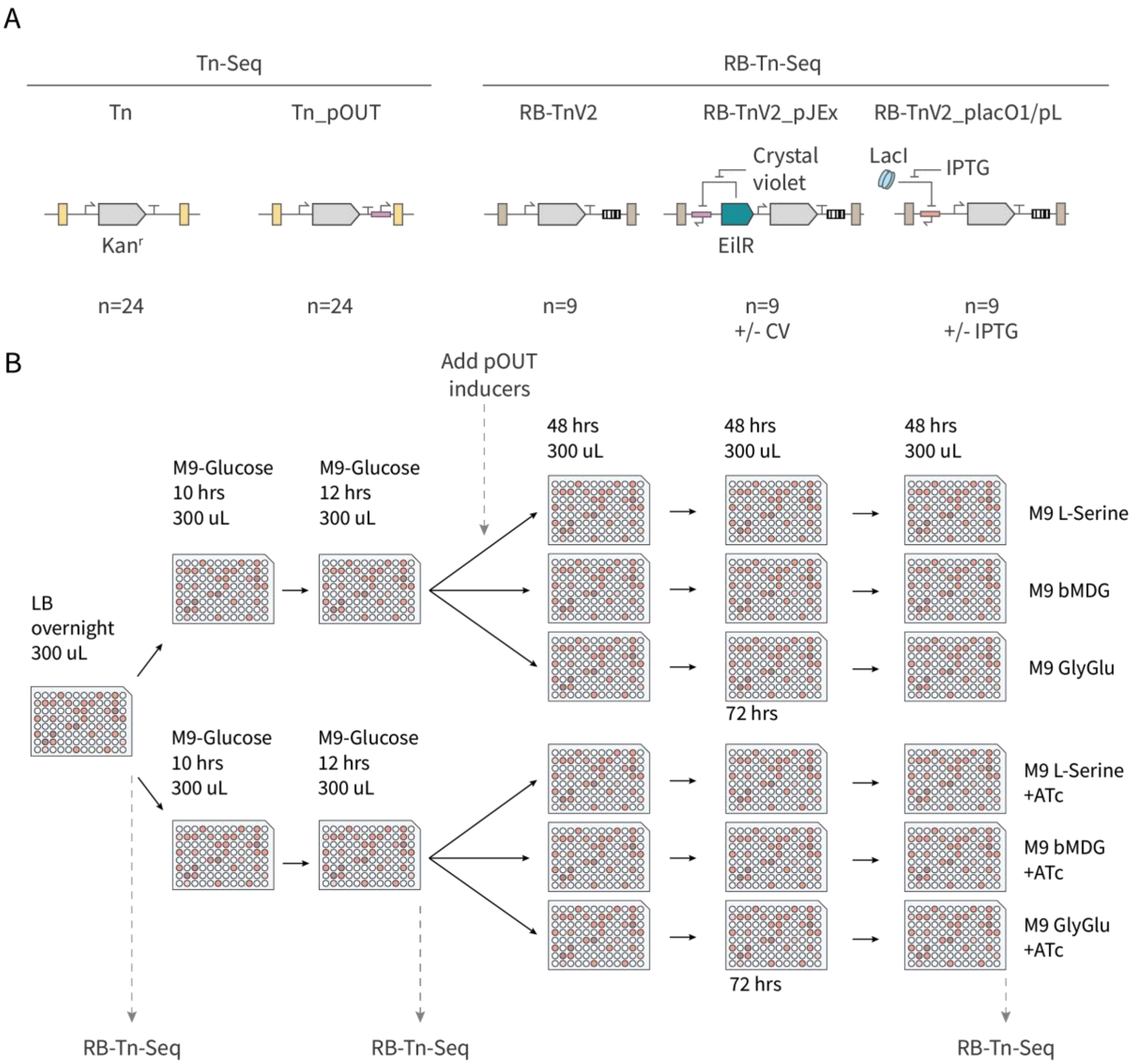

Appendix Figure S9. A schematic overview of the carbon source utilization experiment described in Fig. 4. (A) Diagrams of the different non-barcoded (Tn-Seq, n=24 initial founder colonies) and barcoded (RB-Tn-Seq, n=9 initial founder colonies) transposon variants compared in this assay. The color of the rectangles representing the terminal inverted repeat sequences (yellow or brown) indicate the two different sequences used. (B) A schematic of the individual lineages observed during this experiment, beginning from single colonies inoculated into LB media. For the L-Serine and ß-methyl-D-glucoside (bMDG) conditions, the second passage after adding pOUT inducers was incubated for 48 hours before passaging again. For the Glycyl-L-Glutamic acid experiment, the second passage was incubated for 72 hours before passaging again. The wells corresponding to each unique combination of carbon source, transposon variant, transposase inducer (ATc) and pOUT inducer (CV or IPTG) were pooled at three timepoints and stored for subsequent RB-Tn-Seq sample preparation. CV, crystal violet; IPTG, Isopropyl ß-D-1-thiogalactopyranoside; ATc, anhydrotetracycline.

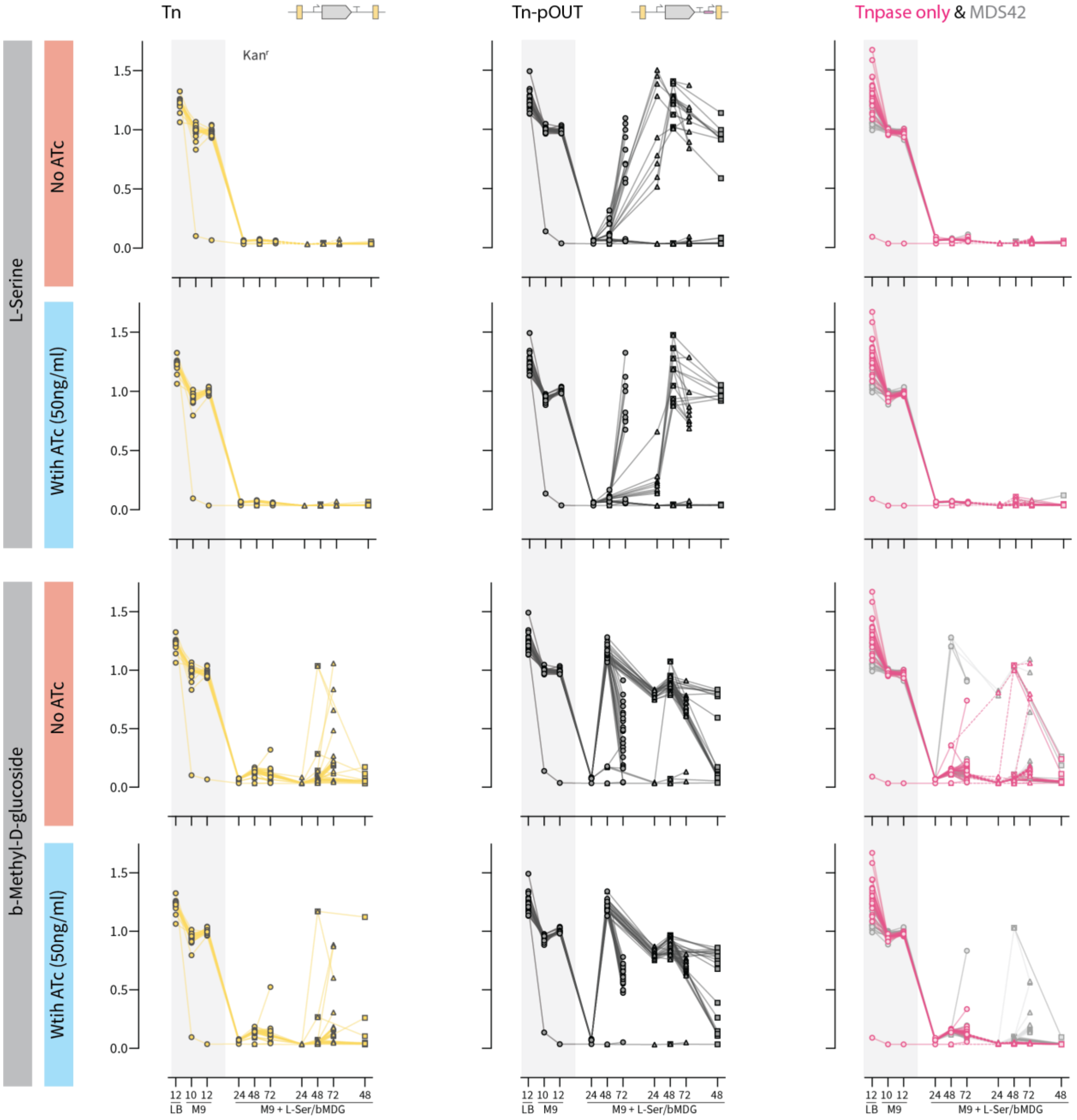

Appendix Figure S10. Longitudinal tracking of growth phenotypes in a carbon source utilization assay. Optical density (OD) measurements were taken at regular intervals for replicate cultures of four *E. coli* MDS42 strains: an unmodified MDS42 strain (light gray, n=24), a derivative expressing the *himar1C9* transposase from a pHelper plasmid (pink, n=24), and unique founders from pHelper strains containing an unmodified *mariner* transposon (Tn, yellow, n=24) or a transposon with an outward facing pJ23104 promoter (Tn-pOUT, dark gray, n=24). Cultures derived from distinct founder colonies were split and passaged through two selective carbon source conditions (L-serine and ß-methyl-D-glucoside) either with or without the transposase inducer (ATc). Grey boxes indicate the initial non-selective growth conditions (LB and M9). The experimental set-up is outlined in detail in Appendix Fig. S9.

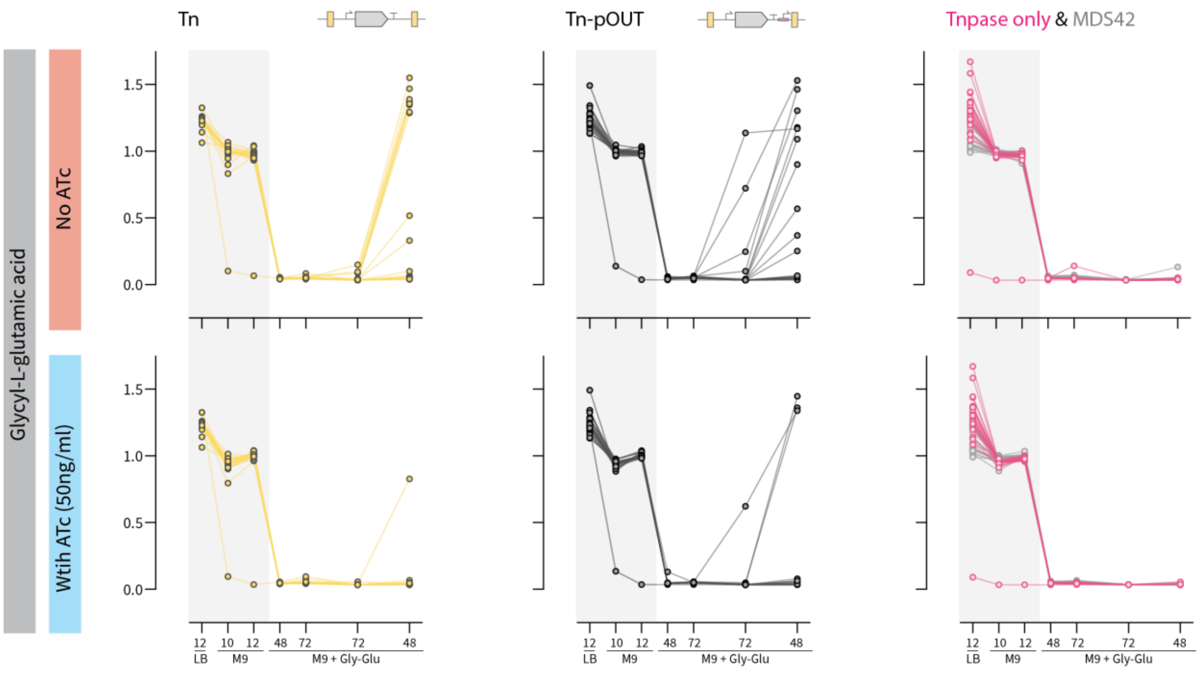

Appendix Figure S11. Longitudinal tracking of growth phenotypes in a carbon source utilization assay. Optical density (OD) measurements were taken at regular intervals for replicate cultures of four *E. coli* MDS42 strains: an unmodified MDS42 strain (light gray, n=24), a derivative expressing the *himar1C9* transposase from a pHelper plasmid (pink, n=24), and unique founders from pHelper strains containing an unmodified *mariner* transposon (Tn, yellow, n=24) or a transposon with an outward facing pJ23104 promoter (Tn-pOUT, dark gray, n=24). Cultures derived from distinct fosunder colonies were incubated in glycyl-L-glutamic acid media either with or without the transposase inducer (ATc). Grey boxes indicate the initial non-selective growth conditions (LB and M9). The experimental set-up is outlined in detail in Appendix Fig. S9.

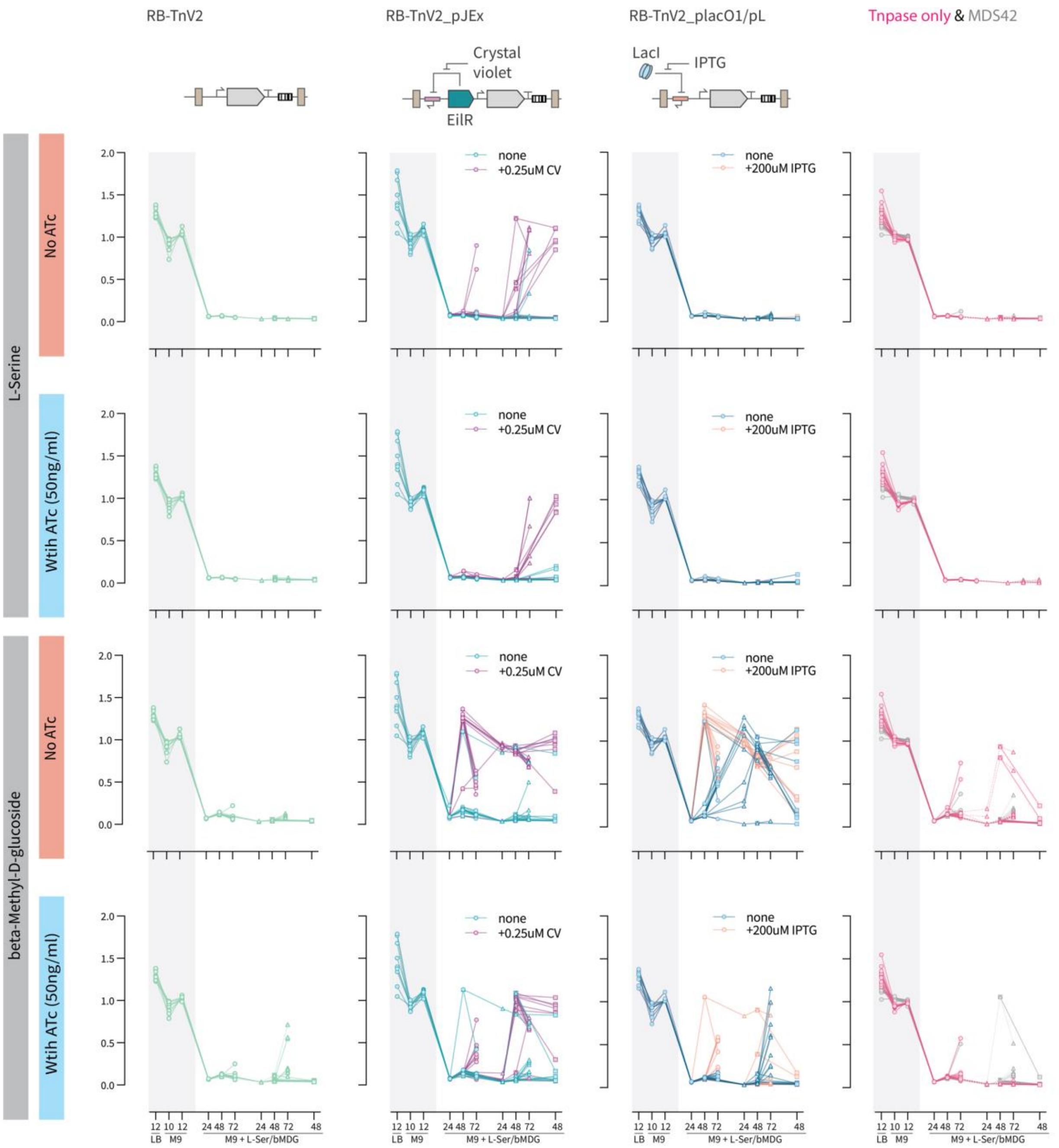

Appendix Figure S12. Longitudinal tracking of growth phenotypes in a carbon source utilization assay. Optical density (OD) measurements were taken at regular intervals for replicate cultures of five *E. coli* MDS42 strains: an unmodified MDS42 strain (light gray, n=24), a derivative expressing the *himar1C9* transposase from a pHelper plasmid (pink, n=24), and unique founders from pHelper strains containing a barcoded *mariner* transposon (RB-TnV2, green, n=9), a transposon with an outward facing pJEx promoter (Tn-RB-TnV2_pJex, turquoise, n=9), or a transposon with an outward facing placO1/pL promoter (Tn-RB-TnV2_ placO1/pL, dark blue, n=9). Cultures derived from distinct founder colonies were split and passaged through two selective carbon source conditions (L-serine and ß-methyl-D-glucoside) either with or without the transposase inducer (ATc) and the pOUT inducers crystal violet (CV) and Isopropyl ß-D-1-thiogalactopyranoside (IPTG). Grey boxes indicate the initial non-selective growth conditions (LB and M9). The experimental set-up is outlined in detail in Appendix Fig. S9.

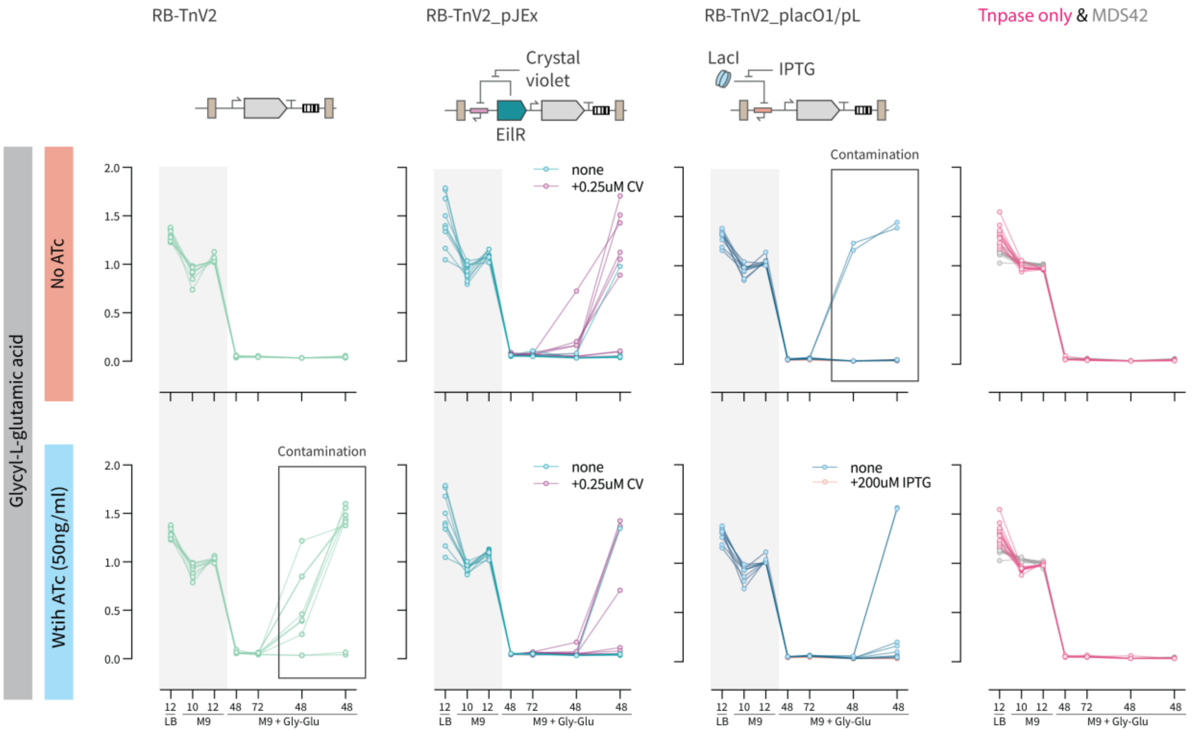

Appendix Figure S13. Longitudinal tracking of growth phenotypes in a carbon source utilization assay. Optical density (OD) measurements were taken at regular intervals for replicate cultures of five *E. coli* MDS42 strains: an unmodified MDS42 strain (light gray, n=24), a derivative expressing the *himar1C9* transposase from a pHelper plasmid (pink, n=24), and unique founders from pHelper strains containing a barcoded *mariner* transposon (RB-TnV2, green, n=9), a transposon with an outward facing pJEx promoter (Tn-RB-TnV2_pJex, turquoise, n=9), or a transposon with an outward facing placO1/pL promoter (Tn-RB-TnV2_ placO1/pL, dark blue, n=9). Cultures derived from distinct founder colonies were incubated in glycyl-L-glutamic acid media either with or without the transposase inducer (ATc) and the pOUT inducers crystal violet (CV) and Isopropyl ß-D-1-thiogalactopyranoside (IPTG). Grey boxes indicate the initial non-selective growth conditions (LB and M9). Cultures in the black boxes labelled “contamination” at the timepoints indicated were identified as having barcodes derived from the RB-TnV2_pJex founders, implying the cross-contamination of these wells. The experimental set-up is outlined in detail in Appendix Fig. S9.

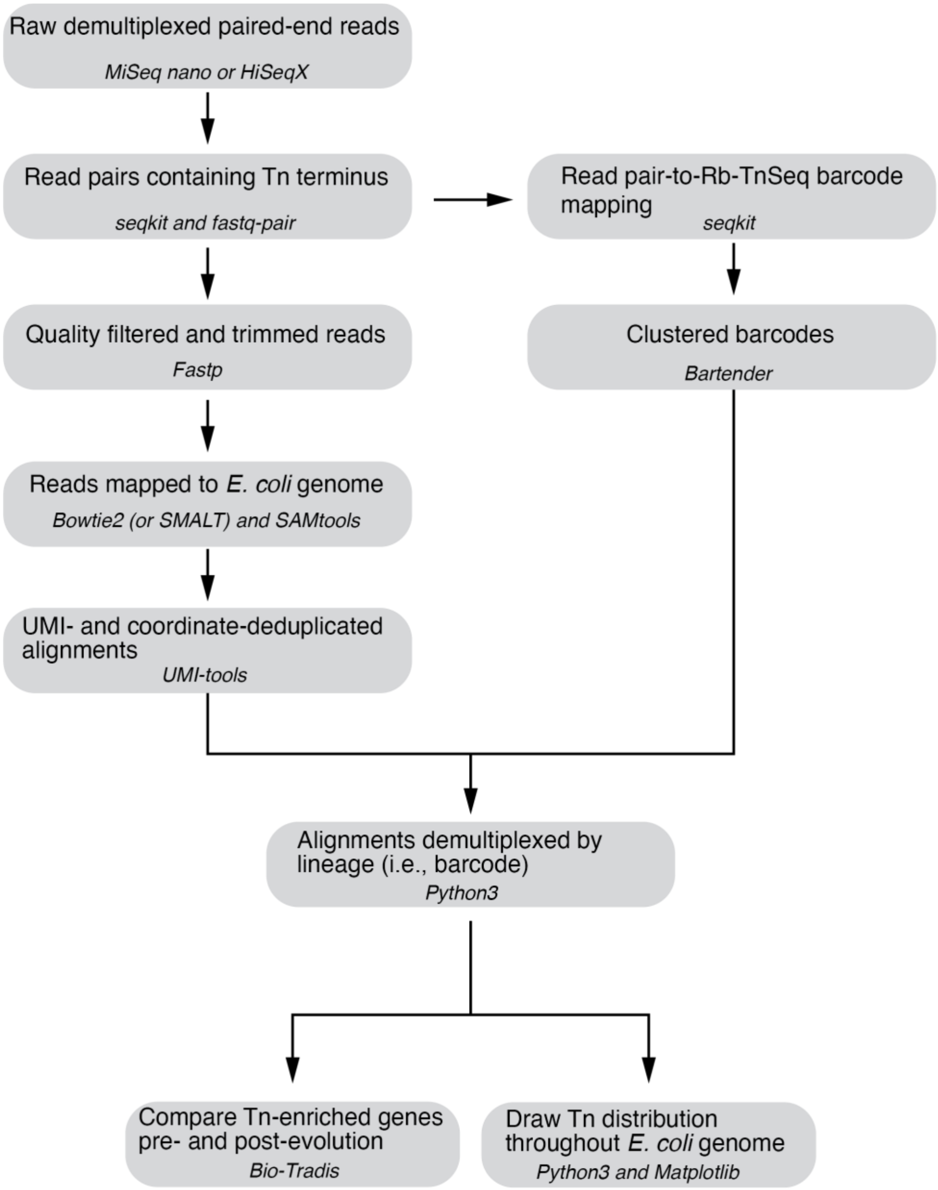

Appendix Figure S14. A schematic representation of the bioinformatics pipeline used for RB-Tn-Seq data processing and analysis. The process is similar to that described in Appendix Fig. S3, but the internal position of the forward primer in the transposon-genome junction PCR allowed for the initial filtering of read pairs to select only those that contained the transposon inverted repeat end sequence. Barcodes (N20) were then extracted based on their conserved flanking regions, and clustered to account for sequencing errors. By cross-referencing the read names, we then annotated the aligned, UMI-deduplicated reads with their corrected barcodes. Demultiplexing was then performed to isolate individual lineages corresponding to the genomic positions of transposons defined by conserved, unique barcodes.

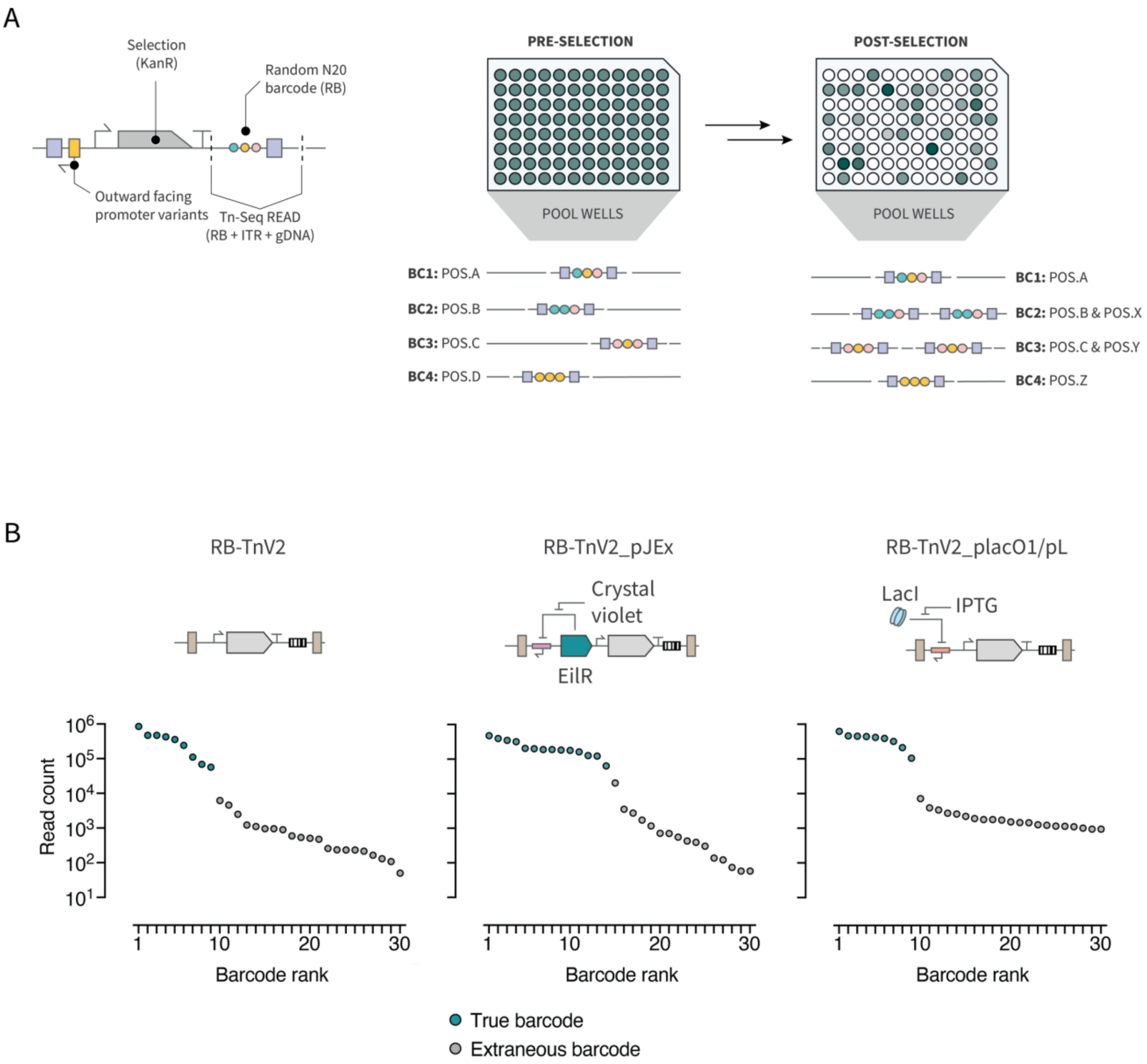

Appendix Figure S15. Replicate demultiplexing and lineage reconstruction from pooled samples from the carbon source utilization experiment described in Appendix Fig. S9. (A) A schematic of the RB-Tn-Seq read structure, allowing for the simultaneous identification of barcode ID and genomic position. Individual cultures from unique founder insertions were grown in arrayed format, before being pooled to increase throughput and reduce sequencing costs. Longitudinal sequencing can then be used to track the changes in the genomic positions of each barcoded transposon variant, thereby reconstructing a lineage for that transposon molecule. (B) The distributions of read counts assigned to the top 30 clustered barcode variants identified in each sample before selection. For RB-TnV2 and RB-TnV2_placO1/pL, the number of replicate founder colony (n=9) matches is reflected in the drop-off in read count after the first nine barcodes. For RB-TnV2_pJex, we identified 14 distinct barcodes suggesting that either some wells received cells from more than one founder colony, or that some colonies were formed from cells transformed with two donor plasmids.

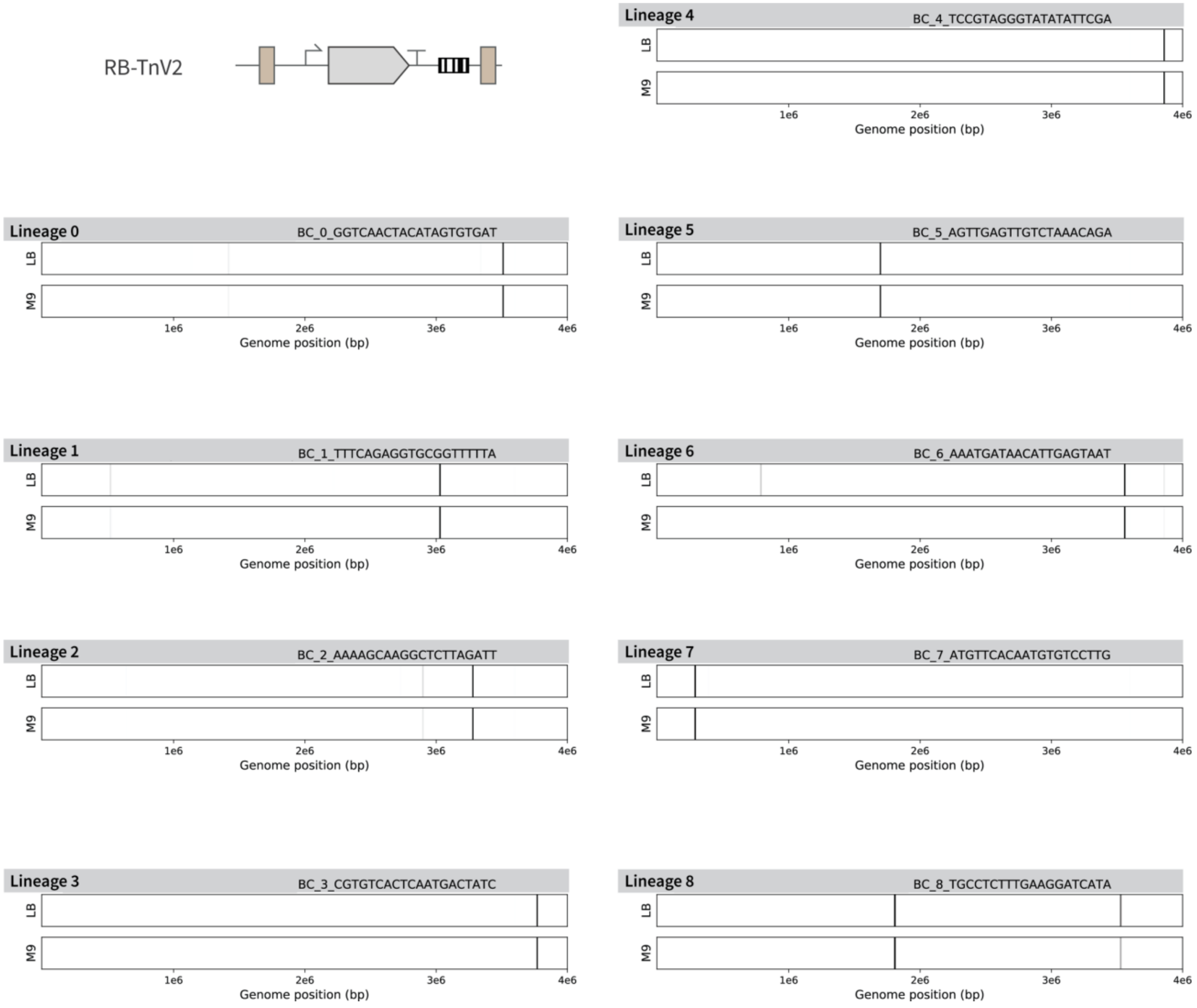

Appendix Figure S16. Heatmap plots for the nine RB-TnV2 lineages showing the genomic locations of transposon insertions based on the RB-Tn-Seq alignments. No growth was observed in any of the wells in the final passage at the 48-hour timepoint, so only data from the non-selective growth conditions (LB and M9-Glucose) are shown. For each lineage, a discrete founder insertion can be identified. High-abundance secondary insertions are also visible in some cultures (e.g., Lineages 2 and 8). A detailed schematic of the experimental set-up is presented in Appendix Fig. S9. The color intensity shows the normalized value of the maximum number of aligned reads for 10kb bins across the genome, set to saturate at a normalized peak intensity of 0.1 to aide in visualization.

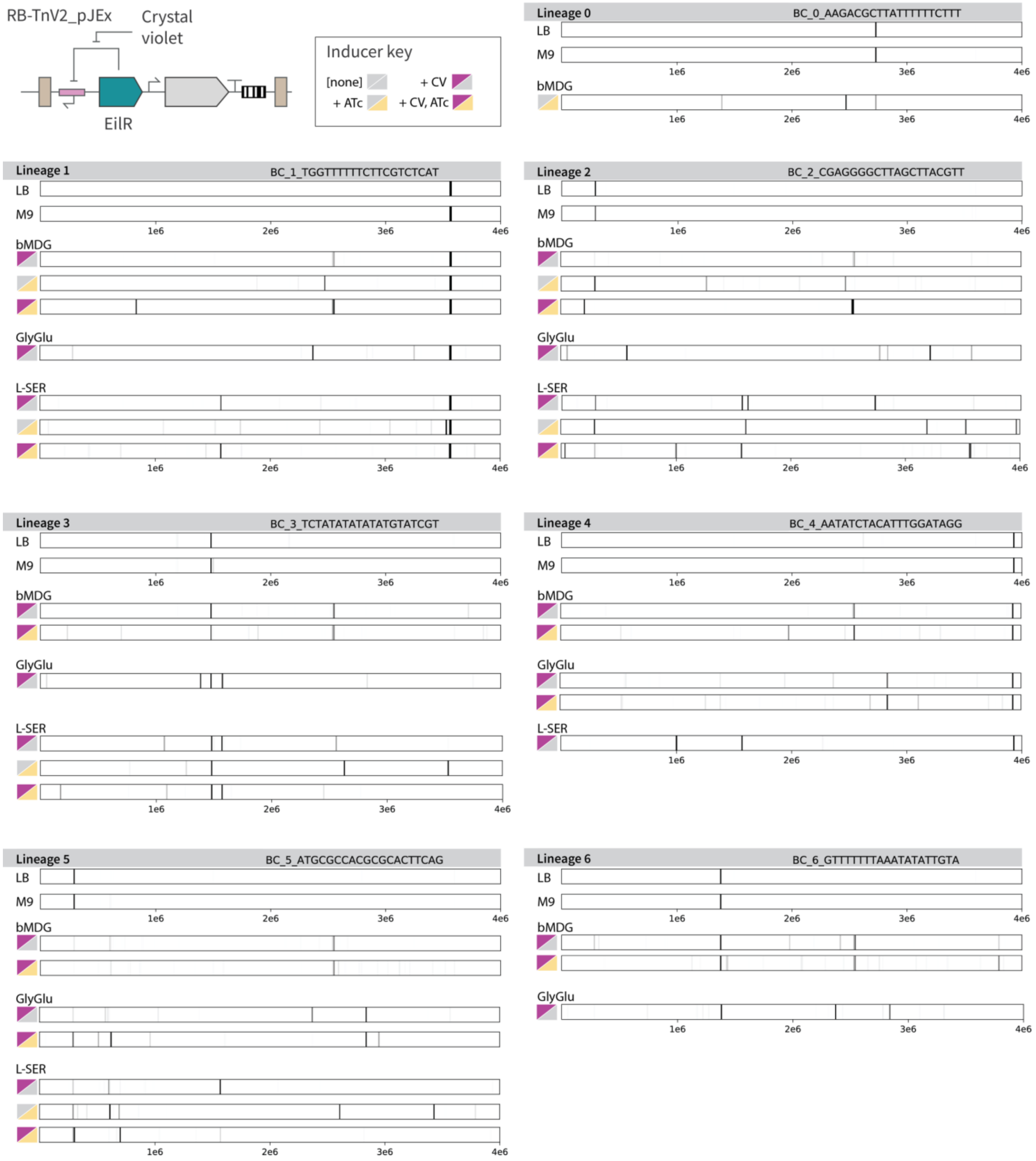

Appendix Figure S17. Heatmap plots for seven of the 14 unique RB-TnV2_pJex lineages showing the genomic locations of transposon insertions based on RB-Tn-Seq alignment data. For each different carbon source, the colored rectangles indicate the inducers added to each culture. L-SER, L-serine; bMDG, ß-methyl-D-glucoside; GlyGlu, glycl-L-glutamic acid. A detailed schematic of the experimental set-up is presented in Appendix Fig. S9. The color intensity shows the normalized value of the maximum number of aligned reads for 10kb bins across the genome, set to saturate at a normalized peak intensity of 0.1 to aide in visualization.

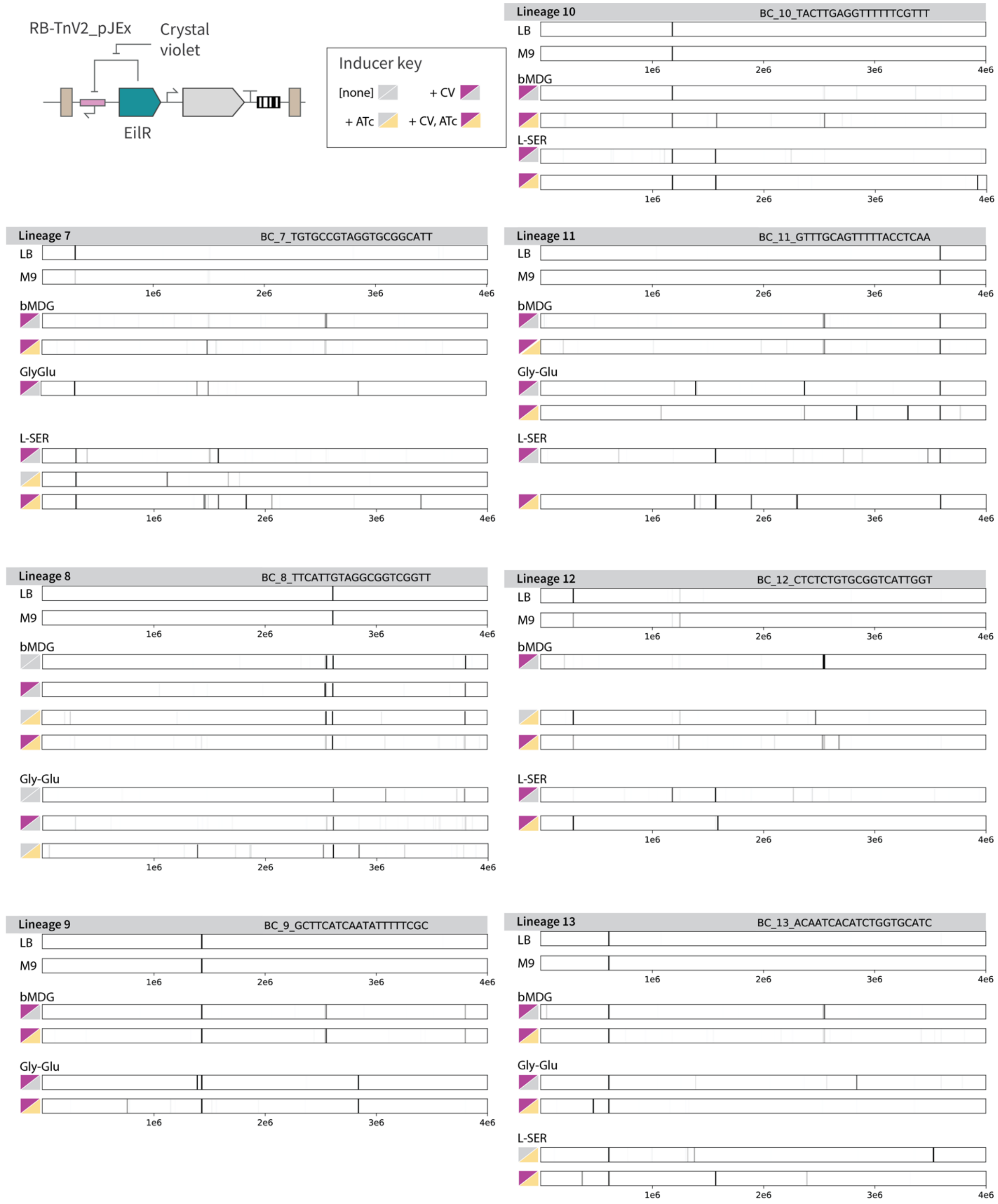

Appendix Figure S18. Heatmap plots for seven of the 14 unique RB-TnV2_pJex lineages showing the genomic locations of transposon insertions based on RB-Tn-Seq alignment data. For each different carbon source, the colored rectangles indicate the inducers added to each culture. L-SER, L-serine; bMDG, ß-methyl-D-glucoside; GlyGlu, glycl-L-glutamic acid. A detailed schematic of the experimental set-up is presented in Appendix Fig. S9. The heatmaps were generated as in Appendix Fig. S17.

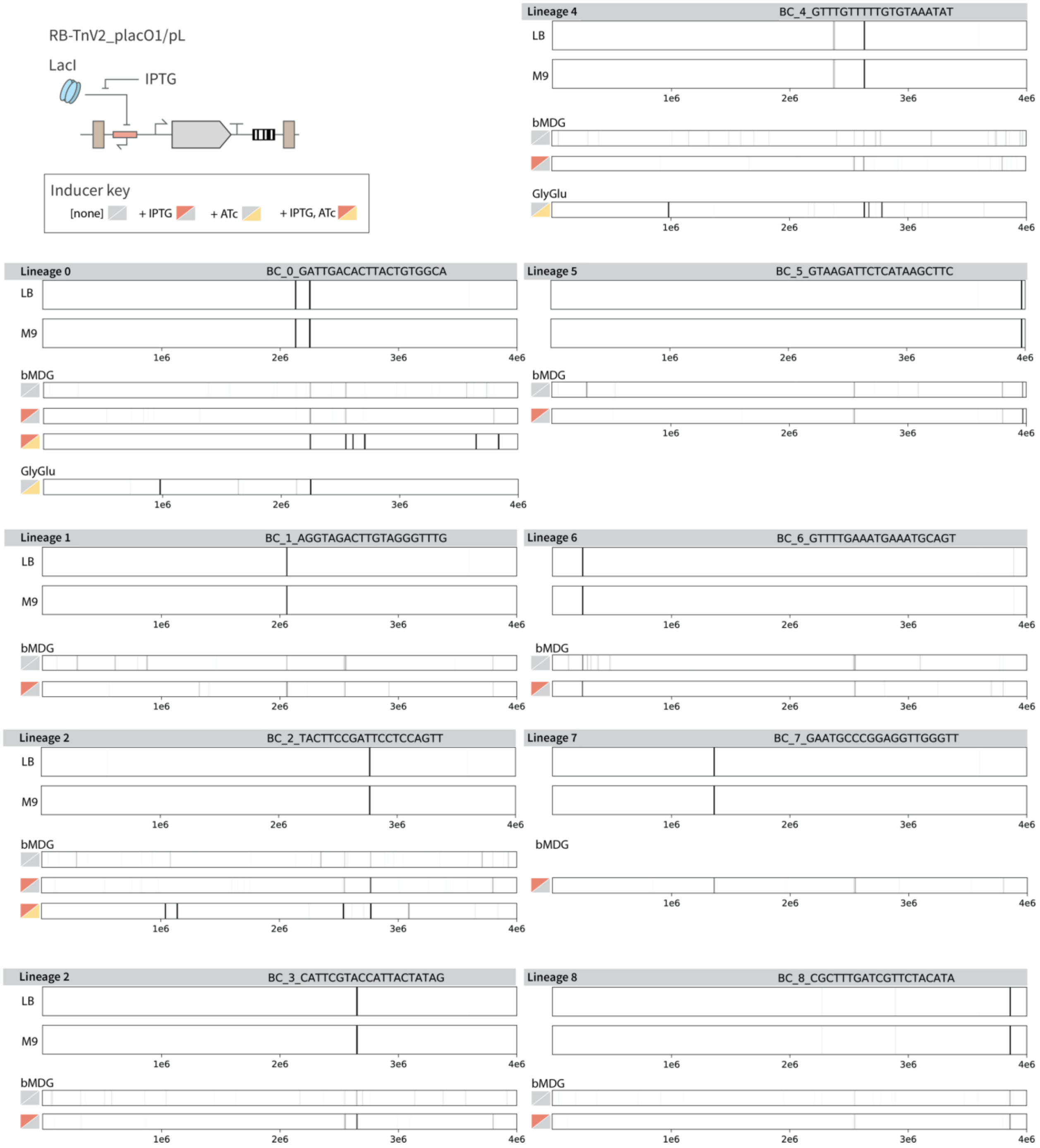

Appendix Figure S19. Heatmap plots for the nine unique RB-TnV2_pLacO1/pL lineages showing the genomic locations of transposon insertions based on RB-Tn-Seq alignment data. For each different carbon source, the colored rectangles indicate the inducers added to each culture. L-SER, L-serine; bMDG, ß-methyl-D-glucoside; GlyGlu, glycl-L-glutamic acid. A detailed schematic of the experimental set-up is presented in Appendix Fig. S9. The heatmaps were generated as in Appendix Fig. S17.

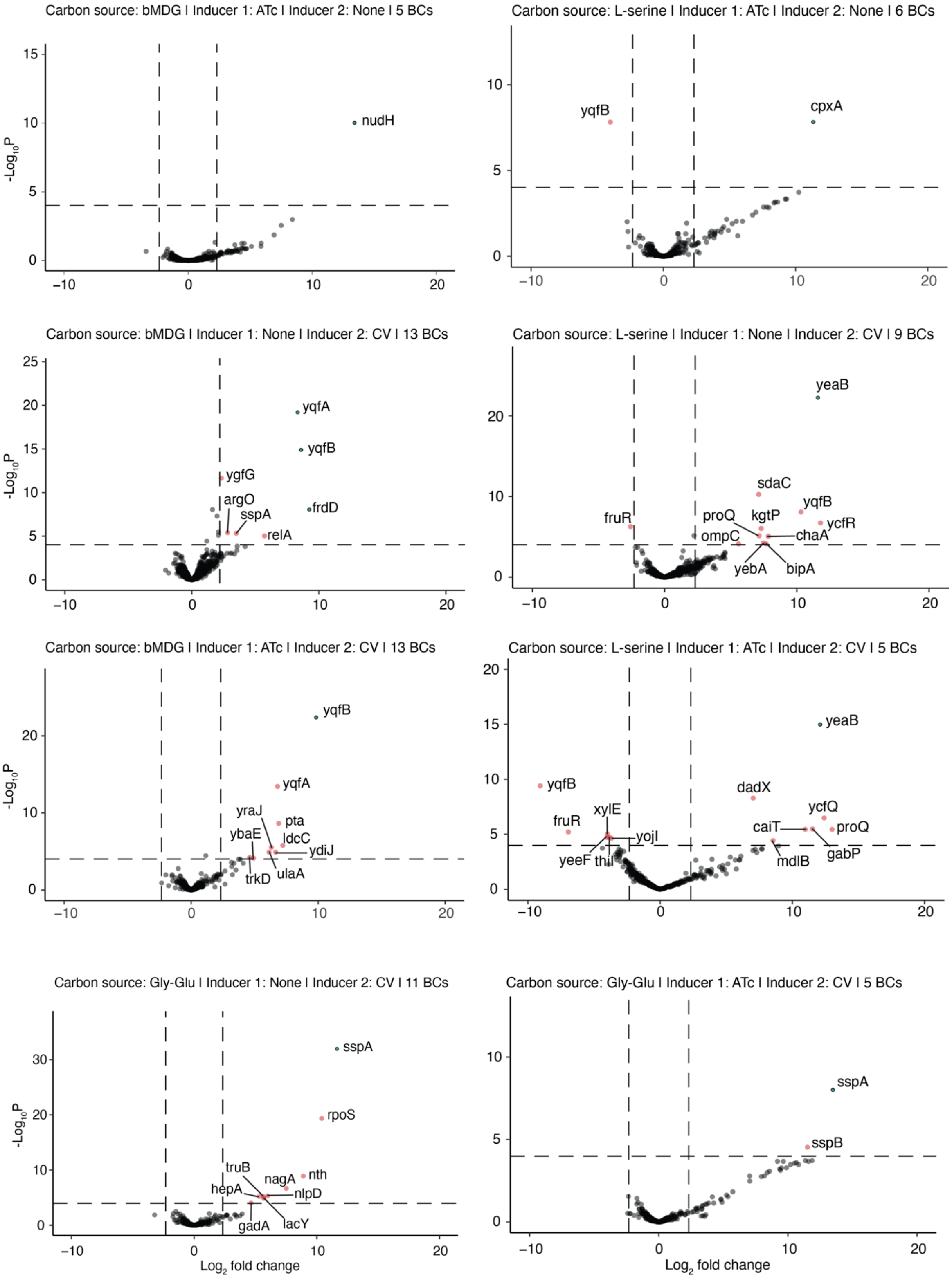

Appendix Figure S20. Volcano plots for all RB-TnV2_pJex lineages with n≥2 barcoded replicate (BCs). The points represent differentially enriched, annotated insertions sites between paired start-point (M9) and end-point samples (see plot titles for carbon source). Differential transposon enrichment analysis was performed as in Appendix Fig. S6

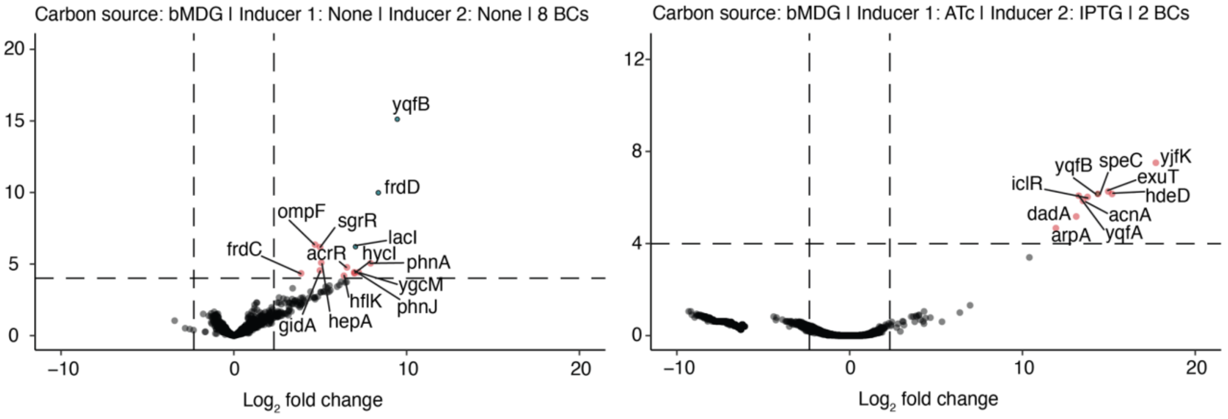

Appendix Figure S21. Volcano plots for all RB-TnV2_pLacO1/pL lineages with n≥2 barcoded replicate (BCs) not already shown in Fig. 4F. The points represent differentially enriched, annotated insertions sites between paired start-point (M9) and end-point samples (see plot titles for final carbon source). Differential transposon enrichment analysis was performed as in Appendix Fig. S6.

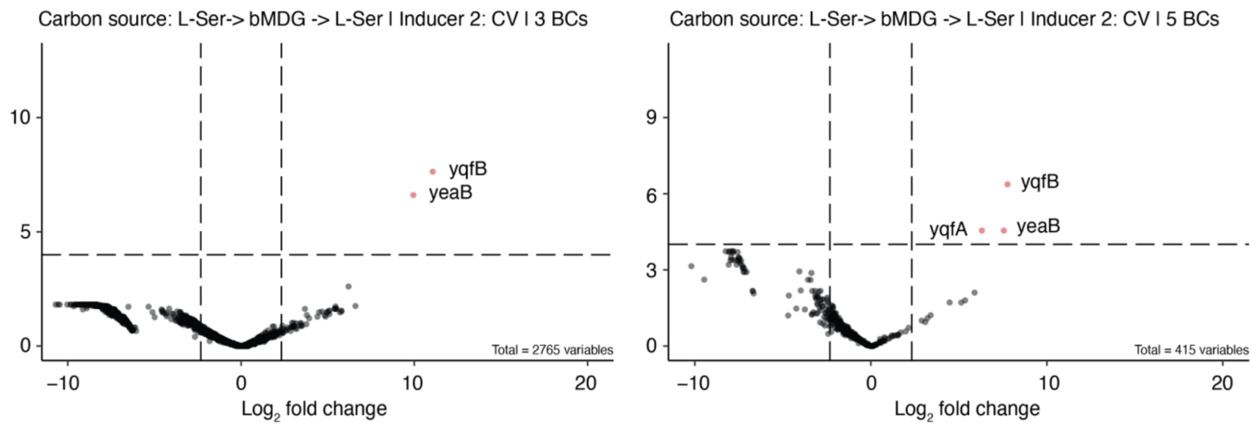

Appendix Figure S22. Volcano plots generated for two parallel replicate carbon source switching experiments described in Fig. 5A. In this experiment, RB-TnV2_pJex samples induced with crystal violet and either no ATc (left panel) or 50ng/ml ATc (right panel) were pre-evolved on L-serine and then secondarily evolved on ß-methyl-D-glucoside. For each replicate, unique RB-TnV2_pJex lineages were mapped to their founding colony in the initial evolution experiment using their established barcodes (Appendix Fig. S9). The points represent differentially enriched, annotated insertions sites between paired start-point (M9, from Fig. 4C and Appendix Fig. S17-18) and end-point samples (after growth on ß-methyl-D-glucoside re-growth on L-serine). Differential transposon enrichment analysis was performed as in Appendix Fig. S6.

Label	Pipeline	Index	Description	5' Mod.	Sequence	3' Mod.	Purification
sENG -020	Tn-seq	Nextera	Nextera adapter (top)	/Phos/	CTCACCGCTCTTG TAGSNNNNNNNNN CTGTCTCTTATAC ACATCTCCGAG*C	Phosphorothioated	HPLC
sENG -021	Tn-seq	Nextera	Nextera adapter (bottom)/TruSeq adapter (bottom)	None	CTACAAGAGCGG TGAGT	None	HPLC
sENG -022	Tn-seq	Nextera	Mariner transposon enrich (fwd) - custom modified for MmeI site tranposons	None	TCGTCGGCAGCG TCAGATGTGTAT AAGAGACAGNNN CCGGGGACTTAT CAtCCAAC*C	Phosphorothioated	HPLC
sENG -023	Tn-seq	Nextera	Mariner transposon enrich (rev) - adapter specific	None	GTCTCGTGGGCTC GGAGATGTGTAT AAGAGACA*G	Phosphorothioated	HPLC
sENG -024	Tn-seq	TruSeq	TruSeq adapter (top) - NOTE: ADDED AN "A" HERE AFTER THE UMI	/Phos/	CTCACCGCTCTTG TAGSNNNNNNNNN AGATCGGAAGAG CACACGTCT*G	Phosphorothioated	HPLC
sENG -025	Tn-seq	TruSeq	Mariner transposon enrich (fwd) - custom, using Mariner Tn binding site from sENG-022 (rather than the Tn5 one)	None	ACACTCTTTCCCT ACACGACGCTCT TCCGATCTNNNC CGGGGACTTATC AtCCAAC*C	Phosphorothioated	HPLC
sENG -026	Tn-seq	TruSeq	Mariner transposon enrich (fwd) - custom, using Mariner Tn binding site from sENG-015 (rather than the Tn5 one)	None	ACACTCTTTCCCT ACACGACGCTCT TCCGATCTNNNT GCGTTTCTACCTG CAGG*G	Phosphorothioated	HPLC
sENG -027	Tn-seq	TruSeq	Mariner transposon enrich (rev) - adapter specific	None	GTGACTGGAGTT CAGACGTGTGCT CTTCCGAT*C	Phosphorothioated	HPLC
sENG -028	Tn-seq	Nextera	Mariner transposon enrich (fwd) - original version with no MmeI binding site	None	TCGTCGGCAGCG TCAGATGTGTAT AAGAGACAGNNN CCGGGGACTTAT CAGCCAAC*C	Phosphorothioated	HPLC
sENG -029	Tn-seq	TruSeq	Mariner transposon enrich (fwd) - custom, using Mariner Tn binding site from sENG-028 (rather than the Tn5 one)	None	ACACTCTTTCCCT ACACGACGCTCT TCCGATCTNNNC CGGGGACTTATC AGCCAAC*C	Phosphorothioated	HPLC
sENG -030	RB-Tn-seq (two-step)	TruSeq	Binding site from Nspacer_barseq_universal, tail from TruSeq P5	None	ACACTCTTTCCCT ACACGACGCTCT TCCGATCTNNNN NNGATGTCCACG AGGTC*T	Phosphorothioated	HPLC
sENG -031	RB-Tn-seq (two-step)	TruSeq	Binding site from Nspacer_barseq_pHIMAR, tail from TruSeq P5	None	ACACTCTTTCCCT ACACGACGCTCT TCCGATCTNNNN NNCGCCCTGCAG GGATGTCCACGA *G	Phosphorothioated	HPLC
sENG -032	RB-Tn-seq (two-step)	Nextera	Binding site from Nspacer_barseq_universal, tail from sENG-022/028	None	TCGTCGGCAGCG TCAGATGTGTAT AAGAGACAGNNN NNNGATGTCCAC GAGGTC*T	Phosphorothioated	HPLC
sENG -033	RB-Tn-seq (two-step)	Nextera	Binding site from Nspacer_barseq_pHIMAR, tail from sENG-022/028	None	TCGTCGGCAGCG TCAGATGTGTAT AAGAGACAGNNN NNNCGCCCTGCA GGGATGTCCACG A*G	Phosphorothioated	HPLC

sENG -038	RB-Tn-seq (two-step)	TruSeq	MOD2_TruSeq, Y-adapter for ligation to genomic DNA fragments (1/2)	/Phos/	GATCGGAAGAGCACACGTCTGAACTCCAGTCA		HPLC
sENG -039	RB-Tn-seq (two-step)	TruSeq	Mod2_TS_Univ, Y-adapter for ligation to genomic DNA fragments (2/2)	None	ACGCTCTTCCGATC*T	Phosphorothioated	HPLC
sENG -040	RB-Tn-seq (two-step)	TruSeq	Mariner transposon enrich (rev) - adapter specific	None	GTGACTGGAGTTCAGACGTGTGCTCTTCCGATC*T	Phosphorothioated	HPLC
sENG -034	RB-Tn-seq (one-step)	TruSeq	Nspacer_barseq_universal, directly from RB-Tn-Seq paper with added A at 5'	None	AATGATACGGCGACCACCGAGATCTACACTCTTTCCCTACACGACGCTCTTCCGATCTNNNNNNGATGTCCACGAGGTC*T	Phosphorothioated	HPLC
sENG -035	RB-Tn-seq (one-step)	TruSeq	Nspacer_barseq_pHIMAR, directly from RB-Tn-Seq paper with added A at 5'	None	AATGATACGGCGACCACCGAGATCTACACTCTTTCCCTACACGACGCTCTTCCGATCTNNNNNNCGCCCTGCAGGGATGTCCACGA*G	Phosphorothioated	HPLC

Appendix Table S1. Oligonucleotide sequences for the NGS primers and adapters used in the Tn-Seq and RB-Tn-Seq sample preparation workflows. *, phosphorothioate linker; HPLC, High-performance liquid chromatography; /Phos/, 5’ phosphate modification.
