# Supplementary material for: A self‐propagating, barcoded transposon system for the dynamic rewiring of genomic networks
Source: Mol Syst Biol. 2023 Mar 27;19(6):e11398. doi: 10.15252/msb.202211398 (PMC10258560; doi:10.15252/msb.202211398)
Supplement: Supplementary file 11 [file MSB-19-e11398-s002.pdf]

molecular systems biology

A self-propagating, barcoded transposon system for the dynamic rewiring of genomic networks

Max English, Miguel Alcantar, and James Collins
DOI: 10.15252/msb.202211398

Corresponding author(s): James Collins (jimjc@mit.edu)

Review Timeline:

Submission Date:	13th Oct 22
Editorial Decision:	7th Nov 22
Revision Received:	4th Jan 23
Editorial Decision:	16th Feb 23
Revision Received:	8th Mar 23
Accepted:	10th Mar 23

Editor: Maria Polychronidou

Transaction Report:

(Note: With the exception of the correction of typographical or spelling errors that could be a source of ambiguity, letters and reports are not edited. Depending on transfer agreements, referee reports obtained elsewhere may or may not be included in this compilation. Referee reports are anonymous unless the Referee chooses to sign their reports.)

Thank you again for submitting your work to Molecular Systems Biology. We have now heard back from the three reviewers who agreed to evaluate your study. As you will see below, the reviewers appreciate that the study is relevant for the field. However, they raise a series of concerns, which we would ask you to address in a revision.

The recommendations of the reviewers are quite clear and I therefore see no need to repeat any of them here. In line with the reviewers' comments, we would ask you to revise the text for clarity and ensure that relevant details and information is included in the Results and Materials and Methods. All issues raised by the referees would need to be satisfactorily addressed. Please let me know in case you would like to discuss in further detail any of the issues raised, I would be happy to schedule a call.

Reviewer #1:

English and colleagues present a modular transposon-based toolkit for the continuous generation of engineered transposon insertions into the genome of E. coli. When coupled to selection/short-term evolution experiments, the authors demonstrate the results are (1) reproducible across replicate lineages and (2) recapitulate known biology in multiple instances, including the identification of genes and regulatory mechanisms responsible for arbutin catabolism and serine catabolism/resistance. To enable custom "functionalization" of their transposons with different parts (regulators, outward-facing promoters, antibiotic resistance cassettes, etc.), they adapted a Golden Gate assembly pipeline to streamline transposon vector construction. Lastly, they adapt random barcode transposon sequencing (RB-TnSeq), and demonstrate how the incorporation of unique DNA barcodes enables the parallel tracking of new transposon insertion events across multiple, independent evolving lineages. Overall, this work is an exciting extension of previous advances in transposon sequencing, and my impression is that it could be easily applied to additional bacterial hosts to uncover phenotypes achieved via multi-locus genetic modifications. And as the authors demonstrate, their system generates both loss-of-function (via disruption of the targeted gene) and gain-of-function mutations (via the constitutive or inducible outward facing promoter), enabling the exploration of a diverse phenotypic space.

Main points:
- Despite my enthusiasm for the work, the paper is challenging to read, mostly because the main body, in particular the Results section, leave out many details that are important for understanding the experiments and the figures. On the flip-side, the amount of detail in the figure legends, Methods section, and supplemental data is pretty overwhelming. My suggestions are to (1) make a separate Results section describing the carbon source fluctuation experiment (data presented in Figure 5) and (2) add more details about the experimental design and data interpretation in the Results section. Otherwise, the reader is going to have a difficult time following the methodology and the logic of each experiment.
- In the Discussion, it would be nice to mention some of the additional implications of this work, in particular how this method could be easily applied to new bacteria (including readily used strains for metabolic engineering like Pseudomonas putida), as well as how the use of DNA barcodes enables the pooling of many founder strains, and the parallel assessment of their evolutionary trajectory. I don't think this is explicitly stated and from my understanding, all experiments in this manuscript are derived from initial single founder colonies. Also, the modular design approach allows the addition of any genetic "cargo" into the transposon, including genes from non-host organisms (new regulators, catabolic enzymes, random DNA from a metagenome, etc.), which would be useful to mention.

Minor points:

- In the second paragraph of the Intro, last sentence. "As a result, many of the processes generating genome-wide structural heterogeneity in cells - for example, the duplication, translocation, transposition, or recombination of DNA segments - have yet to be widely adopted as methods for fitness landscape exploration during laboratory evolution." Is this true? Structural DNA variation is frequently observed in laboratory evolved lines of microbes such as E. coli in the absence of an engineering transposon system like the authors describe, and software packages like Breseq are specifically tailored for identifying this type of genetic variation.
- Per my point above, the data presented in Figure 1B is not talked about in the Results section.
- It's unclear if the data in Figure 1C is from MG1655 or MDS42 (the Results text says one, and the figure legend says the other).
- In Figure 1A(ii), should the network diagrams get flipped? You first perturb the network with transposons with outward facing promoters, and this leads to a re-wired network.
- In Figure 1A(iii), do the colors in the 96-well plate and at the top of the plot correspond to different conditions? So Scheme B represents an experiment that's similar to what's described in Figure 5?
- Some statistical significance for the data in Figure 1D would be nice to include.
- In the first results section, 3rd paragraph (the cycloserine section), the text mentions "following a severe bottleneck". Was this from a single colony, or a small population of Tn-containing cells?
- The assumption is that the cycloserine resistant populations in Figure 1D are the result of transposon insertions in cycA, but this isn't demonstrated. Are there other modes of E. coli resistance to cycloserine?

- Do the authors ever observe transposon insertions in DNA repair genes, leading to hypermutator phenotypes? This could complicate the interpretation of these lineages.
- Do the authors have a hypothesis about why no insertion mutants were identified in the operator region of bglG, similar to the natural mechanism via insertions from IS1 or IS5? Does this region contain TA sites?
- The authors don't present in the figures (or discuss) the orientation of the transposon insertions (plus versus minor strand). My assumption is that the outward facing promoter is always responsible for the gain of function expression of the downstream genes, but this should be explicitly stated in the text.
- In Figure 2A (and in some other figures), the Tn-pOUT cartoon is shown with a different color for the Tn IR with a promoter derived from this sequence. But the Tn IR is the same sequence for every transposon used in this study, correct? I recommend remaking the illustration with yellow for all of the IR ends, and a separate colored block for the outward facing promoter.
- What does the dotted line at OD = 0.10 mean in Figure 2B?

- In the modular designs with inducible outward-facing promoters, is the "cargo" the repressor gene? If yes, please state this.
- Figure 3B, it's hard to differentiate these lines. I recommend making a larger figure panel for this.
- In Figure 4DE, the lighter gray lines are hard to see.
- In Figure 5B, the purple and teal lines are hard to distinguish.
- A supplemental table(s) with the strains and plasmids used in the study should be included. Especially because interested readers are likely to request these materials.
- A careful reading of the Materials and Methods should be performed. For example, at least 3 different concentrations of chloramphenicol are reported. Is this accurate?
- Approximately how many DNA barcodes were introduced into the different Tn vectors? Some applications of the presented methods may

Reviewer #2:

English, Alcantar, and Collins describe the use of an engineered transposon system for inducing genomic changes. The paper introduced three goals: developing an assembly workflow to deliver transposon variants for gene network rewiring, evaluating the impact of the transposon variants on parallel evolving populations of E. coli, and using DNA barcodes to track transposon movement via longitudinal NGS. The paper demonstrated that the system successfully induces genomic changes by screening transposon-harboring strains against 31 different carbon sources, in which three carbon sources were identified where only the transposon-harboring strains evolved a growth advantage. NGS was used to identify insertions associated with gain-of-function or loss-of-function mutations enabling growth on different carbon sources. This work can be readily applied to laboratory evolution experiments, as the barcoded system allows lineages to be sequenced longitudinally in a high-throughput manner, and demultiplexed in silico. The barcode system has the additional advantage of being able to identify rare contamination events in plate-based assays. The transposon system also provides a mechanism to evolve organisms for growth on diverse feedstocks and could be used in industrial or biotechnological applications.
The authors have clearly done a large amount of work for this project, which I commend them for. In some cases, it borders on too much, making it difficult to follow. For instance, when multiple graphs are displayed to make the same point. Terminology can also be somewhat confusing due to the large number of "parts" they use in their transposon tools (one example noted below). Going through and simplifying/unifying terms would help the reader. With this exception, the paper is generally quite well written. The authors do a great job noting limitations and future directions of their work in the discussion.

Major 1: The terminology for the different transposon systems is somewhat confusing. I would come up with intuitive names for the them that describes their distinctions, and use those names consistently. Acronyms are hard to follow. For instance, In Fig 1. pBAD appears to be "TRANSPOSASE" in Fig 1Ai, pBAD-himar1C9 in Fig 1C, and pBAD in the final paragraph of "Constructing a self-propagating transposon mutagenesis platform", while "tetR-pTet pHelper" is referred to as "pHelper", "pTet-himar1C9", and "tetR-pTet pHelper" in the same. By using many different names, I am left wondering if they are the same things or if they are different. Actually, upon further reflection I see that I misunderstood. Fig 1C is showing which inducible system is best. This also helps to illustrate my point though, that the large number of terms used for similar things makes understanding what is being tested at each step quite confusing. The graph titles could be much more informative too. E.g. For Fig 1C, maybe "testing inducible Tnpase expression" would be a better title. For Fig 1D maybe "Testing mutagenicity of transposase systems". The axes labels could be better too. For instance, rather than "CFU/mL" it could be "CFU/mL on selective media" for Fig 1C, and "OD600 after 48h in D-cycloserine"

Minor 1: Typo "A major challenge in directed genome evolution is our limited ability to dynamically track the mutations changes across a genome, and mechanistically link these changes to the emergent phenotype19." Delete "changes"?
Minor 2: typo in SF2A: "suicicde"
Minor 3: For Fig 1B, if the point is to show that cells end up with both GFP and RFP, wouldn't it be better to show a scatter plot of RFP vs YFP? This is also minimally described in the paper.
Minor 4: In some cases, the OD appears to drop, but it is unclear why (e.g. Fig 4C). I assume this is due to dilution of the culture into the same or new media, but these dilution points are not always clear.
Minor 5: Unclear what Fig 2C and D add. "High-OD endpoint phenotypes (11/48 replicates) were only observed in the Tn-pOUT strain exposed to the ATc inducer (Fig. 2C)." Weren't all experiments in Fig 2C with ATc? Should this reference 2B? The text says "Based on this evidence for a transposon variant-specific growth phenotype (Fig. 2D), we modified an established Tn-Seq pipeline to compare the initial- and end-point insertion locations across all 11 replicates (Fig. 2E and Fig. S3)." I don't understand the reference to 2D means in this context.
Minor 6: "As expected, for the pre-selection populations each of the 11 replicates was identified with a unique, highly enriched 'founder' insertion (except in one case, where the initial insertion occurred near a multi-copy ribosomal gene) (Fig. S4)." Unclear why this was expected. Presumably because only one copy was needed before selection, and so the founders usually had a single copy?
Minor 7: It would be good to note early on that many of the gene names that are appearing as enriched are actually the same locus. E.g. sdaA/yeaB/pabB in Fig 3F. Also plots including Fig 2E, SF S4 (etc). It was not immediately apparent that these likely often represent a single transposition event given that they have multiple gene names.
Minor 8: It may be helpful to the reader to note how the authors anticipate this system being used for metabolic optimization. For instance, I would expect that you wouldn't want to use this system in the microbial end product, but rather as a way to discover which modifications to make, which are then made in a less genetically burdensome way (e.g. adding a promoter, rather than a promoter-harboring transposon).
Minor 9: Some of the fonts in the figures are very small and challenging to read (e.g. Fig 3B). It may help to print them out and gauge when printed on paper.
Minor 10: The section "Benchmarking the platform against a natural transposon-mediated adaptive switch" could use some clarification. E.g. "Guided by previous work developing this phenomenon into an assay for MGE activity14," is an important part that could be expanded on and seems to form the basis for the benchmark. It's then somewhat confusing that only the Tn-pOUT constructs are able to confer a selective advantage.
Minor 11: Many of the genomic insertion peak graphs have no Y-axis label (or even no Y axis). E.g. Fig 2G, 4GH.
Minor 12: Figure 1A, on the right hand side is somewhat confusing. I only figured it out after reading the whole paper (that the peaks in the Tn-seq are transposon insertion sites). Maybe label the transposon, the founder, the chromosome. Also, it is unclear where Scheme A was actually applied (in "Benchmarking the platform against a natural transposon-mediated adaptive switch"?). The L-Seriene experiment tested different concentrations after selection, but this was not a step-wise selection. The Scheme B comes in in Fig 5, and so could probably be saved for then. Might be best for Scheme A too to be in the appropriate section. Up to you though.

Minor 13: Consider clarifying what is meant in the discussion: "In this context, the advantage of our transposon platform lies in its apparent ability to introduce insertions at multiple locations in a genome through changes in the copy number of the transposon, mimicking established models for the creation of co-regulated gene networks through MGE dispersal3." For instance, is this referring to eukaryotic transposable elements, or are there also compelling examples from prokaryotes?
Minor 14: It is unclear what the simulation in Fig 3D adds vs Fig 3C. The EVOL-1 strain is clearly superior in L-Serine and has a dose-response relationship with concentration from Fig 3C.

Reviewer #3:

The paper by English et al investigates the use of a new transposon system that allows for multi-loci genomic rearrangements through continuous evolution. The authors show that using their two-plasmid system, they can not only create convergent strains that lead to improved growth advantages in non-conventional media, but using barcodes are able to track the lineage of these strains. For the most part, the results shown support the statements by the authors, but modifications to the manuscript will need to be made before this can be published. There is a lack of detail in the paper that makes it hard for the reader to follow all the experiments and will need to be significantly expanded. More details should be added in the results section to increase the readability of this paper. Furthermore, while I believe this approach could be important for the synthetic biology community, there is a lack of background and comparisons to previous research that really help explain the novelty of this research. It would be good to cross reference to research that has previously been done in the GRN evolution field.

Major points

Overall, there is a lack of discussion about what the results mean. For example, what does it mean that the p-Tet system is leaky (Figure 1C)? Should this not be used? If the authors do not want to add this discussion to the results the discussion section needs to be expanded to fully explain the benefits and weaknesses of the system so that other users may decide whether they wish to use this technology.

Despite reading the paper carefully, I particularly found the first two sections of the results chapters difficult to understand. By the third section the authors seem to have found a better way to explain the results and why each experiment was done and what it intended to show. As this paper shows a new technology, the initial experiments to set up the system should be made as simple as possible to understand so that as many people are possible will be able to utilize this technique.

It is unusual to have results in the materials and methods section that do not also appear in the main text of the paper (e.g., S2B and S2C) and particularly for the arbutin growth experiments the justifications that are included for the experiments in the material and methods are what I would expect to see in the rest of the main text. As a reader, it is difficult to flip back and forth between the materials and methods and the main text to understand the logic of the story.

The authors do not talk about whether the strains can be cured of the plasmid and the impact on the transposons being integrated into the genome can have on the stability of the strains over more generations. It would be of interest to see if, like with the lacI gene interruption, other beneficial modifications become apparent later. There is little information about what happens beyond 48 hours, do more mutations accumulate? Was there a reason the authors stopped at 48 hours?

I would be interested to know whether the same mutations that were found for the adaptation to different carbons sources have been used before, even by design or through random mutagenesis in previous adaptation experiments.

It's hard to follow what is happening in Figure 1B as the reference to it is only fleeting and the figure legend does not do enough to explain what is occurring.

For materials and methods discussing the arbutin assay, I feel that Figure S2B-E are results and should be in the main text and not in the materials and methods section.

Figure legend 1D, please comment on the statistics of this figure. What do the dotted lines mean vs the solid lines. In the text there is no comment on the distribution of the mutations on the colonies. For instance, in Tn, in the second transformation there appear to be a lot of colonies that barely grow, would these types of different distributions be expected?

Figure 4B, there is no discussion in the text about how the contamination occurs and what the impact of this might be. This is now referenced in regard to Fig S13 but further through the manuscript, but should be discussed upon first mention.

The figures in S1B and C are not discussed in the text and should be explained in the manuscript. Figure S1 was referred to in results section constructing a self-propagating transposon mutagenesis platform, paragraph 1, but this is not in relation to arbutin as far as I am aware and yet the figure legend specifically refers to this. Please either correct the figure legend or change where this figure is referenced in the manuscript.

Figure S2A, this figure is very confusing in the way that it is presented. What is the difference between the grey writing on the arrows and the grey writing at the bottom of the experiment. Are all the first steps consistent regardless of the strains and only the last growth in the selection +/- inducer different between the strains?

Figure S9, it is hard to understand the purpose of this experiment as it is not discussed in detail in the text.

Figure S16, why were no cells grown in the final passage, does this imply that the selection has not been as successful? Please expand on this in the main text.

Minor point

Please provide page numbers and line numbers to make it easier for reviewers to comment on specifics of the paper.

Figure 2A, please explain what site B is in the figure legend.

Materials and methods, under transformation and D-cyloserine assays, the authors are referring to Figure 1D and not 1C as written.

Manuscript ID: MSB-2022-11398
Title: A self-propagating, barcoded transposon system for the dynamic rewiring of genomic networks

Reviewer 1

General comments
English and colleagues present a modular transposon-based toolkit for the continuous generation of engineered transposon insertions into the genome of E. coli. When coupled to selection/short-term evolution experiments, the authors demonstrate the results are (1) reproducible across replicate lineages and (2) recapitulate known biology in multiple instances, including the identification of genes and regulatory mechanisms responsible for arbutin catabolism and serine catabolism/resistance. To enable custom "functionalization" of their transposons with different parts (regulators, outward-facing promoters, antibiotic resistance cassettes, etc.), they adapted a Golden Gate assembly pipeline to streamline transposon vector construction. Lastly, they adapt random barcode transposon sequencing (RB-TnSeq), and demonstrate how the incorporation of unique DNA barcodes enables the parallel tracking of new transposon insertion events across multiple, independent evolving lineages. Overall, this work is an exciting extension of previous advances in transposon sequencing, and my impression is that it could be easily applied to additional bacterial hosts to uncover phenotypes achieved via multi-locus genetic modifications. And as the authors demonstrate, their system generates both loss-of-function (via disruption of the targeted gene) and gain-of-function mutations (via the constitutive or inducible outward facing promoter), enabling the exploration of a diverse phenotypic space.

Main points
Reviewer 1 Comment 1
Despite my enthusiasm for the work, the paper is challenging to read, mostly because the main body, in particular the Results section, leave out many details that are important for understanding the experiments and the figures. On the flip-side, the amount of detail in the figure legends, Methods section, and supplemental data is pretty overwhelming. My suggestions are to (1) make a separate Results section describing the carbon source fluctuation experiment (data presented in Figure 5) and (2) add more details about the experimental design and data interpretation in the Results section. Otherwise, the reader is going to have a difficult time following the methodology and the logic of each experiment.

Response:
We thank the reviewer for their constructive comments. We agree that additional discussion of the results and experimental design would improve the clarity of the manuscript. In line with the reviewer's suggestions, we have implemented the following changes to the main text:

1. We have added an additional paragraph to the first results section that elaborates on how the transposon platform can facilitate directed evolution in bacterial genomes and the general workflow used to validate the platform in all subsequent experiments (lines 137-145).
2. We have expanded the results section discussing Figure 4 to elaborate on the modular transposon assembly and barcoding approach (lines 250-263).
3. We have created a new section for the results presented in Figure 5. This section includes additional discussion about the carbon source fluctuation experiment and explicitly discusses important observations (lines 330-351).

We believe that these modifications, alongside the other changes suggested by the reviewer, have improved the clarity of our manuscript.

Reviewer 1 Comment 2
In the Discussion, it would be nice to mention some of the additional implications of this work, in particular how this method could be easily applied to new bacteria (including readily used strains for metabolic engineering like Pseudomonas putida), as well as how the use of DNA barcodes enables the pooling of many founder strains, and the parallel assessment of their evolutionary trajectory. I don't think this is explicitly stated and from my understanding, all experiments in this manuscript are derived from initial single founder colonies. Also, the modular design approach allows the addition of any genetic "cargo" into the transposon, including genes from non-host organisms (new regulators, catabolic enzymes, random DNA from a metagenome, etc.), which would be useful to mention.

Response:
We thank the reviewer for these valuable suggestions. We have incorporated additional text in the Discussion to describe how this platform could be expanded to different bacterial species, and how a different pooled experiment could be conducted with the same barcoding strategy (lines 390-412). We have also added additional details about how cargos could be incorporated and the Golden Gate flanking sequences that should be used (Table 3).

Minor points
Reviewer 1 Comment 3
In the second paragraph of the Intro, last sentence. "As a result, many of the processes generating genome-wide structural heterogeneity in cells - for example, the duplication, translocation, transposition, or recombination of DNA segments - have yet to be widely adopted as methods for fitness landscape exploration during laboratory evolution." Is this true? Structural DNA variation is frequently observed in laboratory evolved lines of microbes such as E. coli in the absence of an engineering transposon system like the authors describe, and software packages like Breseq are specifically tailored for identifying this type of genetic variation.

Response:
We thank the reviewer for making this important point. We agree that these biological processes can play major roles in natural evolution experiments. However, our intention was to highlight that these processes have yet to be widely adapted into engineered platforms to facilitate fitness landscape exploration. We have modified the main text to clarify this point (lines 49-50).

Additionally, we note that while *E. coli* MDS42 does not contain insertion sequence elements, it is still capable of undergoing unrelated genome-wide structural rearrangements. Despite this, we typically observe that the parental MDS42 strain which lacks our engineered transposon platform is unable to develop adaptive mutations in the timescale of our experiments. This indicates that the adaptive mutations observed in our experiments are introduced specifically through transposon-mediated mutagenesis.

Reviewer 1 Comment 4
Per my point above, the data presented in Figure 1B is not talked about in the Results section.

Response:
We have added additional text elaborating on the results shown in Figure 1B and their implications for our engineered platform (lines 101-111). In line with Reviewer 2's Comment 4, we have also modified the title for Figure 1B to more clearly emphasize the main conclusion drawn from this experiment.

Reviewer 1 Comment 5
It's unclear if the data in Figure 1C is from MG1655 or MDS42 (the Results text says one, and the figure legend says the other).

Response:
We thank the reviewer for pointing out this inconsistency. The experiments in Figure 1C were conducted in *E. coli* MDS42, but were erroneously described as *E. coli* MG1655 in the main text. We have now corrected all erroneous mentions of *E. coli* MG1655.

Reviewer 1 Comment 6
In Figure 1A(ii), should the network diagrams get flipped? You first perturb the network with transposons with outward facing promoters, and this leads to a re-wired network.

Response:
We thank the reviewer for this suggestion. We have now flipped the diagrams to better emphasize that the network rewiring occurs after transposon-mediated perturbations to the host genome.

Reviewer 1 Comment 7
In Figure 1A(iii), do the colors in the 96-well plate and at the top of the plot correspond to different conditions? So Scheme B represents an experiment that's similar to what's described in Figure 5?

Response:
We agree that the original color scheme was confusing. We have simplified the color scheme to more accurately reflect the general layout of our evolution experiments and emphasize the following points:

1. Each experiment has multiple replicates for each population condition. These replicates are arrayed in adjacent wells of a 96 well plate.
2. We include transposase-only controls (abbreviated as "Tnpase" in the figures) as well as parental strains lacking both transposon and transposase.
3. Each experiment includes conditions with and without the transposase inducer (e.g., ATc).

In addition, we have labeled schemes A and B with the figures that use these respective selection regimes.

Reviewer 1 Comment 8
Some statistical significance for the data in Figure 1D would be nice to include.

Response:

We have added statistical comparisons for the data presented in Figure 1D and have updated the corresponding figure caption (lines 447-448) as well as the Methods section (lines 676-679).

Reviewer 1 Comment 9
In the first results section, 3rd paragraph (the cycloserine section), the text mentions "following a severe bottleneck". Was this from a single colony, or a small population of Tn-containing cells?

Response:
The bottleneck was enforced through a 10,000-fold dilution when transferring cells from non-selective minimal media to the selective, D-cycloserine containing media. We have modified the text to include this important detail (line 129).

Reviewer 1 Comment 10
The assumption is that the cycloserine resistant populations in Figure 1D are the result of transposon insertions in cycA, but this isn't demonstrated. Are there other modes of E. coli resistance to cycloserine?

Response:
We thank the reviewer for this comment. The D-cycloserine resistance fluctuation assay is a well-established method that uses the spontaneous inactivation of the *cycA* gene to estimate genomic mutation rates. For *E. coli* grown in minimal media, CycA is the transporter primarily responsible for the uptake of D-cycloserine[1]. As a result, resistance to D-cycloserine emerges almost exclusively from loss-of-function mutations in the *cycA* gene (e.g., point mutations, deletions, and insertions)[2,3]. While there exist additional mechanisms that confer resistance to D-cycloserine, such as the disruption of the *dadA* gene which encodes a D-amino acid dehydrogenase, we do not expect these to be prominent in our experimental conditions.

As noted above, transposon insertion is not the sole mechanism contributing to *cycA* inactivation. Wild type *E. coli* MDS42 does not contain insertion elements but can develop resistance to D-cycloserine via the emergence of point mutations and deletions in the *cycA* coding region. Nonetheless, the presence of additional *cycA*-disrupting mutations does not change our interpretation of the results presented in Figure 1D: the introduction of an inducible transposon-transposase system provides a means to increase transposon-mediated genomic mutation rates. We have modified the text to clarify this interpretation (lines 134-135). In addition, we have qualified our description of the assay by mentioning that spontaneous inactivation of *cycA* is the “typical” mode of *E. coli* resistance to D-cycloserine (line 125).

Reviewer 1 Comment 11
Do the authors ever observe transposon insertions in DNA repair genes, leading to hypermutator phenotypes? This could complicate the interpretation of these lineages.

Response:
We thank the reviewer for raising this point. We reanalyzed our sequencing data and comprehensively attempted to identify samples that contained either founder insertions or highly enriched endpoint insertions near genes associated with DNA damage repair pathways[4]. We did not identify any founder insertions in repair genes, nor did any repair gene emerge as statistically enriched in endpoint populations versus starting populations. We did identify one endpoint sample (carbon source: bMDG; plasmid: RB-TnV2_placO1/pL; inducer: no IPTG; barcode id: 1) from the

random barcode evolution experiment presented in Figure 4 which contained a highly enriched insertion in the *sulA* gene, presumably inactivating the gene. The *sulA* gene is normally involved with inducing cell lysis in the presence of irreparable DNA damage. To our knowledge, inactivation of *sulA* does not directly lead to a hypermutator phenotype. This analysis helps confirm that direct activation or inactivation of genes is the most prevalent mode of adaptation with the engineered transposon system rather than the emergence of hypermutator phenotypes.

One possible reason we commonly observed the direct activation or inactivation of a small set of genes in our evolution experiments is that the phenotypes we were selecting for are binary with respect to growth. More specifically, there generally exist well-defined gain-of-function or loss-of-function mutations that yield growth-positive phenotypes. In contrast, selection in conditions that require many mutations to yield a beneficial phenotype could potentially increase the prevalence of transposon insertions that induce hypermutator phenotypes.

Reviewer 1 Comment 12
Do the authors have a hypothesis about why no insertion mutants were identified in the operator region of bglG, similar to the natural mechanism via insertions from IS1 or IS5? Does this region contain TA sites?

Response:
We thank the reviewer for this comment. The operator region upstream of *bglG* does contain TA sites. However, there are two related explanations why we did not observe insertions within this region in the experiment presented in Figure 2.

First, the direct upregulation of *bglF/B/H* likely results in faster adaptation to arbutin compared to insertional mutations upstream of *bglG*. In a preliminary experiment, we observed that *E. coli MDS42* cells which contained an autonomous transposon required at least 96 hours to grow in media that contained arbutin as the sole carbon source (Appendix Figure S1). Since these transposons did not contain an outward-facing promoter, the most likely routes towards activation of the *bgl* operon were either insertional mutations upstream of *bglG* or disruption of the *hns* gene which encodes the transcriptional regulator that represses *bglG* expression. Inactivation of *hns* is known to be a rare adaptation[5]. Therefore, growth in this preliminary experiment likely occurred through insertional mutations upstream of *bglG*. In contrast, the evolution experiment presented in Figure 2 involved a more gradual increase in arbutin levels and a shorter, 48-hour incubation in media containing arbutin as the sole carbon source. While the evolution scheme and transposon architecture differed between the preliminary experiment and the experiment in Figure 2, we believe these results lend support to the possibility that 48 hours was not sufficient for *E. coli* to develop arbutin-catabolizing adaptations via insertional mutations upstream of *bglG* and instead preferentially upregulated the expression of genes downstream of *bglG*. It is possible that with longer incubation times we may also observe insertional mutations upstream of *bglG*.

Second, the outward-facing promoter harbored on our transposon may not effectively overcome repression of an additional H-NS binding site located within *bglG*. The natural mechanism for *bgl* operon activation typically involves two related events: (i) insertion sequence-mediated disruption of repressive operator sites upstream of the *bglG* promoter, and (ii) enhancement of *bglG* promoter activity by trans-activating elements harbored on some insertion sequences, such as IS5[6]. These two mechanisms lead to a 40-60-fold increase in *bglG* promoter activity which is sufficient to overcome repression by an additional H-NS operator binding site located within the *bglG* coding region[6,7]. In

cases where insertions occur upstream of *bglG*, the outward-facing promoter harbored on our transposon may not overcome repression of the intragenic H-NS operator binding site which leads to slow accumulation of *bglG* and *bgl* activation. In this case, we would not recapitulate the natural mechanism for *bgl* operon activation in the 48-hour evolution window and instead observe direct activation of genes downstream of *bglG*, which do not contain internal operator sites.

We have added text to the results section in order to elaborate on why we observed a distinct mechanism leading to activation of the *bgl* operon (lines 187-191).

Reviewer 1 Comment 13

The authors don't present in the figures (or discuss) the orientation of the transposon insertions (plus versus minor strand). My assumption is that the outward facing promoter is always responsible for the gain of function expression of the downstream genes, but this should be explicitly stated in the text.

Response:

We agree that additional discussion on the orientation of transposon insertions is warranted and would improve the interpretability of the alignment plots (e.g., Figures 2G and 3G). In general, the only requirement for transposon insertion is a TA dinucleotide. If one DNA strand contains a TA site, the other strand must also contain a TA site due to DNA complementarity. This means that for any given TA site, the transposon can theoretically insert in either orientation.

For all experiments with the non-barcoded transposon variants (i.e., Figures 2 and 3; the Tn-Seq experiments), both the forward and reverse sequencing reads consist entirely of gDNA-derived sequences as the primer anneals at the extreme end of the transposon sequence. Therefore, it is not possible to determine the orientation of transposon insertion solely from the alignment plots. Instead, the orientation can be predicted with high confidence based on prior knowledge of the biological mechanism required to yield a growth phenotype. As an example, *E. coli* are only able to catabolize arbutin after activation of the *bgl* operon. We can therefore assume that Tn-pOUT is inserted such that the outward-facing promoter is driving expression in the same direction as the *bglF/B/H* coding sequences. Similarly, we assume that Tn-pOUT is inserted such that the outward-facing promoter drives expression of *sdaA*, which is required for L-serine catabolism. In these contexts, the transposon lacking an outward-facing promoter represents an important negative control to support the validity of this assumption: it is otherwise identical to Tn-pOUT, and so if the mechanisms did not specifically require the promoter we would expect a broadly similar number of growth-positive replicates in this condition.

Conversely, for all experiments with the barcoded transposon variants (i.e., Figures 4 and 5; the RB-Tn-Seq experiments) it is possible to determine the transposon orientation based on the shape of the alignment plots. This represents an additional reason why we decided to progress to this new workflow. In a typical RB-Tn-Seq sequence fragment, the forward read contains the inverted repeat sequence plus the DNA barcode, and only the reverse read consists of gDNA-derived sequences which align to the host genome. As a result, the shape of the peak plots is asymmetrical and the alignments accumulate in the opposite direction of the outward-facing promoter. For example, in Figure 4G the alignments accumulate away from *bglA*. Since the outward-facing promoter is oriented such that it is on the opposite end with respect to the DNA barcode and sequencing reads (see Figure 4B for the transposon architectures) we can conclude that the promoter is driving expression in the same direction as the *bglA* coding sequence.

To make the transposon orientation less ambiguous, we have modified the operon schematics such that genes that are presumably activated by outward-facing promoters are now colored orange. We have also included additional text in the results section (lines 286-287) and the Figure 4G caption (lines 539-541) to describe how the transposon orientation is deduced for RB-Tn-Seq experiments.

Reviewer 1 Comment 14

In Figure 2A (and in some other figures), the Tn-pOUT cartoon is shown with a different color for the Tn IR with a promoter derived from this sequence. But the Tn IR is the same sequence for every transposon used in this study, correct? I recommend remaking the illustration with yellow for all of the IR ends, and a separate colored block for the outward facing promoter.

Response:

We thank the reviewer for pointing out this inconsistency. In this study, we used two different transposon inverted repeat variants. The first variant was harbored on both the original Tn and Tn-pOUT transposons. The second variant was harbored on all the barcoded transposons (RB-TnV2, RB-TnV2_pJEx and RB-TnV2_placO1/pL). We have standardized our color scheme such that: (i) the non-barcoded transposon inverted repeat variant is colored yellow, and (ii) the barcoded transposon inverted repeat variant is colored brown.

Additionally, we have modified all transposon schematics such that the outward-facing promoters are on a separate, colored block.

Reviewer 1 Comment 15

What does the dotted line at OD = 0.10 mean in Figure 2B?

Response:

In this experiment, we used OD600 $\geq$ 0.10 as a working definition of the growth-positive phenotype on arbutin. As such, the individual populations that exceeded this endpoint OD600 (n=11) were the ones submitted for individual sequencing. We have now labeled the dotted line in Figure 2B with this description and modified the title for Figure 2F to specify that only these 11 growth-positive populations were sequenced.

Reviewer 1 Comment 16

In the modular designs with inducible outward-facing promoters, is the "cargo" the repressor gene? If yes, please state this.

Response:

We agree with the reviewer that this point warrants additional clarification. In these examples, we do not include a cargo element: the repressor is in fact included within the promoter part. The cargo promoter and cargo are replaced with a non-coding “spacer” part that spans both elements. The cargo promoter and cargo parts are included to make this system as flexible as possible for any end users: for example, they could be used to encode reporters and their constitutive or inducible promoters, or additional genetic regulators such as transcription factors to facilitate more complex gene network rewiring.

We have made changes to the text to clarify this point (lines 258-263), and have included an index of all parts used in this study and their respective sequences (Table 3).

Reviewer 1 Comment 17
Figure 3B, it's hard to differentiate these lines. I recommend making a larger figure panel for this.

Response:
We agree with the reviewer that this panel is too small. We have removed the redundant panel Figure 3D (in line with Reviewer 2's Comments 10 and 15), and have expanded the two graphs to make the labeling clearer.

Reviewer 1 Comment 18
In Figure 4DE, the lighter gray lines are hard to see.

Response:
We agree with the reviewer that in some cases the fainter gray lines are challenging to see, particularly in print. The shading here represents a quantitative color scale and so less enriched peaks will have a lower color intensity. To improve the ease of interpretation, we have added the color scale to the graph, increased the line widths by changing the bin sizes from 10,000bp to 20,000bp, and lowered the maximum threshold cutoff for the color scaling such that less enriched peaks appear darker.

Reviewer 1 Comment 19
In Figure 5B, the purple and teal lines are hard to distinguish.

Response:
We have increased the intensity of the teal color, and agree with the reviewer that this improves the legibility of the figure.

Reviewer 1 Comment 20
A supplemental table(s) with the strains and plasmids used in the study should be included. Especially because interested readers are likely to request these materials.

Response:
We thank the reviewer for this constructive suggestion. We have included tables with strains, plasmids, and DNA parts (Reagents and Tools Table; Dataset EV1).

Reviewer 1 Comment 21
A careful reading of the Materials and Methods should be performed. For example, at least 3 different concentrations of chloramphenicol are reported. Is this accurate?

Response:
We thank the reviewer for their comment. We have carefully reviewed the Methods section and confirm that the multiple chloramphenicol concentrations reported in the Methods are correct. We changed the concentration of chloramphenicol used depending on the initial population size and the volume of media used for evolution experiments. For example, in experiments conducted in Biolog plates (Figure 3) we used small sample values (100µL) which were inoculated with small bacterial population sizes. Hence, we lowered the concentration of chloramphenicol used in order to prevent cells from being overburdened from high antibiotic concentrations.

Reviewer 1 Comment 22
Approximately how many DNA barcodes were introduced into the different Tn vectors? Some applications of the presented methods may

Response:
We thank the reviewer for raising this important concern. For our experiments using barcoded transposon variants, we used an N20 barcode as described in the original RB-Tn-Seq paper. Hence, we expect the number of barcodes introduced through cloning to be orders of magnitude above the number of founder colonies we would pick in a typical experiment. This would make the likelihood of the same N20 barcode sequence appearing in different founder colonies extremely low.

Unfortunately, the second part of the reviewer's comment was cut off, but we assume they were interested in applications where a much larger number of founder colonies is used (e.g., starting from a library of 1000s of initial insertions). This is the exact use case for which the RB-Tn-Seq method was originally developed, as they were using saturated mutagenesis libraries. In this case, it would be important to deep sequence the original barcode library after cloning to verify that there was sufficient barcode diversity. In addition, if multiple different transposon architectures are used, it is also possible to include predefined flanking sequences unique to each backbone or transposon architecture to further delineate between lineages. The only sequence requirements for the barcode insert are SbfI-FseI restriction enzyme-compatible overhangs which are used for cloning.

Reviewer 2

General comments
English, Alcantar, and Collins describe the use of an engineered transposon system for inducing genomic changes. The paper introduced three goals: developing an assembly workflow to deliver transposon variants for gene network rewiring, evaluating the impact of the transposon variants on parallel evolving populations of E. coli, and using DNA barcodes to track transposon movement via longitudinal NGS. The paper demonstrated that the system successfully induces genomic changes by screening transposon-harboring strains against 31 different carbon sources, in which three carbon sources were identified where only the transposon-harboring strains evolved a growth advantage. NGS was used to identify insertions associated with gain-of-function or loss-of-function mutations enabling growth on different carbon sources. This work can be readily applied to laboratory evolution experiments, as the barcoded system allows lineages to be sequenced longitudinally in a high-throughput manner, and demultiplexed in silico. The barcode system has the additional advantage of being able to identify rare contamination events in plate-based assays. The transposon system also provides a mechanism to evolve organisms for growth on diverse feedstocks and could be used in industrial or biotechnological applications.

The authors have clearly done a large amount of work for this project, which I commend them for. In some cases, it borders on too much, making it difficult to follow. For instance, when multiple graphs are displayed to make the same point. Terminology can also be somewhat confusing due to the large number of "parts" they use in their transposon tools (one example noted below). Going through and simplifying/unifying terms would help the reader. With this exception, the paper is

generally quite well written. The authors do a great job noting limitations and future directions of their work in the discussion.

Main points
Reviewer 2 Comment 1
The terminology for the different transposon systems is somewhat confusing. I would come up with intuitive names for the them that describes their distinctions, and use those names consistently. Acronyms are hard to follow. For instance, In Fig 1. pBAD appears to be "TRANSPOSASE" in Fig 1Ai, pBAD-himar1C9 in Fig 1C, and pBAD in the final paragraph of "Constructing a self-propagating transposon mutagenesis platform", while "tetR-pTet pHelper" is referred to as "pHelper", "pTet-himar1C9", and "tetR-pTet pHelper" in the same. By using many different names, I am left wondering if they are the same things or if they are different. Actually, upon further reflection I see that I misunderstood. Fig 1C is showing which inducible system is best. This also helps to illustrate my point though, that the large number of terms used for similar things makes understanding what is being tested at each step quite confusing. The graph titles could be much more informative too. E.g. For Fig 1C, maybe "testing inducible Tnpase expression" would be a better title. For Fig 1D maybe "Testing mutagenicity of transposase systems". The axes labels could be better too. For instance, rather than "CFU/mL" it could be "CFU/mL on selective media" for Fig 1C, and "OD600 after 48h in D-cycloserine"

Response:
We thank the reviewer for their constructive comments. We agree that our inconsistent use of abbreviations for the helper plasmids is confusing. We have consolidated the nomenclature for the two inducible transposase systems into two acronyms:

1. The arabinose-inducible transposase system is now named “AraC/pBAD-Tnpase”.
2. The ATc-inducible transposase system is now named “TetR/pTet-Tnpase”.

In addition, we have included supplementary tables with descriptions for all plasmids used in this study, including the figures in which they appear (Table 1 and Dataset EV1).

Lastly, we have modified the figure titles and axes labels in Figures 1B-D to be more descriptive, and agree that this improves the interpretability of these plots.

Minor points
Reviewer 2 Comment 2
Typo "A major challenge in directed genome evolution is our limited ability to dynamically track the mutations changes across a genome, and mechanistically link these changes to the emergent phenotype19." Delete "changes"?

Response:
We have corrected this typo (line 47), and thank the reviewer for the suggested change.

Reviewer 2 Comment 3
typo in SF2A: "suicicde"

Response:
We have corrected this typo in Appendix Fig. S2, and thank the reviewer for the suggested change.

Reviewer 2 Comment 4
For Fig 1B, if the point is to show that cells end up with both GFP and RFP, wouldn't it be better to show a scatter plot of RFP vs YFP? This is also minimally described in the paper.

Response:
We thank the reviewer for their suggestion. We created a scatter plot of RFP vs. GFP fluorescence and found this visualization less interpretable than our original histograms. Additionally, in this case we believe it is more important to highlight the RFP- and GFP-labeled transposon populations that arise from random genomic integrations as opposed to individual cells, which is why we elected to display the red and green channels as separate plots.

Nonetheless, we appreciate the reviewer's comment, as it points out that the purpose of the experiment and conclusions drawn from the results were not clearly explained. To address this, we have included additional text in the results section to elaborate on our interpretation of Figure 1B (lines 101-111) and we have included a more descriptive title for Figure 1B.

Reviewer 2 Comment 5
In some cases, the OD appears to drop, but it is unclear why (e.g. Fig 4C). I assume this is due to dilution of the culture into the same or new media, but these dilution points are not always clear.

Response:
The reviewer raises an important observation that is common across these plate based-assays. In the case of the results in Figure 2D, we observe gradual decreases in OD600 in the arbutin evolution experiments after the cells enter stationary phase, likely due to cell death. Arbutin itself is fairly toxic for the cells, and the very low nutrient levels in this minimal media result in cell lysis during long-term incubation.

In the experiments presented in Figure 4 and the associated supplementary appendix figures, the observed decreases in OD600 levels are an artifact of the low temporal resolution of the measurement and the "one-size-fits-all" approach to passaging. Early-arising growth phenotypes will remain in stationary phase for longer before the subsequent passage. In some cases, this can actually mean a (much) lower viable inoculum than for late-arising growth phenotypes. Furthermore, the OD600 for rapidly growing cultures may have peaked and begun to decrease again before the next OD600 measurement was taken (e.g., in experiments when OD600 values were measured only after 48 hrs from the previous passage). This issue is inherent to the fact that multiple different transposon architectures, inducer conditions and their respective controls were growing in the same 96-well plate. We were not able to adjust the passaging times for each condition in isolation. The development of automated, high-throughput liquid handling platforms (e.g., the PRANCE or eVOLVER platforms) that can adjust dilution rates in real time on a per-well/per-variant basis could alleviate this issue as they become more widely adopted.

Reviewer 2 Comment 6
Unclear what Fig 2C and D add. "High-OD endpoint phenotypes (11/48 replicates) were only observed in the Tn-pOUT strain exposed to the ATc inducer (Fig. 2C)." Weren't all experiments in Fig 2C with ATc? Should this reference 2B? The text says "Based on this evidence for a transposon variant-specific growth phenotype (Fig. 2D), we modified an established Tn-Seq pipeline to

compare the initial- and end-point insertion locations across all 11 replicates (Fig. 2E and Fig. S3)." I don't understand the reference to 2D means in this context.

Response:
We thank the reviewer for their constructive comments. Since Figure 2B only shows the endpoint OD600 values for each population after 48hrs in arbutin, we included Figure 2C and 2D to highlight the following points:

1. Figure 2C is meant to highlight the longitudinal experiment structure with increasing concentrations of arbutin and to help visualize the different growth phenotypes (e.g., early- vs. late-arising growth phenotypes). We focused solely on a single condition for this plot, namely the Tn-pOUT transposon variant with ATc added to the media.
2. Figure 2D is meant to showcase the growth profiles in arbutin for growth-positive populations (highlighted in orange) compared to those that do not meet our definition of growth positive (gray), in particular the biphasic growth phases of these variants.

Regarding the reviewer's second point, as shown in Figure 2B each strain was cultured in conditions with and without ATc. In Figure 2C, we elected to plot only the +ATc condition as this was the only condition which yielded growth-positive phenotypes.

Regarding the reviewer's third point, we agree that the original reference to Figure 2D was unclear. We have modified the text to make clearer references to Figures 2C and D (lines 172-175).

Reviewer 2 Comment 7
"As expected, for the pre-selection populations each of the 11 replicates was identified with a unique, highly enriched 'founder' insertion (except in one case, where the initial insertion occurred near a multi-copy ribosomal gene) (Fig. S4)." Unclear why this was expected. Presumably because only one copy was needed before selection, and so the founders usually had a single copy?

Response:
The reviewer is correct. The donor plasmid that was transformed into *E. coli* at the start of every evolution experiment contained an R6Ky origin of replication which relies on the *pir* gene product for plasmid replication. Since *E. coli* MG1655 and MDS42 do not contain the *pir* gene, they can only propagate after the transposon harboring the antibiotic resistance cassette is inserted into the host genome. A single genomic insertion is sufficient to confer cells with the ability to grow on selective media containing antibiotics. In theory, multiple insertions can occur in the initial founder cell, but the probability of this occurring is multiplicatively smaller than a single insertion.

We appreciate the reviewer for pointing out that this detail was not clear, and we have updated the text to more carefully explain why only a single insertion was expected (lines 169-172).

Reviewer 2 Comment 8
It would be good to note early on that many of the gene names that are appearing as enriched are actually the same locus. E.g. sdaA/yeaB/pabB in Fig 3F. Also plots including Fig 2E, SF S4 (etc). It was not immediately apparent that these likely often represent a single transposition event given that they have multiple gene names.

Response:

We agree with the reviewer that this fact is not always clear in the figures and captions. To address this, we have included annotated operon schematics in Figures 2F and 3E, consistent with Figures 4 and 5.

Reviewer 2 Comment 9
It may be helpful to the reader to note how the authors anticipate this system being used for metabolic optimization. For instance, I would expect that you wouldn't want to use this system in the microbial end product, but rather as a way to discover which modifications to make, which are then made in a less genetically burdensome way (e.g. adding a promoter, rather than a promoter-harboring transposon).

Response:
The reviewer raises an excellent point, and we agree that in any finalized strain for bioproduction or laboratory investigation, it would be advisable to ensure that the genomic/regulatory network changes were fixed rather than being dynamic. In this workflow, the transposon system would either be used to directly study the evolutionary process itself, or to generate candidate strains with desired phenotypes in a non-rational manner. If the end goal is a stable, standardized strain for a given industrial or laboratory application, any genomic changes identified in this screen would then be recreated via canonical genome engineering approaches to introduce the same regulatory elements but without the transposon, the transposase and any associated plasmids.

We have added text to the Discussion section of the paper to highlight this important clarification (lines 387-390).

Reviewer 2 Comment 10
Some of the fonts in the figures are very small and challenging to read (e.g. Fig 3B). It may help to print them out and gauge when printed on paper.

Response:
We thank the reviewer for this constructive feedback, and have taken the following steps to ensure that all subfigures are easily legible:

1. To address the issues with the size and legibility of Figure 3B raised in Reviewer 1's Comment 17 we have removed Figure 3D and enlarged Figure 3B.
2. We have enlarged several of the figures to reduce overcrowding, and reduced the number of different colors where possible.
3. We have ensured that all figure text is font size 6pt or greater.

Reviewer 2 Comment 11
The section "Benchmarking the platform against a natural transposon-mediated adaptive switch" could use some clarification. E.g. "Guided by previous work developing this phenomenon into an assay for MGE activity14," is an important part that could be expanded on and seems to form the basis for the benchmark. It's then somewhat confusing that only the Tn-pOUT constructs are able to confer a selective advantage.

Response:
We thank the reviewer for their comment. This important point was also raised by Reviewer 1 (Comment 12). In brief, within the time scale of our experiments, direct activation of *bglF/B/H* is

likely a favorable adaptation because these genes lack intragenic repressor binding sites. In contrast, *bglG* contains an intragenic H-NS repressor binding site which lowers the efficiency of transcription through *bglG* and leads to slower accumulation of the gene product. We have modified the main text to further elaborate on this hypothesis (lines 187-191). Given that other adaptations are possible, we have qualified our observations by explicitly stating that the mutations we observed arose "within the timescale of our experiment" (lines 172-173).

Reviewer 2 Comment 12
Many of the genomic insertion peak graphs have no Y-axis label (or even no Y axis). E.g. Fig 2G, 4GH.

Response:
We have added Y-axis labels to all of the insertion peak graphs to improve their interpretability.

Reviewer 2 Comment 13
Figure 1A, on the right hand side is somewhat confusing. I only figured it out after reading the whole paper (that the peaks in the Tn-seq are transposon insertion sites). Maybe label the transposon, the founder, the chromosome. Also, it is unclear where Scheme A was actually applied (in "Benchmarking the platform against a natural transposon-mediated adaptive switch"?). The L-Seriene experiment tested different concentrations after selection, but this was not a step-wise selection. The Scheme B comes in in Fig 5, and so could probably be saved for then. Might be best for Scheme A too to be in the appropriate section. Up to you though.

Response:
We have added labels to Figure 1A(iii), and we agree that this improves the interpretability of the schematics. Additionally, we have labeled schemes A and B with the corresponding figures that use these selection regimes.

Reviewer 2 Comment 14
Consider clarifying what is meant in the discussion: "In this context, the advantage of our transposon platform lies in its apparent ability to introduce insertions at multiple locations in a genome through changes in the copy number of the transposon, mimicking established models for the creation of co-regulated gene networks through MGE dispersal3." For instance, is this referring to eukaryotic transposable elements, or are there also compelling examples from prokaryotes?

Response:
We thank the reviewer for this excellent observation, and agree that this mode of dispersal of gene regulatory elements within a given genome is primarily attributed to eukaryotic systems. There are documented examples of repetitive, mobile regulatory sequences being distributed within prokaryotic genomes via DNA mobilization (e.g., the dispersal of REPINs via REP-associated tyrosine transposases, RNA regulatory element dispersal via ERICs, and the dispersal of promoter sequences via repetitive Correia elements in *Neisseria meningitidis*. However, the notion of (small) groups of genes forming co-regulated families via the insertion of homologous upstream promoter sequences as described in Figure 5 is arguably more analogous to the eukaryotic model for regulatory element dispersal. Our work therefore recapitulates a typically eukaryotic phenomenon in a prokaryotic context, and allows the user to create co-inducible phenotypes.

We have modified the Discussion to reflect this helpful suggestion from the reviewer (lines 374-375).

Reviewer 2 Comment 15
It is unclear what the simulation in Fig 3D adds vs Fig 3C. The EVOL-1 strain is clearly superior in L-Serine and has a dose-response relationship with concentration from Fig 3C.

Response:
We thank the reviewer for this constructive feedback, and agree that by removing this redundant figure we can free up additional space to address the issues with the size and legibility of Figure 3B raised in Reviewer 1's Comment 17 and Reviewer 2's Comment 10. We have therefore removed Figure 3D and enlarged Figure 3B.

Reviewer 3

General comments
The paper by English et al investigates the use of a new transposon system that allows for multi-loci genomic rearrangements through continuous evolution. The authors show that using their two-plasmid system, they can not only create convergent strains that lead to improved growth advantages in non-conventional media, but using barcodes are able to track the lineage of these strains. For the most part, the results shown support the statements by the authors, but modifications to the manuscript will need to be made before this can be published. There is a lack of detail in the paper that makes it hard for the reader to follow all the experiments and will need to be significantly expanded. More details should be added in the results section to increase the readability of this paper. Furthermore, while I believe this approach could be important for the synthetic biology community, there is a lack of background and comparisons to previous research that really help explain the novelty of this research. It would be good to cross reference to research that has previously been done in the GRN evolution field.

Main points
Reviewer 3 Comment 1
Overall, there is a lack of discussion about what the results mean. For example, what does it mean that the p-Tet system is leaky (Figure 1C)? Should this not be used? If the authors do not want to add this discussion to the results the discussion section needs to be expanded to fully explain the benefits and weaknesses of the system so that other users may decide whether they wish to use this technology.

Response:
We thank the reviewer for this comment. In lines with this comment and suggestions from the other reviewers, we have expanded the Results and Discussion sections to more comprehensively discuss results from each experiment. Additionally, we have included additional text on the limitations of our platform in the Discussion section (lines 362-368).

Regarding the reviewer's specific comment about the TetR/pTet-Tnpase system, we use the term "leaky" to describe a promoter that initiates intermediate or high levels of transcription while in the

uninduced state (e.g., without ATc). In the context of our platform, leaky expression leads to intermediate levels of transposase expression and transposition insertion frequencies even in the uninduced state. While the AraC/pBAD-Tnpase system had a less active uninduced state compared to the TetR/pTet-Tnpase system, we opted to use the TetR/pTet-Tnpase system due to the bio-orthogonal nature of its corresponding inducer. Importantly, although the TetR/pTet-Tnpase system is leaky, we are still able to increase transposase expression levels and, as a result, increase the transposon insertion frequency. We have now clarified this important detail in the text (lines 134-135).

Reviewer 3 Comment 2

Despite reading the paper carefully, I particularly found the first two sections of the results chapters difficult to understand. By the third section the authors seem to have found a better way to explain the results and why each experiment was done and what it intended to show. As this paper shows a new technology, the initial experiments to set up the system should be made as simple as possible to understand so that as many people are possible will be able to utilize this technique.

Response:

We thank the reviewer for their constructive comments. In line with these comments and similar ones from the other reviewers, we have made several changes which we believe improves the clarity of the manuscript:

1. We have added additional text explaining the implications of the results presented in Figure 1B (lines 101-111).
2. We have added an additional paragraph to the first results section that elaborates on how the transposon platform can facilitate directed evolution in bacterial genomes and provides a brief summary of all downstream experiments (lines 137-145).
3. We have added additional text to the results section discussing the modular transposon assembly and barcoding strategy introduced in Figure 4 (lines 250-263).
4. We have created a separate section for the results presented in Figure 5 and elaborated on the experimental workflow and important observations (lines 330-351).
5. We modified the Discussion section to note additional limitations of the platform (lines 362-368).
6. We have consolidated the abbreviations used for the helper plasmids and included a table summarizing all transposon parts and plasmids, along with the figures in which they appear.

Reviewer 3 Comment 3

It is unusual to have results in the materials and methods section that do not also appear in the main text of the paper (e.g., S2B and S2C) and particularly for the arbutin growth experiments the justifications that are included for the experiments in the material and methods are what I would expect to see in the rest of the main text. As a reader, it is difficult to flip back and forth between the materials and methods and the main text to understand the logic of the story.

Response:

We thank the reviewer for this comment, and we agree that several details regarding experimental design were underexplained in the initial submission. In line with the comment above (Reviewer 3's Comment 2), we have expanded the main text and discussion to further elaborate on several aspects of the experimental design and platform. Regarding the reviewer's specific comment about Appendix Figure S2, we have added additional text to elaborate on this experiment (lines 157-161).

Reviewer 3 Comment 4
The authors do not talk about whether the strains can be cured of the plasmid and the impact on the transposons being integrated into the genome can have on the stability of the strains over more generations. It would be of interest to see if, like with the lacI gene interruption, other beneficial modifications become apparent later. There is little information about what happens beyond 48 hours, do more mutations accumulate? Was there a reason the authors stopped at 48 hours?

Response:
We thank the reviewer for this comment, which is in line with Reviewer 2's Comment 9. In brief, our platform is best utilized as a screening tool to identify beneficial insertional mutations that confer a fitness advantage in selective environments. Once these mutations are identified, they could then be reintroduced using canonical genome engineering approaches in order to generate a more stable strain applicable for bioproduction. We have now added this important point to the Discussion section (lines 387-390).

In addition, we appreciate the reviewer's interest in conducting longer-term evolution experiments with our platform. We agree that it would be fascinating to conduct longer-term evolution experiments, as this could reveal additional, rare adaptations to our selections. Unfortunately, these experiments are difficult to perform in manual, plate-based experiments with small samples volumes due to concerns with media evaporation. In future studies, these longer-term evolution experiments would ideally be performed using continuous culture systems.

Reviewer 3 Comment 5
I would be interested to know whether the same mutations that were found for the adaptation to different carbons sources have been used before, even by design or through random mutagenesis in previous adaptation experiments.

Response:
We thank the reviewer for this comment. Table 2 includes references to previous reports of adaptive mutations which enable *E. coli* to grow on the different carbon sources used in our study. For cases not already discussed, we have modified Table 2 and the main text to further elaborate on the similarities and differences between the adaptive mutations observed in our study and previous reports. We provide a brief summary below:

1. Growth on arbutin is naturally enabled by IS1/IS5-mediated disruption of operator sites upstream of *bglG*, which increases expression of *bglG* and the downstream structural genes *bglF*, *bglB*, and *bglH*. In our study, we instead observed the direct activation of *bglF*, *bglB*, and *bglH*.
2. Growth on L-serine is naturally enabled by mutations in transcriptional regulators, such as *cpxA*, which lead to a pleiotropic phenotype including the increased expression of *sdaA*. We also observed the inactivation of *cpxA* in cases where the transposon harbored an inducible promoter without the corresponding inducer. Conversely, in cases where the transposon harbored either a constitutive promoter or an inducible promoter with the corresponding inducer, we observed the direct activation of *sdaA*.
3. Growth on ß-methyl-D-glucoside has been observed in *E. coli* strains displaying enhanced expression and activity of the *bglA* gene product via mutations in the promoter and coding sequence, respectively. We also identified insertions upstream of *bglA* which, in cases where

the transposon harbored an inducible promoter with the corresponding inducer, presumably increased expression of *bglA*. Conversely, in cases where the transposon harbored an inducible promoter without the corresponding inducer, we observed insertions in the *nudH* gene. Inactivation of *nudH* has been reported to indirectly increase *bglA* expression.

4. Wetmore *et al.*[8] previously performed an RB-Tn-Seq experiment with *E. coli* growing on various carbon sources, including glycyl-L-glutamic acid – their screen associated the inactivation of *sspA* with enhanced growth on glycyl-L-glutamic acid. Our screen also identified *sspA* mutants as being able to grow using glycyl-L-glutamic as the sole carbon source.

Reviewer 3 Comment 6

It's hard to follow what is happening in Figure 1B as the reference to it is only fleeting and the figure legend does not do enough to explain what is occurring.

Response:

We thank the reviewer for this comment, which is in line with Reviewer 1's Comment 4 and Reviewer 2's Comment 4. We have added additional text to the results section elaborating on the findings of this experiment and implications for our engineered platform (lines 101-111). In line with Reviewer 2's Comment 1, we have also modified the title for Figure 1B to more clearly emphasize the main conclusion drawn from this experiment.

Reviewer 3 Comment 7

For materials and methods discussing the arbutin assay, I feel that Figure S2B-E are results and should be in the main text and not in the materials and methods section.

Response:

We thank the reviewer for their constructive feedback. We have included discussion of Appendix Figure S2B-E in the results section of the main text (lines 157-161). Due to space limitations, we have decided to keep Figure S2B-E in the Appendix.

Reviewer 3 Comment 8

Figure legend 1D, please comment on the statistics of this figure. What do the dotted lines mean vs the solid lines. In the text there is no comment on the distribution of the mutations on the colonies. For instance, in Tn, in the second transformation there appear to be a lot of colonies that barely grow, would these types of different distributions be expected?

Response:

We thank the reviewer for this comment. The solid lines correspond to the median and the dotted lines correspond to the quartiles.

Additionally, while it is difficult to predict the distribution of growth phenotypes following selection on D-cycloserine, we are not surprised that the growth profiles are heterogeneous. There are multiple reasons for this. First, early-arising mutants have likely been in stationary phase for an extended period of time which would result in some cell death and a decrease in OD600. Second, the exact location of the transposon insertion within the *cycA* likely impacts bacterial growth. Third, there may be some cells that accrue additional secondary transposon insertions in the genome during the outgrowth on non-selective media, which could further impact growth during the D-cycloserine selection.

Reviewer 3 Comment 9
Figure 4B, there is no discussion in the text about how the contamination occurs and what the impact of this might be. This is now referenced in regard to Fig S13 but further through the manuscript, but should be discussed upon first mention.

Response:
We agree with the reviewer on the importance of discussing the contamination event. We have added text to the results section elaborating on possible sources of contamination and how the computational identification of contamination prevented this event from impacting our interpretation of the results. (lines 292-299).

Reviewer 3 Comment 10
The figures in S1B and C are not discussed in the text and should be explained in the manuscript. Figure S1 was referred to in results section constructing a self-propagating transposon mutagenesis platform, paragraph 1, but this is not in relation to arbutin as far as I am aware and yet the figure legend specifically refers to this. Please either correct the figure legend or change where this figure is referenced in the manuscript.

Response:
We thank the reviewer for raising this point. We agree that the original reference to Appendix Figure S1 was inappropriate, as this supplementary figure is related to the arbutin evolution experiment. We have moved this reference to the arbutin evolution results section (line 159).

Reviewer 3 Comment 11
Figure S2A, this figure is very confusing in the way that it is presented. What is the difference between the grey writing on the arrows and the grey writing at the bottom of the experiment. Are all the first steps consistent regardless of the strains and only the last growth in the selection +/- inducer different between the strains?

Response:
We agree with the reviewer that the significance of the gray text was not clear in the figure, and have removed it. Additionally, we have made changes to the schematic text to emphasize the two different experimental workflows used to generate the data presented.

Reviewer 3 Comment 12
Figure S9, it is hard to understand the purpose of this experiment as it is not discussed in detail in the text.

Response:
The importance of this experiment is twofold:

1. First, to validate the previously-described inducible promoter systems and establish upper limits on the potential gene activation levels achievable with these systems.
2. Second, to verify that the promoter systems retain function when activating genes via transcription across the sequence of the transposon end. This second point is important as the transposon end sequence may directly impact transcription, and/or contain sequences that affect RNA structure and function.

We agree with the reviewer that the text does not contain sufficient explanation to highlight these reasons, and have made changes accordingly to the manuscript (lines 255-258). Also, the original Appendix Figure S9 is now Appendix Figure S8.

Reviewer 3 Comment 13
Figure S16, why were no cells grown in the final passage, does this imply that the selection has not been as successful? Please expand on this in the main text.
Yes none grew, this is shown in Fig. 4B

Response:
We thank the reviewer for this comment. Indeed, none of the cells containing the RB-TnV2 transposon variant grew on selective media. This is summarized in Figure 4B, where the only growth observed in the RB-TnV2 lineages was the result of a contamination event. We have now highlighted this contamination event in the main text (lines 294-298).

Minor points
Reviewer 3 Comment 14
Please provide page numbers and line numbers to make it easier for reviewers to comment on specifics of the paper.

Response:
We have added page numbers and line numbers to the revised manuscript.

Reviewer 3 Comment 15
Figure 2A, please explain what site B is in the figure legend.

Response:
To clarify the significance of site B, we have added text to the figure legend (lines 460-461).

Reviewer 3 Comment 16
Materials and methods, under transformation and D-cyloserine assays, the authors are referring to Figure 1D and not 1C as written.

Response:
We thank the reviewer for highlighting this error, and have corrected the manuscript accordingly.

References for all reviewer responses:

1. Baisa, G., Stabo, N. J. & Welch, R. A. Characterization of Escherichia coli d-Cycloserine Transport and Resistant Mutants. J Bacteriol 195, 1389–1399 (2013).
2. Fehér, T., Cseh, B., Umenhoffer, K., Karcagi, I. & Pósfai, G. Characterization of cycA mutants of Escherichia coli: An assay for measuring in vivo mutation rates. Mutation Research/Fundamental and Molecular Mechanisms of Mutagenesis 595, 184–190 (2006).
3. Pósfai, G. et al. Emergent Properties of Reduced-Genome Escherichia coli. Science 312, 1044–1046 (2006).
4. Maslowska, K. H., Makiela-Dzbenska, K. & Fijalkowska, I. J. The SOS system: A complex and tightly regulated response to DNA damage. Environmental and Molecular Mutagenesis 60, 368–384 (2019).
5. Hall, B. G. Activation of the bgl operon by adaptive mutation. Molecular Biology and Evolution 15, 1–5 (1998).
6. Schnetz, K. & Rak, B. IS5: a mobile enhancer of transcription in Escherichia coli. Proc Natl Acad Sci U S A 89, 1244–1248 (1992).
7. Dole, S., Nagarajavel, V. & Schnetz, K. The histone-like nucleoid structuring protein H-NS represses the Escherichia coli bgl operon downstream of the promoter. Molecular Microbiology 52, 589–600 (2004).
8. Wetmore, K. M. et al. Rapid Quantification of Mutant Fitness in Diverse Bacteria by Sequencing Randomly Bar-Coded Transposons. mBio 6, e00306-15 (2015).

Thank you for sending us your revised manuscript. We have now heard back from the three reviewers who were asked to evaluate your revised study. As you will see below, the reviewers are satisfied with the performed revisions and support publication. However, they list some remaining issues, which we would ask you to fix in a minor revision. Importantly, as reviewer #3 points out, all information about methods, statistical analyses, data etc. should be included in the manuscript text. Essential information regarding the performed experiments and the related data and results should be included in the main text and not only in the point-by-point response to the reviewers.

We would also ask you to address some remaining editorial issues listed below.

Reviewer #1:

The authors have adequately addressed all of my major comments. Importantly, the re-organized manuscript is more focused and much easier to follow. Overall, they've developed an exciting new approach for massively parallel analysis of evolved bacterial populations, using sequential insertions of engineered and functionalized transposons. I anticipate the wide adoption of their tool by research groups investigating general evolutionary questions and groups with specific engineering targets (like tolerance phenotypes).

Upon re-reading the manuscript, I did have a few minor edits:

Line 103: States that MG1655 cells are used in the Figure 1B data, but the figure legends says MDS42 cells (Line 433).

Lines 474-479: In Figure 2GH, it would be good to specify that the data is from 2/11 lines.

In Figure 4C, the top panel should be labeled +ATc and the bottom panel -ATc.

Reviewer #2:

I commend the authors on their thorough addressing of the points we raised in the last round of review. The clarity of the manuscript is greatly improved. Only two minor issues remain.

Line 176: Consider changing "initial-" to "start-" since "initial-point" is not commonly used, and Fig 2E uses "start-point".

Figure 2H is confusing and cannot be interpreted without the caption (and is even unclear with it). What are the colours? What is the top vs bottom panel? Is this real data (not clear from the caption)? Presumably it is real data given the zoom in for 2G. The dashed arrow does not indicate "transposon movement from their initial location", but preservation of the initial transposon insertion event that doesn't disappear after selection. Maybe explicitly labeling initial insertion locations would be good, especially given the close proximity of the purple transposon's initial site to the bgl operon. Labeling the Bgl operon more clearly could be helpful too (the gray zoom triangle to 2G does not make this clear). In this example it's also unclear why the purple transposon moves next to the cyan transposon's initial location. Is there some additional benefit there? Without explanation, the "take home" seems to be that they both move next to where the other one was. Presumably the cyan one moves to the purple one because the purple one happens to be next to the bgl operon, but the purple moving next to cyan is just confusing. Fig 3F has some of the same issues, but the sdaA convergence is clearer due to the inset figure.

Reviewer #3:

The amendments by English et al. to their paper on a new transposon system for rewiring genomic networks have significantly improved the readability of the paper. However, there are still many instances where the experiments are performed, the data shown, but the results of the paper not explained. The response to reviewers' comments are helpful in guiding the reader

through the data; however, not all of the information has been translated in significant detail in the manuscript. If the reviewers have such comments, I imagine readers do too. It would be good to guide the readers in the same way that the authors have for the reviewers. In particular, although work has been done to expand on section 1, "constructing a self-propagating transposon mutagenesis platform" this is still difficult to understand on first reading.

Figure 1D: What is the difference between Tn and Tn[GFP] is and are both represented? If it is, as is possible, to represent that different strains and even those carrying an extra gene, are able to be replicated accordingly, then please discuss this.

Table 1: the addition of this table is beneficial; however, the authors have indicated that there is a difference between TetR/p-Tet-Tnpase (Chlor) and TetR/p-Tet-Tnpase (Carb) but even on first mention of these plasmids (page 4) this differentiation was not used. This table is only beneficial if it is a true reflection of what is written in the manuscript.

Page 4, line 149: It would be helpful if when first describing the experiment the difference between Tn and Tn-pOUT was described in reference to figure 2A, rather than coming at the end of the first paragraph.

Figure S7: it has not been clearly explained why both chloramphenicol and ampicillin/carbenicillin are both used in experiments. Would it be expected that these would differ?

In accordance with other suggestions from reviewers the authors have now included statistical analysis on some of their graphs but the methods describing these are not available in the materials and methods, or I missed them.

Manuscript ID: MSB-2022-11398R
Title: A self-propagating, barcoded transposon system for the dynamic rewiring of genomic networks

Reviewer 1

General comments
The authors have adequately addressed all of my major comments. Importantly, the re-organized manuscript is more focused and much easier to follow. Overall, they've developed an exciting new approach for massively parallel analysis of evolved bacterial populations, using sequential insertions of engineered and functionalized transposons. I anticipate the wide adoption of their tool by research groups investigating general evolutionary questions and groups with specific engineering targets (like tolerance phenotypes).

Upon re-reading the manuscript, I did have a few minor edits:

Minor points
Reviewer 1 Comment 1
Line 103: States that MG1655 cells are used in the Figure 1B data, but the figure legends says MDS42 cells (Line 433).

Response:
We thank the reviewer for highlighting this discrepancy. The experiment in Figure 1B was conducted in *E. coli* MG1655 cells. This was erroneously described as *E. coli* MDS42 in the legend for Figure 1B. We have now corrected the figure legend (line 1202).

We have carefully reviewed the text and have confirmed that there are no more erroneous mentions of *E. coli* MG1655 or MDS42.

Reviewer 1 Comment 2
Lines 474-479: In Figure 2GH, it would be good to specify that the data is from 2/11 lines.

Response:
We thank the reviewer for their constructive comments. We now indicate that the same two, representative traces (out of eleven replicates) are shown in Figures 2G and 2H (lines 1246-1247 and lines 1251-1253).

Also, in accordance with Reviewer 2's Comment 2, we have added additional details to Figures 2G and 2H and their corresponding legends (lines 1244-1255) in order to facilitate interpretation of these plots.

Reviewer 1 Comment 3
In Figure 4C, the top panel should be labeled +ATc and the bottom panel -ATc.

Response:
We thank the reviewer for pointing out this discrepancy between Figure 4C and its legend. The orientation described in the figure is correct: the top panel corresponds to cells incubated without

ATc and the bottom panel corresponds to cells incubated with ATc. This was incorrectly described in the figure legend, which we have now corrected (line 1295).

Reviewer 2

General comments
I commend the authors on their thorough addressing of the points we raised in the last round of review. The clarity of the manuscript is greatly improved. Only two minor issues remain.

Minor points
Reviewer 2 Comment 1
Line 176: Consider changing "initial-" to "start-" since "initial-point" is not commonly used, and Fig 2E uses "start-point".

Response:
We thank the reviewer for their suggestion. We have now made this change (line 204).

Reviewer 2 Comment 2
Figure 2H is confusing and cannot be interpreted without the caption (and is even unclear with it). What are the colours? What is the top vs bottom panel? Is this real data (not clear from the caption)? Presumably it is real data given the zoom in for 2G. The dashed arrow does not indicate "transposon movement from their initial location", but preservation of the initial transposon insertion event that doesn't disappear after selection. Maybe explicitly labeling initial insertion locations would be good, especially given the close proximity of the purple transposon's initial site to the bgl operon. Labeling the Bgl operon more clearly could be helpful too (the gray zoom triangle to 2G does not make this clear). In this example it's also unclear why the purple transposon moves next to the cyan transposon's initial location. Is there some additional benefit there? Without explanation, the "take home" seems to be that they both move next to where the other one was. Presumably the cyan one moves to the purple one because the purple one happens to be next to the bgl operon, but the purple moving next to cyan is just confusing. Fig 3F has some of the same issues, but the sdaA convergence is clearer due to the inset figure.

Response:
We agree that the lack of description for Figures 2G and 2H make them difficult to interpret. Figures 2G and 2H are real data which are meant to demonstrate the convergence on transposon insertion upstream of *bglF* as evidence for transposon-mediated adaptation to arbutin. We showed two representative replicates (the teal and purple lines) to demonstrate that, in addition to insertion upstream of *bglF*, the transposons preserve their initial insertion and may also insert at additional locations throughout the genome.

To facilitate interpretation, we have added more annotations to the figure and have included additional explanatory text to the figure legend. In summary, we have made the following changes:

1. We have included more description in the figure legend (lines 1244-1255). This new legend more explicitly describes the information shown in the plots (e.g., colors, dashed versus solid lines) and how the plots were generated from the sequencing data.
2. We have made the title for Figure 2G more descriptive. It now includes the take-home message that we see convergence on “transposon insertion upstream of *bglF*”.

3. We have included more annotations for Figure 2H that highlight: (i) the top and bottom panels correspond to start- and end-point traces, respectively; (ii) the start-point peaks represent the initial founder insertions; the end-point peaks demonstrate preservation of the initial insertion in addition to remobilization to other locations throughout the genome; and (iii) all endpoint replicates contain an adaptive insertion within the *bgl* operon. We have added similar annotations to Figure 3F.

We thank the reviewer for their feedback, which we believe has helped us significantly improve the interpretability of Figures 2G, 2H and 3F.

Reviewer 3

General comments

The amendments by English et al. to their paper on a new transposon system for rewiring genomic networks have significantly improved the readability of the paper. However, there are still many instances where the experiments are performed, the data shown, but the results of the paper not explained. The response to reviewers' comments are helpful in guiding the reader through the data; however, not all of the information has been translated in significant detail in the manuscript. If the reviewers have such comments, I imagine readers do too. It would be good to guide the readers in the same way that the authors have for the reviewers. In particular, although work has been done to expand on section 1, "constructing a self-propagating transposon mutagenesis platform" this is still difficult to understand on first reading.

Response:

We thank the reviewer for their helpful comments, which we believe has helped us substantially improve the clarity of our text. We also appreciate the reviewer for pointing out that there were portions of the main text that could be further improved. In addition to incorporating the reviewer's specific suggestions below, we have made the following changes to further improve the clarity of our text:

1. We have added more description to the first Results subsection entitled "Constructing a self-propagating transposon mutagenesis platform". In particular, we have explained our reasoning for testing the two transposon variants and the conclusions derived from the consistent performance between the two variants (lines 125-128; lines 134-136; lines 148-150). Additionally, we have added more description to the D-cycloserine experiment presented in Figure 1D (lines 141-143; lines 150-153). This includes emphasizing the importance of the parental *E. coli* MDS42 strain as a negative control throughout the experiments in this paper (lines 157-161).
2. In accordance with the comments from Reviewer 2, we have added more detail to Figures 2G, 2H, and 3F and their corresponding legends (lines 1244-1255).
3. We have added additional description for the results presented in the arbutin evolution experiment (line 191-193; lines 194-195; lines 210-212; lines 222-224).
4. We have added additional, relevant experimental details to the experiments presented in Figure 3 (lines 240-245). We also include an explanation for repeating the L-serine evolution experiment with two helper plasmid variants (line 275-277).
5. We have added more description for the results presented in Figures 4B and 4C (lines 323-337). Specifically, we provide additional details explaining the results presented in Figure 4B and explicitly discuss the changes in OD600 throughout the experiment shown in Figure

4C. Finally, we emphasize the relationship between the number of founder colonies and the theoretical transposon barcode library size (lines 344-347).

Minor points
Reviewer 3 Comment 1
Figure 1D: What is the difference between Tn and Tn[GFP] is and are both represented? If it is, as is possible, to represent that different strains and even those carrying an extra gene, are able to be replicated accordingly, then please discuss this.

Response:
We thank the reviewer for their constructive comments. The Tn variant only contains a kanamycin resistance gene whereas the Tn[GFP] variant contains an additional gene encoding for an mNeonGreen fluorescent reporter. We have now added additional text describing the difference between these two transposon variants (lines 126-128).

Our intention was to demonstrate that the transposons are still capable of undergoing adaptive insertions in the host genome when they are functionalized with synthetic cargo. We have now clarified this in lines 134-136 and lines 148-150.

Reviewer 3 Comment 2
Table 1: the addition of this table is beneficial; however, the authors have indicated that there is a difference between TetR/p-Tet-Tnpase (Chlor) and TetR/p-Tet-Tnpase (Carb) but even on first mention of these plasmids (page 4) this differentiation was not used. This table is only beneficial if it is a true reflection of what is written in the manuscript.

Response:
To avoid confusion, we now explicitly refer to the TetR/pTet-transposase with chloramphenicol resistance as "TetR/pTet-transposase (cam^R)" (lines 123, 132, and 155). We use the abbreviation "cam^R" (chloramphenicol resistance) to remain consistent between Figure 1B and the main text.

Reviewer 3 Comment 3
Page 4, line 149: It would be helpful if when first describing the experiment the difference between Tn and Tn-pOUT was described in reference to figure 2A, rather than coming at the end of the first paragraph.

Response:
We thank the reviewer for their suggestion. Our initial reference to Figure 2A focuses on the *bgl* operon architecture and the natural mode of insertion-mediated adaptation to arbutin. Conversely, the Tn and Tn-pOUT transposon variants are specific to our experimental setup, which is why we chose to describe them at the end of the first paragraph.

Nonetheless, the reviewer's comment points out that our rationale for using both the Tn and Tn-pOUT variants was not clear. We have now added additional text explaining our reasoning for using both transposon variants (lines 191-193).

Reviewer 3 Comment 4

Figure S7: it has not been clearly explained why both chloramphenicol and ampicillin/carbenicillin are both used in experiments. Would it be expected that these would differ?

Response:
We do not expect the results between the chloramphenicol and carbenicillin resistance helper plasmids to differ. Our intention was to demonstrate the reproducibility of the L-serine evolution experiment, regardless of the antibiotic selection marker on the helper plasmids. We have now described this on lines 275-277.

Reviewer 3 Comment 5
In accordance with other suggestions from reviewers the authors have now included statistical analysis on some of their graphs but the methods describing these are not available in the materials and methods, or I missed them.

Response:
We have included descriptions for all statistical tests in both the main text and Methods section.

1. The statistical test performed in Figure 1D is described on lines 1214-1216 in the main text and lines 572-575 in the methods.
2. The statistical test performed to produce the volcano plots in Figures 2F, 3F, and 4F/H are described on lines 1242-1244, 1275-1278, and 1315-1317 in the main text, respectively. This test is also described in lines 751-754 in the methods. The volcano plots in the supplemental figures (Appendix Figures 6 and 20-22) also contain an accompanying description in their figure legends.

Thank you again for sending us your revised manuscript. We are now satisfied with the modifications made and I am pleased to inform you that your paper has been accepted for publication.

EMBO Press Author Checklist

Corresponding Author Name: James J. Collins	
Journal Submitted to: Molecular Systems Biology	
Manuscript Number: MSB-2022-11398	

USEFUL LINKS FOR COMPLETING THIS FORM
The EMBO Journal - Author Guidelines
EMBO Reports - Author Guidelines
Molecular Systems Biology - Author Guidelines
EMBO Molecular Medicine - Author Guidelines

Reporting Checklist for Life Science Articles (updated January
This checklist is adapted from Materials Design Analysis Reporting (MDAR) Checklist for Authors. MDAR establishes a minimum set of requirements in transparent reporting in the life sciences (see Statement of Task: 10.31222/osf.io/9sm4x). Please follow the journal's guidelines in preparing your
Please note that a copy of this checklist will be published alongside your article.

Abridged guidelines for figures
1. Data
The data shown in figures should satisfy the following conditions:
- the data were obtained and processed according to the field's best practice and are presented to reflect the results of the experiments in an accurate and unbiased manner.
- ideally, figure panels should include only measurements that are directly comparable to each other and obtained with the same assay.
- plots include clearly labeled error bars for independent experiments and sample sizes. Unless justified, error bars should not be shown for technical
- if n<5, the individual data points from each experiment should be plotted. Any statistical test employed should be justified.
- Source Data should be included to report the data underlying figures according to the guidelines set out in the authorship guidelines on Data

2. Captions
Each figure caption should contain the following information, for each panel where they are relevant:
- a specification of the experimental system investigated (eg cell line, species name).
- the assay(s) and method(s) used to carry out the reported observations and measurements.
- an explicit mention of the biological and chemical entity(ies) that are being measured.
- an explicit mention of the biological and chemical entity(ies) that are altered/varied/perturbed in a controlled manner.
- the exact sample size (n) for each experimental group/condition, given as a number, not a range;
- a description of the sample collection allowing the reader to understand whether the samples represent technical or biological replicates (including how many animals, litters, cultures, etc.).
- a statement of how many times the experiment shown was independently replicated in the laboratory.
- definitions of statistical methods and measures:
  - common tests, such as t-test (please specify whether paired vs. unpaired), simple $\chi 2$ tests, Wilcoxon and Mann-Whitney tests, can be unambiguously identified by name only, but more complex techniques should be described in the methods section;
  - are tests one-sided or two-sided?
  - are there adjustments for multiple comparisons?
  - exact statistical test results, e.g., P values = x but not P values < x;
  - definition of 'center values' as median or average;
  - definition of error bars as s.d. or s.e.m.

Please complete ALL of the questions below.
Select "Not Applicable" only when the requested information is not relevant for your study.

Materials

Newly Created Materials	Information included in the manuscript?	In which section is the information available? (Reagents and Tools Table, Materials and Methods, Figures, Data Availability Section)
New materials and reagents need to be available; do any restrictions apply?	Yes	No restrictions apply. New materials are listed in Materials and Methods, Data Availability

Antibodies	Information included in the manuscript?	In which section is the information available? (Reagents and Tools Table, Materials and Methods, Figures, Data Availability Section)
For **antibodies** provide the following information: - Commercial antibodies: RRID (if possible) or supplier name, catalogue number and or/clone number - Non-commercial: RRID or citation	Not Applicable	

DNA and RNA sequences	Information included in the manuscript?	In which section is the information available? (Reagents and Tools Table, Materials and Methods, Figures, Data Availability Section)
**Short novel DNA or RNA including primers, probes**: provide the sequences.	Yes	Appendix Table S1

Cell materials	Information included in the manuscript?	In which section is the information available? (Reagents and Tools Table, Materials and Methods, Figures, Data Availability Section)
**Cell lines:** Provide species information, strain. Provide accession number in repository **OR** supplier name, catalog number, clone number, and/**OR** RRID.	Not Applicable	
**Primary cultures:** Provide species, strain, sex of origin, genetic modification status.	Not Applicable	
Report if the cell lines were recently **authenticated** (e.g., by STR profiling) and tested for mycoplasma contamination.	Not Applicable	

Experimental animals	Information included in the manuscript?	In which section is the information available? (Reagents and Tools Table, Materials and Methods, Figures, Data Availability Section)
**Laboratory animals or Model organisms:** Provide species, strain, sex, age, genetic modification status. Provide accession number in repository **OR** supplier name, catalog number, clone number, **OR** RRID.	Not Applicable	
**Animal observed in or captured from the field:** Provide species, sex, and age where possible.	Not Applicable	
Please detail **housing and husbandry conditions**.	Not Applicable	

Plants and microbes	Information included in the manuscript?	In which section is the information available? (Reagents and Tools Table, Materials and Methods, Figures, Data Availability Section)
**Plants:** provide species and strain, ecotype and cultivar where relevant, unique accession number if available, and source (including location for collected wild specimens).	Not Applicable	
**Microbes:** provide species and strain, unique accession number if available, and source.	Yes	Materials and Methods

Human research participants	Information included in the manuscript?	In which section is the information available? (Reagents and Tools Table, Materials and Methods, Figures, Data Availability Section)
If collected and within the bounds of privacy constraints report on age, sex and gender or ethnicity for all study participants.	Not Applicable	

Core facilities	Information included in the manuscript?	In which section is the information available? (Reagents and Tools Table, Materials and Methods, Figures, Data Availability Section)
If your work benefited from core facilities, was their service mentioned in the acknowledgments section?	Not Applicable	

Design

Study protocol	Information included in the manuscript?	In which section is the information available? (Reagents and Tools Table, Materials and Methods, Figures, Data Availability Section)
If study protocol has been **pre-registered, provide DOI in the manuscript**. For clinical trials, provide the trial registration number **OR** cite DOI.	Not Applicable	
Report the **clinical trial registration number** (at ClinicalTrials.gov or equivalent), where applicable.	Not Applicable	

Laboratory protocol	Information included in the manuscript?	In which section is the information available? (Reagents and Tools Table, Materials and Methods, Figures, Data Availability Section)
Provide DOI OR other citation details if **external detailed step-by-step protocols** are available.	Yes	Provided in Materials and Methods, and citations

Experimental study design and statistics	Information included in the manuscript?	In which section is the information available? (Reagents and Tools Table, Materials and Methods, Figures, Data Availability Section)
Include a statement about **sample size** estimate even if no statistical methods were used.	Not Applicable	
Were any steps taken to minimize the effects of subjective bias when allocating animals/samples to treatment (e.g. **randomization procedure**)? If yes, have they been described?	Not Applicable	
Include a statement about **blinding** even if no blinding was done.	Not Applicable	
Describe **inclusion/exclusion criteria** if samples or animals were excluded from the analysis. Were the criteria pre-established?<br><br>If sample or data points were omitted from analysis, report if this was due to attrition or intentional exclusion and provide justification.	Not Applicable	
For every figure, are **statistical tests** justified as appropriate? Do the data meet the assumptions of the tests (e.g., normal distribution)? Describe any methods used to assess it. Is there an estimate of variation within each group of data? Is the variance similar between the groups that are being statistically compared?	Not Applicable	

Sample definition and in-laboratory replication	Information included in the manuscript?	In which section is the information available? (Reagents and Tools Table, Materials and Methods, Figures, Data Availability Section)
In the figure legends: state number of times the experiment was **replicated** in laboratory.	Yes	Figure captions
In the figure legends: define whether data describe **technical or biological replicates**.	Yes	Figure captions

Ethics

Ethics	Information included in the manuscript?	In which section is the information available? (Reagents and Tools Table, Materials and Methods, Figures, Data Availability Section)
Studies involving **human participants**: State details of **authority granting ethics approval** (IRB or equivalent committee(s), provide reference number for approval.	Not Applicable	
Studies involving **human participants**: Include a statement confirming that **informed consent** was obtained from all subjects and that the experiments conformed to the principles set out in the WMA Declaration of Helsinki and the Department of Health and Human Services Belmont Report.	Not Applicable	
Studies involving **human participants:** For publication of **patient photos**, include a statement confirming that consent to publish was obtained.	Not Applicable	
Studies involving experimental **animals**: State details of **authority granting ethics approval** (IRB or equivalent committee(s), provide reference number for approval. Include a statement of compliance with ethical regulations.	Not Applicable	
Studies involving **specimen and field samples:** State if relevant **permits** obtained, provide details of authority approving study; if none were required, explain why.	Not Applicable	

Dual Use Research of Concern (DURC)	Information included in the manuscript?	In which section is the information available? (Reagents and Tools Table, Materials and Methods, Figures, Data Availability Section)
Could your study fall under dual use research restrictions? Please check biosecurity documents and list of **select agents and toxins** (CDC): https://www.selectagents.gov/sat/list.htm	Not Applicable	
If you used a select agent, is the security level of the lab appropriate and reported in the manuscript?	Not Applicable	
If a study is subject to dual use research of concern regulations, is the name of the **authority granting approval and reference number** for the regulatory approval provided in the manuscript?	Not Applicable	

Reporting

The MDAR framework recommends adoption of discipline-specific guidelines, established and endorsed through community initiatives. Journals have their own policy about requiring specific guidelines and recommendations to complement MDAR.

Adherence to community standards	Information included in the manuscript?	In which section is the information available? (Reagents and Tools Table, Materials and Methods, Figures, Data Availability Section)
State if relevant guidelines or checklists (e.g., **ICMJE, MIBBI, ARRIVE, PRISMA**) have been followed or provided.	Not Applicable	
For **tumor marker prognostic studies,** we recommend that you follow the **REMARK** reporting guidelines (see link list at top right). See author guidelines, under 'Reporting Guidelines'. Please confirm you have followed these guidelines.	Not Applicable	
For **phase II and III randomized controlled trials**, please refer to the **CONSORT** flow diagram (see link list at top right) and submit the CONSORT checklist (see link list at top right) with your submission. See author guidelines, under 'Reporting Guidelines'. Please confirm you have submitted this list.	Not Applicable	

Data Availability

Data availability	Information included in the manuscript?	In which section is the information available? (Reagents and Tools Table, Materials and Methods, Figures, Data Availability Section)
Have **primary datasets** been deposited according to the journal's guidelines (see 'Data Deposition' section) and the respective accession numbers provided in the Data Availability Section?	Yes	Materials and Methods, Data Availability Section
Were **human clinical and genomic datasets** deposited in a public access-controlled repository in accordance to ethical obligations to the patients and to the applicable consent agreement?	Not Applicable	
Are **computational models** that are central and integral to a study available without restrictions in a machine-readable form? Were the relevant accession numbers or links provided?	Not Applicable	
If publicly available data were reused, provide the respective **data citations in the reference list**.	Not Applicable	
